# Catalytic Nucleophilic Fluorination of Secondary and Tertiary Propargylic Electrophiles with a Copper–N-Heterocyclic Carbene Complex

**DOI:** 10.1002/anie.201506882

**Published:** 2015-09-25

**Authors:** Li-Jie Cheng, Christopher J Cordier

**Affiliations:** Department of Chemistry, Imperial College London South Kensington, London, SW7 2AZ (UK) E-mail: ccordier@imperial.ac.uk

**Keywords:** alkynes, carbene ligands, copper, fluorine, homogeneous catalysis

## Abstract

A catalytic method for the nucleophilic fluorination of propargylic electrophiles is described. Our protocol involves the use of a Cu(NHC) complex as the catalyst and is suitable for the preparation of secondary and tertiary propargylic fluorides without the formation of isomeric fluoroallenes. Preliminary mechanistic investigations suggest that fluorination proceeds via copper acetylides and that cationic species are involved.

Compounds containing C–F bonds are of vital importance to the pharmaceutical[1] and agrochemical industries,[2] positron-emission tomography,[3] and materials science.[4] Catalytic fluorination has been the focus of many investigations,[5] in which either electrophilic or nucleophilic fluorine sources have been used.[6] Despite these significant advances, catalytic nucleophilic fluorination remains a challenge, particularly at C(sp^3^) centers.[7] The high charge density of unsolvated fluoride anions imparts high nucleophilicity but also strong basicity,[8] thus enabling elimination pathways to alkenes. Hydrogen bonding to fluoride anions by protic solvents limits the formation of alkene by-products owing to reduced basicity[9] but significantly reduces fluoride nucleophilicity. This dichotomy represents a unique challenge for catalysis, and the catalytic nucleophilic fluorination of comparatively simple non-activated primary alkyl electrophiles is still relatively underdeveloped.[10] Strategies for installing fluoride substituents into organic molecules equipped with functional groups for subsequent synthetic elaboration are of notable utility. To meet this objective, transition-metal-catalyzed methods for the nucleophilic fluorination of allylic electrophiles, with Pd,[11] Ir,[12] Rh,[13] or Cu[14] complexes as catalysts, have received considerable attention [Eq. (1)].


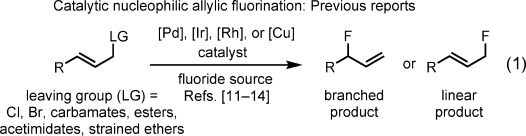


These approaches exploit the electrophilicity of metal–π-allyl intermediates and have been elegantly engineered to overcome significant challenges, including elimination to form dienes, the reversibility of C–F bond formation,[15] and regioselectivity (formation of branched or linear allylic fluorides). By contrast, catalytic methods for preparing propargylic fluorides remain underrepresented.[16] Propargylic fluorides are important motifs in biologically active compounds[17] and are used as synthetic precursors to fluorinated analogues of fluoroglycosides and antiviral, antifungal, and anticancer agents.[18] At present, reagent-based protocols for the dehydroxyfluorination[19] of propargylic alcohols remain the state-of-the-art methods for the preparation of propargylic fluorides, and, to the best of our knowledge, catalytic nucleophilic fluorination reactions of propargylic electrophiles have not been reported. When considering the use of fluoride anions in propargylic substitution reactions,[20] our attention was drawn to a study by Murahashi and co-workers on the copper(I)-catalyzed amination of propargylic electrophiles.[21a] This strategy for propargylic substitution under copper catalysis has since been expanded to include the use of other nitrogen,[21] carbon,[22] and oxygen[23] nucleophiles [Eq. (2)].


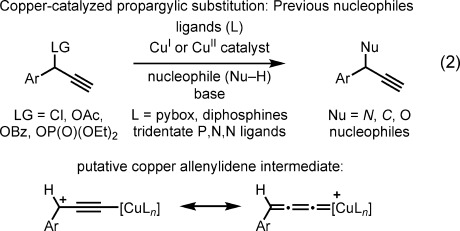


Key features of these transformations include the chemoselectivity of reactions of terminal alkynes, the lack of allene formation, and the enhanced reactivity of 3-aryl substrates relative to their 3-alkyl congeners. Mechanistic investigations indicate nonstereospecific pathways for substitution and have implicated copper allenylidenes[24] as key intermediates.[25] Our objective was to identify a suitable catalyst system that would permit the capture of such putative intermediates by a fluoride anion, while prohibiting the displacement of fluoride from the resulting product. In this endeavor, we focused our attention on 3-alkyl substrates. Herein, we report a catalytic method for the nucleophilic fluorination of propargylic electrophiles [Eq. (3); Ms=methanesulfonyl, Ts=*p*-toluenesulfonyl].[26]


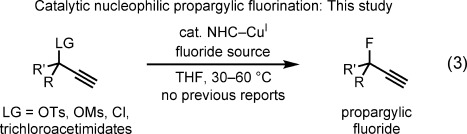


We began our investigations by examining 3-alkyl-substituted propargylic carboxylate esters in conjunction with anionic fluoride sources in the presence of Cu^I^ salts. The use of ligand-free conditions, or catalysts with diphosphine or tridentate pyridine-based ligands, resulted in either low substrate conversion or ester cleavage, presumably as a consequence of the basic nature of the fluoride anion. To circumvent ester cleavage, we extended our substrate survey to include propargylic chloride **1 a-Cl** [Eq. (4); Tf=trifluoromethanesulfonyl]. Full substrate conversion was observed when CuCl (10 mol %) was used, without significant product formation. With Cu^I^–binap or Cu^I^–pybox catalyst systems, shown previously to be suitable for the use of non-halide nucleophiles, complete substrate consumption was possible, but fluoride **2 a** was formed in very low yield along with enyne **3**. Expanding our evaluation of ligand architectures, we found that the copper–N-heterocyclic carbene (NHC) complex [(IPr)CuCl] catalyzed the formation of fluoride **2 a** in 42 % yield, with 33 % starting material remaining. Despite this breakthrough, elimination (15 % yield) was an alarming drawback. Control experiments demonstrated that enyne **3 a** was not formed by *n*Bu_4_NF-mediated elimination from **1 a-Cl** or **2 a** in the absence of [(IPr)CuCl]. Furthermore, when fluoride **2 a** was subjected to the reaction conditions, the formation of enyne **3** was not observed.


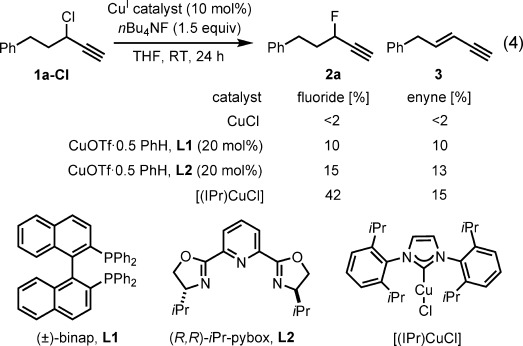


After deducing that basic fluoride sources should be avoided, we turned to acidic fluoride sources, such as Et_3_N⋅3 HF, but chloride **1 a-Cl** was unreactive under such conditions. Since related propargylic substitution reactions are performed in the presence of a base, thus implicating the possible formation of a copper acetylide, we were uncertain as to the viability of propargylic substitution reactions under acidic conditions. However, we found that [(IPr)CuCl] catalyzed the formation of propargylic fluoride **2 a** in good yield from propargylic tosylate **1 a-OTs** in the presence of Et_3_N⋅3 HF as the fluoride source at 30 °C,[27] with the formation of only a trace amount of enyne **3** (Table 1, entry 1). To our knowledge, Cu(NHC) complexes have not previously been demonstrated as catalysts for propargylic substitution reactions of this kind.[28] In the absence of [(IPr)CuCl], no reaction was observed (Table 1, entry 2).[29] The use of ligand-free conditions led to consumption of the substrate without fluorination, and the structure of the NHC ligand had a significant effect on reaction efficiency (Table 1, entries 3–6). By comparison with copper–ligand systems successfully used in propargylic substitution reactions with other nucleophiles (Table 1, entries 7 and 8), [(IPr)CuCl] displayed pronounced efficiency. The catalyst loading could be reduced to 5 mol % with little change in the product yield (Table 1, entry 9). The fluorination was complete in 1 h when the reaction was performed at 60 °C (Table 1, entry 10).

**Table 1:** Effect of the reaction parameters on the catalytic synthesis of secondary propargylic fluorides.^[a]^ 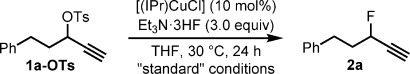

Entry	Variation from “standard” conditions	Conv. [%]^[b]^	Yield [%]^[b]^
1	none	>98	90
2	no [(IPr)CuCl]	<2	<2
3	CuCl instead of [(IPr)CuCl]	>98	<2
4	[(IMes)CuCl] instead of [(IPr)CuCl]	>98	<2
5	[(IPent)CuCl] instead of [(IPr)CuCl]	<2	<2
6	[(SIPr)CuCl] instead of [(IPr)CuCl]	83	72
7	CuOTf⋅0.5 PhH, **L1** (20 mol %)	>98	35
8^[c]^	CuOTf⋅0.5 PhH, **L2** (20 mol %)	>98	55
9	5 mol % instead of 10 mol % [(IPr)CuCl]	98	84
10^[d]^	60 °C instead of 30 °C	>98	90
11	[(IPr)CuF] instead of [(IPr)CuCl]	>98	80
12^[e]^	[(IPr)CuOTs] instead of [(IPr)CuCl]	>98	90
13^[f]^	[(IPr)CuOTf] instead of [(IPr)CuCl]	>98	90 (77)^[g]^
14	mesylate instead of tosylate	96	80
15^[h]^	trichloroacetimidate instead of tosylate	>98	70

[a] All results shown are the average for two experiments performed with 0.1 mmol of the substrate. [b] The conversion and yield were determined by analysis of the reaction mixture by ^1^H NMR spectroscopy with CH_2_Br_2_ as an internal standard. [c] The product was racemic. [d] The reaction was complete within 1 h. [e] The reaction was complete within 5 h. [f] The reaction was complete within 2 h. [g] The yield in parentheses is for a reaction carried out under the standard conditions following the premixing of [(IPr)CuCl] with AgOTf (10 mol %). [h] [(IPr)CuOTf] was used instead of [(IPr)CuCl], and the reaction was performed at 60 °C. 
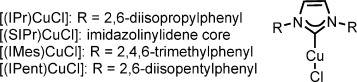

Curious about anion exchange in the (IPr)Cu complex, we treated of a solution of [(IPr)CuCl] in [D_8_]THF with Et_3_N⋅3 HF [Eq. (5)]. Although [(IPr)CuF] was not detected by ^1^H or ^19^F NMR spectroscopy, this complex did catalyze fluoride formation when used as a catalyst (Table 1, entry 11).[30] Conversely, [(IPr)CuOTs] and [(IPr)CuOTf] were shown to be highly active and led to fluorination in 5 and 2 h, respectively (Table 1, entries 12 and 13).[31] The corresponding propargylic mesylate was fluorinated in 80 % yield (Table 1, entry 14). The corresponding trichloroacetimidate was a suitable substrate when [(IPr)CuOTf] was used as the catalyst; in this case, the best results were obtained at 60 °C (Table 1, entry 15). To our knowledge, this acid-activated leaving group has not been deployed in related propargylic substitution reactions.[21–23]


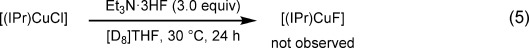


Having identified an appropriate ligand architecture and fluoride source, we elected to move forward with the commercially available complex [(IPr)CuCl] to explore the scope of this fluorination protocol (Scheme 1). Fluoride **2 a** was formed in 87 % yield under the standard reaction conditions. A reaction on a 10 mmol scale provided 1.5 g of the product (90 %). Fluorination in the presence of a furan, an unfunctionalized alkyl chain, an alkene, and a 3-benzyl-substituted substrate proceeded well to give products **2 b**–**e**. We next explored the tolerance of our method towards steric congestion adjacent to the reacting carbon atom. A 3-cyclohexyl substituent was tolerated without modification of the procedure (product **2 f**), and fluorination adjacent to an adamantyl unit occurred in 88 % yield (product **2 g**). The method functioned well with substrates containing a primary chloride, a benzyl ether, an acetal, a methyl ester, and a carbamate (products **2 h**–**l**). The fluorination of an alcohol-containing substrate proceeded in 56 % yield to give **2 m**, and fluorination in the presence of an aldehyde occurred smoothly to give **2 n**. In contrast, many reagents used for dehydrofluorination are incompatible with unprotected aldehydes and alcohols.

**Scheme 1:**
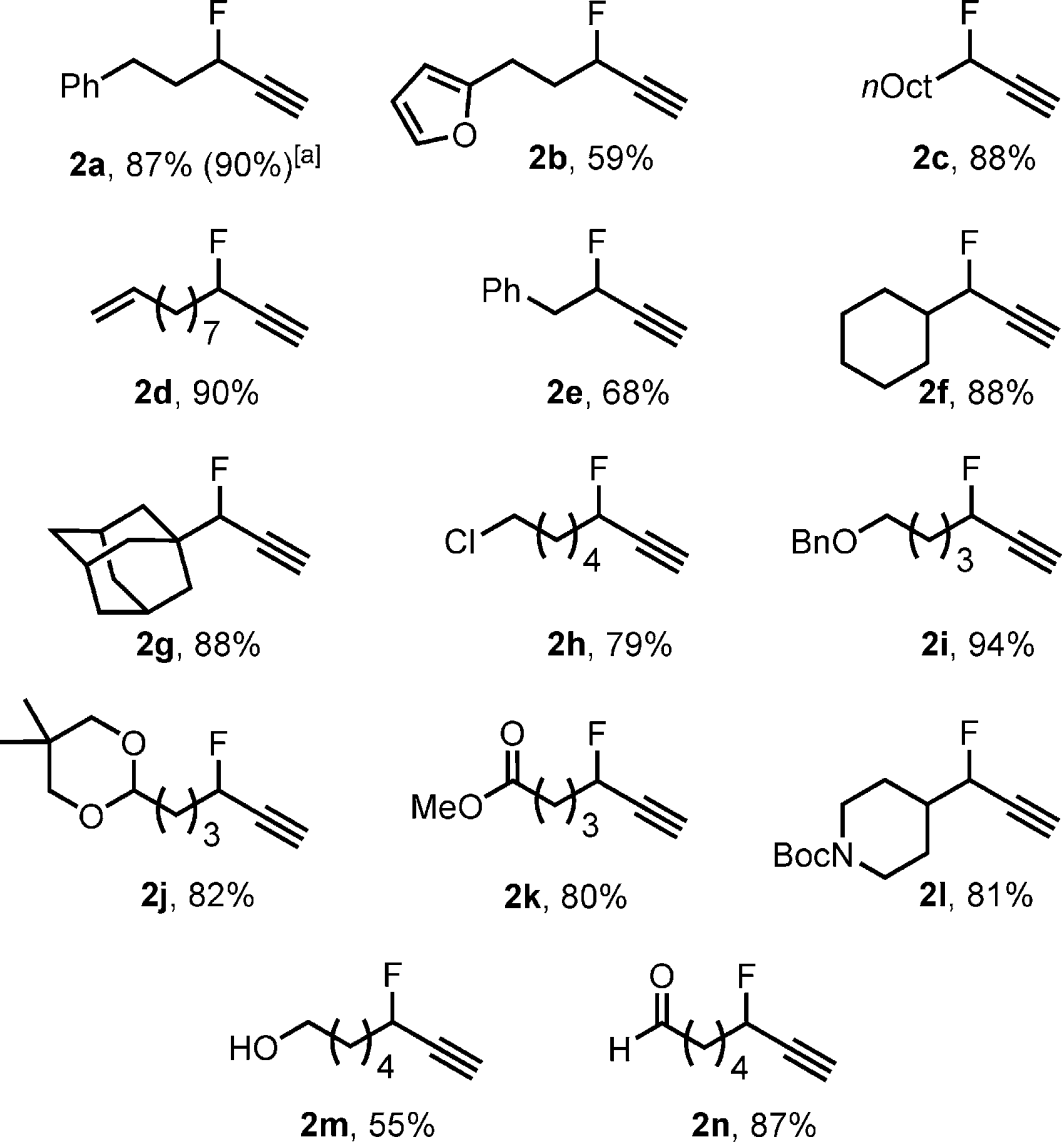
Scope of the catalytic synthesis of secondary propargylic fluorides with respect to the substituents on the electrophile. For reaction conditions, see Table 1. Yields are the average for two experiments with 0.5 mmol of 1. [a] Yield of the purified product of a reaction performed with 10 mmol of 1 a-OTs. Bn=benzyl, Boc=*N*-*tert*-butoxycarbonyl.

The extension of our method to the formation of tertiary propargylic fluorides required an assessment of electrophile stability. To this end, we found that tertiary propargylic trichloroacetimidates could be readily prepared and fluorinated within 2 h with [(IPr)CuOTf] as the catalyst (Scheme 2). The acyclic fluoride **5 a** was formed in 64 % yield, and the method was shown to be compatible with an acetal and a carbamate during preparation of the cyclic tertiary fluorides **5 b** and **5 c**. The synthesis of tertiary fluorides by known protocols is highly challenging, and methods to form tertiary propargylic fluorides often suffer from side reactions, including elimination and 1,2-alkyl shifts. For the synthesis of fluoride **5 c**, protection of the alkyne as a cobalt complex has been required previously to circumvent these problems.[32]

**Scheme 2:**
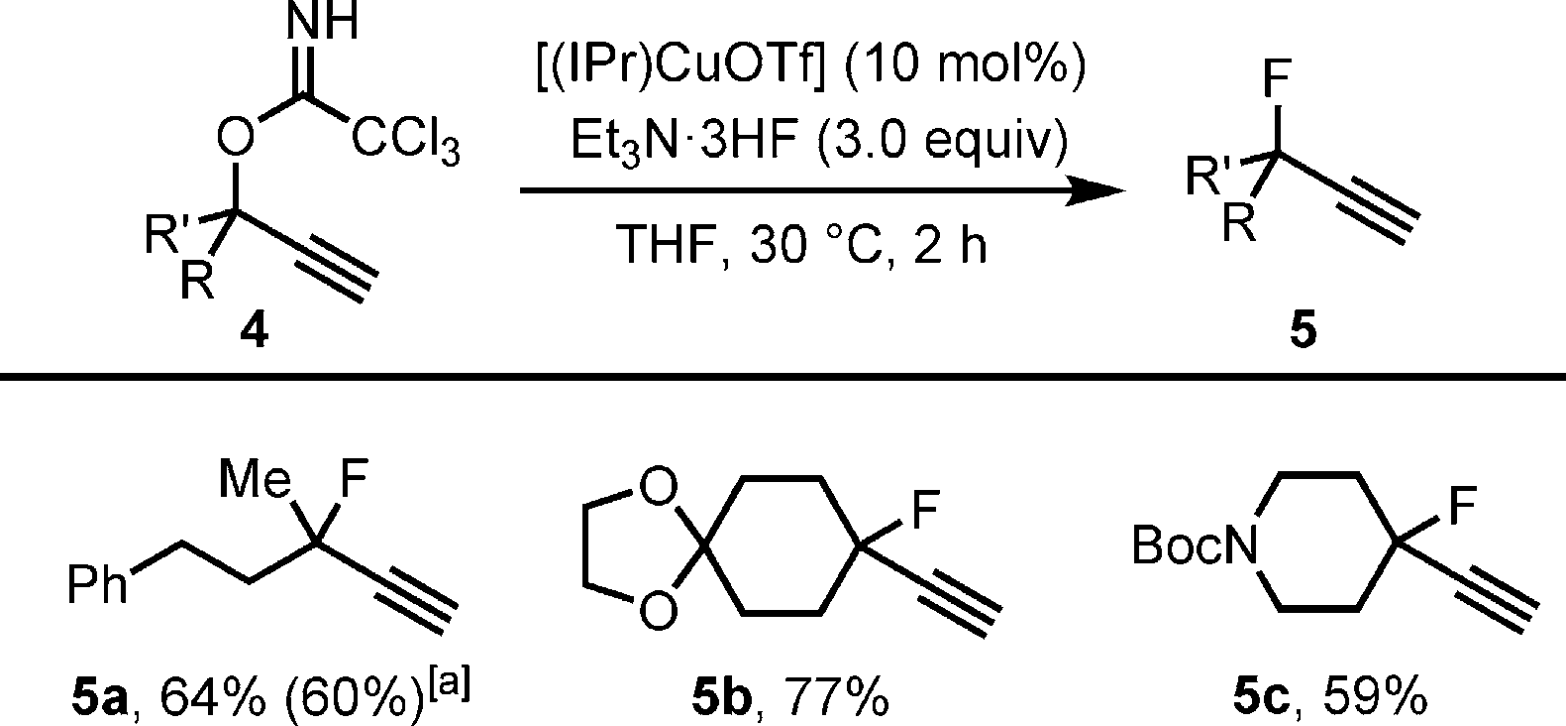
Scope of the catalytic synthesis of tertiary propargylic fluorides with respect to the substituents on the electrophile. Yields are for the purified product and are the average for two experiments with 0.5 mmol of the substrate. [a] The yield in parentheses is for a reaction with [(IPr)CuCl] as the catalyst (a reaction time of 24 h was required).

We performed a series of control experiments to examine some mechanistic features of this process. Under our standard conditions, the enantiomerically enriched tosylate (*R*)**1 a-OTs** (e.r. >99:1) was converted into racemic **2 a** [Eq. (6)].[33] The attempted fluorination of tosylate **6**, bearing an internal alkyne, did not occur, even at elevated temperatures [Eq. (7)]. To investigate the possible formation of a copper acetylide, we prepared a (IPr)Cu–phenylacetylide complex. Under standard conditions, the use of this complex as a catalyst, in place of [(IPr)CuCl], led to fluoride formation in just 1 h [Eq. (8)]. On the basis of these results, our current hypothesis is that the transformation probably occurs via copper acetylides, and achiral cationic species are key intermediates.


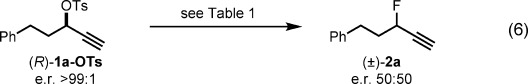



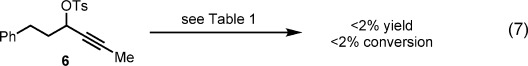



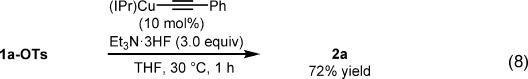


The synthetic importance of terminal alkynes is widely appreciated. We were keen to showcase the synthetic utility of propargylic fluorides and to examine the stability of the fluoride during subsequent modifications (Scheme 3). Sonogashira coupling, one-carbon homologation, and hydrogenation allowed the formation of internal alkyne **7**, allene **8**, and allylic fluoride **9**, respectively. Alkyne hydration with AgSbF_6_ gave methyl ketone **10** in 58 % yield. Conversion of the alkyne group in **2 a** into aromatic moieties was straightforward: Copper-catalyzed cycloaddition with phenyl azide to form triazole **11** proceeded in good yield, and a ruthenium-catalyzed [2+2+2] annulation provided hydroisoindole derivative **12**.

**Scheme 3:**
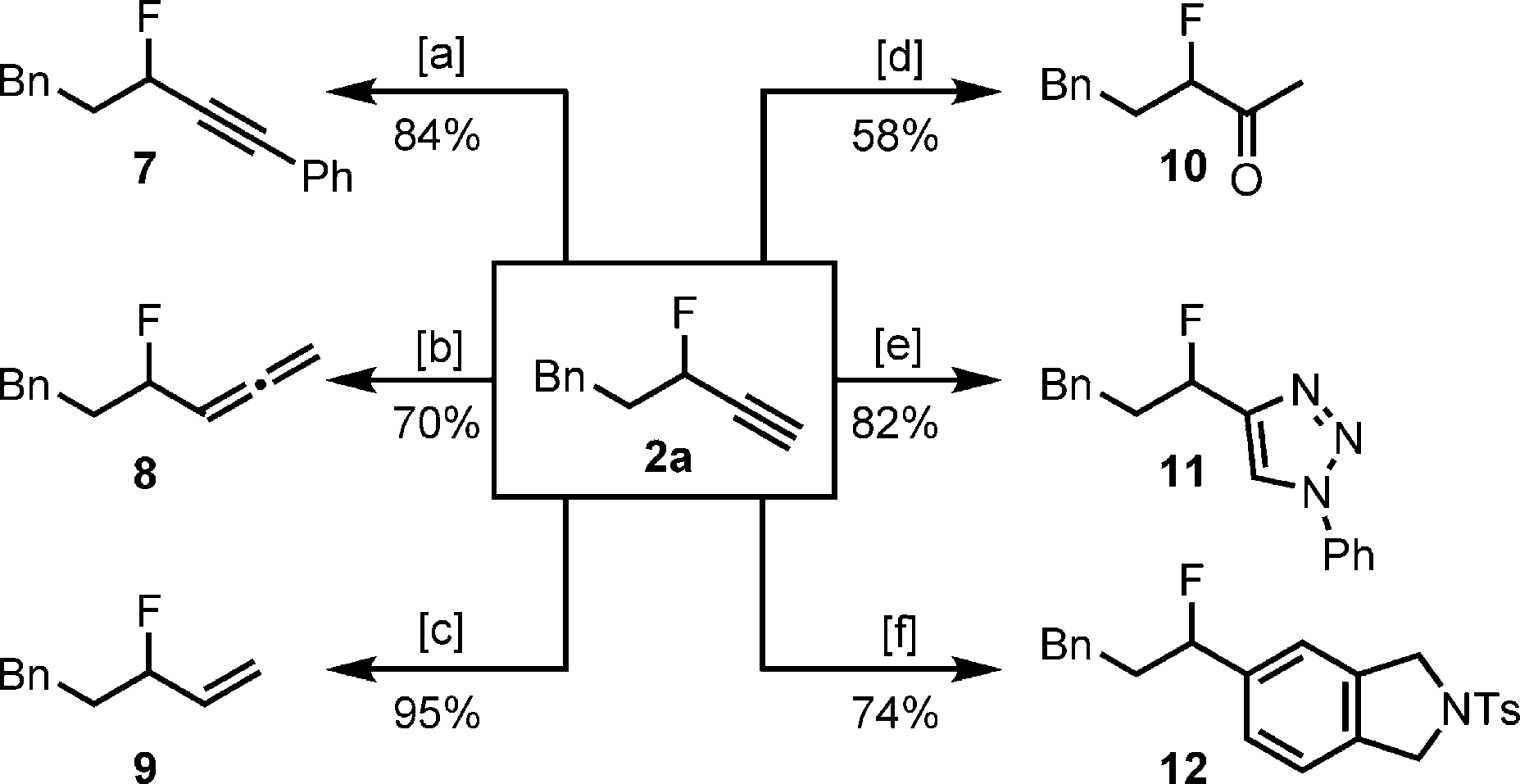
Synthetic transformations of propargylic fluoride 2 a. Yields are for the isolated product of reactions performed with 0.5 mmol of 2 a. Reagents and conditions: [a] [Pd(PPh_3_)_4_] (10 mol %), CuI (10 mol %), PhI, Et_3_N, 60 °C, 8 h; [b] CuI (50 mol %), *N*,*N*-dicyclohexylamine, paraformaldehyde, dioxane, 110 °C, 24 h; [c] H_2_ (1 atm), Lindlar catalyst, quinoline, EtOAc, room temperature, 22 h; [d] AgSbF_6_ (10 mol %), MeOH–H_2_O (10:1), 75 °C, 24 h; [e] CuSO_4_⋅5 H_2_O (10 mol %), sodium ascorbate (20 mol %), PhN_3_, *t*BuOH–H_2_O (1:1), room temperature, 24 h; [f] [Cp*Ru(cod)Cl] (5 mol %), *N*,*N*-bis(propargyl)toluenesulfonamide, DCE, room temperature, 15 min. cod=1,5-cycloctadiene, Cp*=1,2,3,4,5-pentamethylcyclopentadienyl, DCE=1,2-dichloroethane.

In summary, we have described the first catalytic method for nucleophilic propargylic fluorination. The commercially available, air- and moisture-insensitive Cu(NHC) complex [(IPr)CuCl] was used for this transformation, which is suitable for the fluorination of readily available propargylic electrophiles in the presence of a range of functional groups to give secondary and tertiary propargylic fluorides without fluoroallene formation. The transformation is selective for terminal alkynes, and preliminary mechanistic investigations suggest cationic pathways for fluorination. Propargylic fluorides serve as versatile synthetic precursors to a variety of fluorinated building blocks. Efforts to expand the application of Cu(NHC) complexes in propargylic substitution reactions are under way.

## Supporting Information

As a service to our authors and readers, this journal provides supporting information supplied by the authors. Such materials are peer reviewed and may be re-organized for online delivery, but are not copy-edited or typeset. Technical support issues arising from supporting information (other than missing files) should be addressed to the authors.

miscellaneous_information
