# Supplementary material for: Catalytic Nucleophilic Fluorination of Secondary and Tertiary Propargylic Electrophiles with a Copper–N-Heterocyclic Carbene Complex
Source: Angew Chem Int Ed Engl. 2015 Sep 25;54(46):13734–8. doi: 10.1002/anie.201506882 (PMC4648036; doi:10.1002/anie.201506882)
Supplement: Supplementary file 1 — miscellaneous_information [file anie0054-13734-sd1.pdf]

Angewandte Chemie
Eine Zeitschrift der Gesellschaft Deutscher Chemiker

Supporting Information

Catalytic Nucleophilic Fluorination of Secondary and Tertiary Propargylic Electrophiles with a Copper–N-Heterocyclic Carbene Complex

*Li-Jie Cheng and Christopher J. Cordier**

anie_201506882_sm_miscellaneous_information.pdf

Table of Contents

1.	General Information	S–2
2.	General Procedure A: Optimization of Propargylic Fluorination Protocol	S–3
	2.1 Control Experiments	S–3
	2.2 Selected Optimization Experiments	S–5
3.	Synthesis of Catalysts	S–6
4.	Experimental Procedures for Substrate Preparation	S–8
	4.1 General Procedure B: Synthesis of Propargylic Alcohols	S–8
	4.2 General Procedure C: Synthesis of Secondary Propargylic Tosylates	S–15
	4.3 General Procedure D: Synthesis of Tertiary Propargylic Trichloroacetimidates	S–22
5.	General Procedure E: Fluorination of Secondary Propargylic Tosylates (Table 2)	S–24
6.	General Procedure F: Fluorination of Tertiary Propargylic Trichloroacetimidates (Scheme 1)	S–30
7.	Mechanistic Studies (Equations 6–8)	S–32
8.	Synthetic Transformations of Propargylic Fluoride **2a** (Scheme 2)	S–34
9.	HPLC Data for Enantioenriched Substrates	S–38
10.	^{1}H, ^{13}C NMR Spectra of Unknown Compounds	S–44

1. General Information

Practical Considerations. Reactions requiring anhydrous conditions were conducted in oven-dried glassware under at atmosphere of anhydrous N_2. Screens were performed in 4 mL glass vials with a PTFE-lined cap, and all other reactions were performed in round-bottom flasks with rubber septa. Syringes were used to transfer air- and moisture-sensitive reagents. Thin layer chromatography (TLC) was conducted with E. Merck silica gel 60 F254 pre-coated plates (0.25 mm) and visualised initially by UV light (254 nm) or potassium permanganate staining. Purification of compounds was achieved by column chromatography using Merck Flash Silica Gel 60 (230-400 mesh). Organic solutions were concentrated under reduced pressure using a rotary evaporator, using an ice-water bath for volatile compounds.

Materials. Unless otherwise noted, materials obtained from commercial suppliers were used without further purification. THF was distilled from Na/benzophenone under an atmosphere of dry N_2. Et_2O, CH_2Cl_2, CH_3CN, hexane, toluene and were dried on a LC Technology Solutions Inc. SP-1 Solvent Purification System under N_2. (IPr)CuCl, (IMes)CuCl, $Et_3N{\bullet}3HF$, *rac*-BINAP, (*R*)-(*i*-Pr)-Pybox were purchased from Sigma–Aldrich Chemical Company. (SIPr)CuCl,[1] (IAd)CuCl,[2] (IPr*)CuCl,[3] (IPr)CuF,[4] (IPr)Cu(CCPh)[5] were prepared according to literature procedures.

Instrumentation. Nuclear Magnetic Resonance (NMR) spectra were recorded on BRUKER AV (400 MHz) at 298 K. Chemical shifts (δ) are reported in ppm with the residual solvent signal as internal standard (chloroform at 7.26 and 77.00 ppm for ^1H and ^{13}C NMR spectroscopy, respectively). Abbreviations for signal coupling are as follows: s, singlet; d, doublet; t, triplet; q, quartet; quin., quintet; m, multiplet; br., broad. Coupling constants were taken from the spectra directly and are uncorrected and not averaged according to protons determined to exist in the same spin system according to COSY experiments. Signals with the appearance of a given multiplicity are preceded with the term ‘app.’ to denote an apparent splitting pattern when chemically distinct nuclei display very similar coupling constants. ^1H and ^{13}C NMR provided are taken directly using material for which the yield is quoted, without further purification, and are representative of purity. Infrared (IR) spectra were recorded on a PERKIN ELMER Spectrum Two ATR-FT-IR as neat compounds. Absorptions are given in wavenumbers (cm^{-1}). Mass spectra (MS) were obtained on Agilent 5975C spectrometer using electron impact (EI) or Agilent 6220 (for high resolution MS) using electrospray ionization time-of-flight (ESI-TOF). Optical rotation was measured by the Perkin Elmer 341 polarimeter. HPLC analyses were carried out on an Agilent 1260 Infinity Series system, employing Daicel Chiracel columns.

---

[1] Santoro, O.; Collado, A.; Slawin, A. M. Z.; Nolan, S. P.; Cazin, C. S. J. *Chem. Commun*. **2013**, *49*, 10483–10485.
[2] Díez-González, S.; Escudero-Adán, E. C.; Benet-Buchholz, J.; Stevens, E. D.; Slawin, A. M. Z.; Nolan, S. P. *Dalton Trans.* **2010**, *39*, 7595–7606.
[3] Semba, K.; Nakao, Y. *J. Am. Chem. Soc*. **2014**, *136*, 7567–7570.
[4] Wu, S.; Zeng, W.; Wang, Q.; Chen, F.-X. *Org. Biomol. Chem*. **2012**, *10*, 9334–9337.
[5] Goj, L. A.; Blue, E. D.; Munro-Leighton, C.; Gunnoe, T. B.; Petersen, J. L. *Inorg. Chem*. **2005**, *44*, 8647–8649.

2. General Procedure A: Optimization of Propargylic Fluorination Protocol

Propargylic electrophile (0.1 mmol) and catalyst (0.01 mmol) were added in turn to a 4 mL glass vial with a 1 cm stir bar. The vial was capped with a PTFE-lined cap and then it was evacuated and refilled with N_2 three times. Next, solvent (0.5 mL) was added by 1 mL syringe to dissolve the solid, followed by fluoride source (0.3 mmol) added in one portion under N_2 at room temperature. The reaction was heated to 30 °C and stirred at this temperature for 24 h. Then the reaction mixture was filtered through a pad of silica gel (a pipette with about 5 cm silica gel) and washed with ethyl acetate (10 mL). The filtrate was concentrated under reduced pressure. The residue was dissolved in $CDCl_3$ and CH_2Br_2 (0.1 mmol) was added as internal standard for ^1H NMR analysis.

2.1 Control Experiments

Table S1: Control Experiments

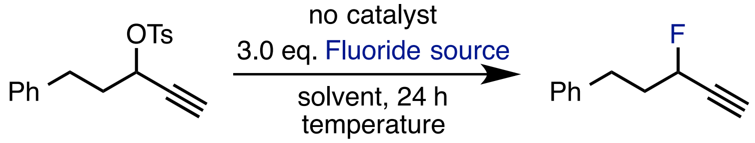

Entry	Fluoride Source	Solvent	Temp (°C)	Conversion (%)	Yield (%)
1	$Et_3N{\bullet}3HF$	THF	30	<2	<2
2	$Et_3N{\bullet}3HF$	THF	60	<2	<2
3	KF	THF	30	<2	<2
4	KF	THF	60	<2	<2
5	CsF	THF	30	<2	<2
6	CsF	THF	60	50	<2
7	$Et_3N{\bullet}3HF$	DMSO	30	<2	<2
8	$Et_3N{\bullet}3HF$	DMSO	100	>98	<2
9	n-$Bu_4NF{\bullet}3H_2O$	DMSO	30	>98	<2
10	KF	DMSO	30	<2	<2
11	KF	DMSO	100	>98	<2
12	CsF	DMSO	30	5	<2
13	CsF	DMSO	100	>98	<2

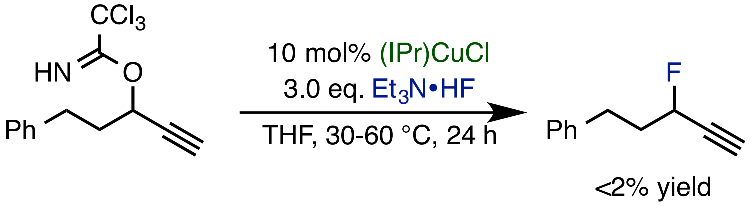

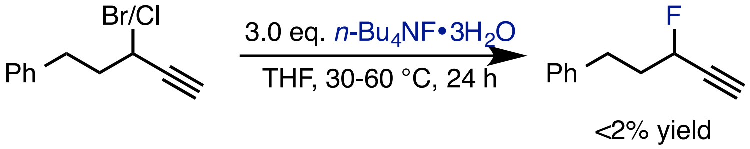

2.2 Selected Optimization of Reactions

Table S2: Catalyst Survey

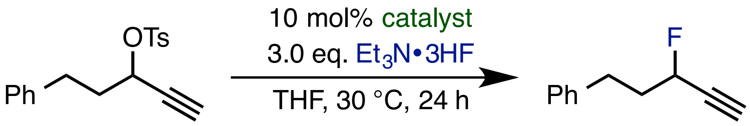

Entry	Catalyst	Conversion (%)	Yield (%)
1	None	0	0
2	CuCl	>98	0
3[a]	CuOTf•1/2C_6H_6/*rac*-BINAP	>98	55
4[a]	CuOTf•1/2C_6H_6/(*R*)-*i*-Pr-Pybox	>98	35
5	(IPr)CuCl	>98	90
6	(SIPr)CuCl	83	72
7	(IMes)CuCl	>98	0
8	(IAd)CuCl	>98	0
9	(IPent)CuCl	0	0
10	(IPr*)CuCl	49	44
11[b]	(IPr)CuOTs	>98	90
12[c]	(IPr)CuOTf	>98	90
13	(IPr)CuF	>98	80

[a]CuOTf•1/2C_6H_6 (10 mol%), *rac*-BINAP (20 mol%) or (*R*)-(*i*-Pr)-Pybox (20 mol%) were premixed in THF at 60 °C for 1 h. [b]The reaction was completed within 5 h. [c]The reaction was completed within 2 h.

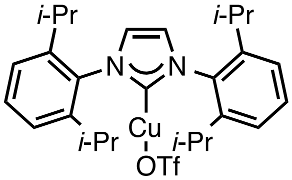

(IPr)CuCl

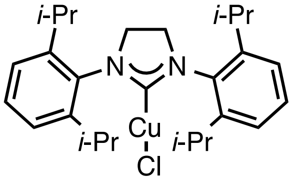

(SIPr)CuCl

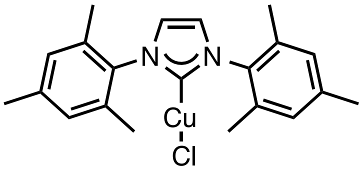

(IMes)CuCl

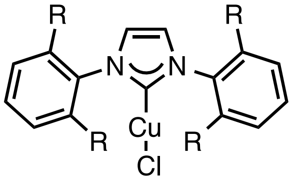

(IPent)CuCl
R = 3-Pent

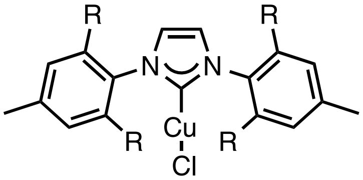

(IPr*)CuCl
R = $CH(Ph)_2$

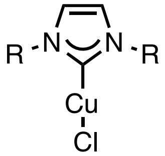

(IAdam)CuCl
R = 1-Adamantyl

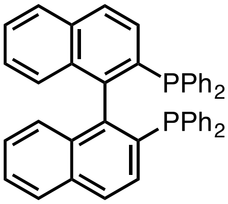

(±)-BINAP

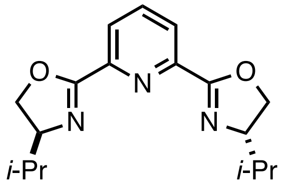

(*R*)-(*i*-Pr)-Pybox

Table S3: Examination of Fluoride Source

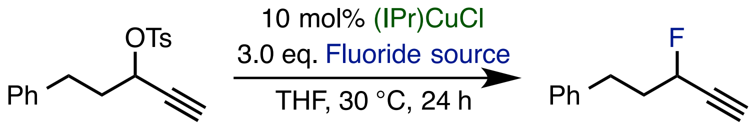

Entry	F^- source	Conversion (%)	Yield (%)
1	Et_3N•3HF	>98	90
2	AgF	>98	<2
3	KF	>98	<2
4	CsF	>98	<2
5	$Bu_4NSiPh_3F_2$	>98	<2
6	TBAF•$3H_2O$	>98	56
7	TBAF•4^{t}BuOH	>98	49
8	9HF•Py	<2	<2

Table S4: Study of Leaving Groups

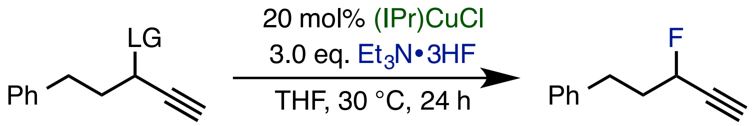

Entry	LG	Conversion (%)	Yield (%)
1	-OTs	>98	90
2	-OMs	96	80
3	-Cl	<2	<2
4	-Br	<2	<2
5	-OAc	<2	<2
6	-OCO_2Me	<2	<2

Table S5: Solvent Evaluation

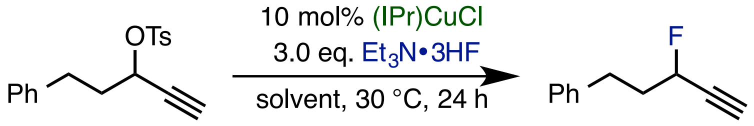

Entry	Solvent	Conversion (%)	Yield (%)
1	THF	>98	90
2	CH_2Cl_2	84	58
3	Toluene	30	28
4	CH_3CN	85	74
5	1,4-dioxane	84	46
6	Hexane	>98	75
7	Et_2O	98	84
8	EtOAc	98	86

3. Synthesis of Catalysts

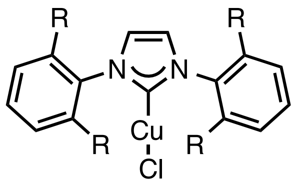

R = 3-Pent

(IPent)CuCl: IPent•HCl[6] (179 mg, 0.3 mmol), NaO*t*-Bu (32 mg, 0.3 mmol) and CuCl (33 mg, 0.3 mmol) were added quickly in turn to a Schlenk flask with a 1 cm stir bar under air. The flask was evacuated and refilled with N_2. Then THF (3 mL) was added by syringe under N_2 and the resulting mixture was stirred at room temperature for 3 h. Then the mixture was filtered through a pad of Celite (a pipette with about 5 cm Celite) and washed with dry CH_2Cl_2. The filtrate was removed under the reduced pressure. The resulting solid was then dissolved into CH_2Cl_2 (3 mL) and triturated with hexane (20 mL). The suspension was filtered through a pad of Celite (a pipette with about 5 cm Celite) and filtrate was concentrated to afford the title product as an off-white solid (60 mg, 30%).

^{1}H NMR (400 MHz, $CDCl_3$) δ 7.47 (t, *J* = 7.7 Hz, 2H), 7.21 (d, *J* = 7.8 Hz, 4H), 7.01 (s, 2H), 2.14 (app. quin., *J* = 7.2 Hz, 4H), 1.79 – 1.45 (m, 16H), 0.91 (t, *J* = 7.3 Hz, 12H), 0.78 (t, *J* = 7.3 Hz, 12H);

^{13}C NMR (101 MHz, $CDCl_3$) δ 180.2, 143.2, 137.2, 130.0, 124.6, 123.6, 42.8, 29.3, 28.6, 13.0, 12.5.

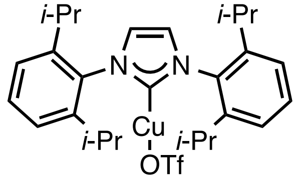

(IPr)CuOTf: (IPr)CuCl (244 mg, 0.5 mmol) and AgOTf (128 mg, 0.5 mmol) were added in turn to a 4 mL glass vial with a 1 cm stir bar. The vial was capped with a PTFE-lined cap and then it was evacuated and refilled with N_2 three times. Next, THF (2 mL) was added to the vial by syringe under N_2. The mixture was allowed to stir at room temperature for 3 hours. Then the resulting white solids were filtered off a pad of Celite (a pipette with about 5 cm Celite) and washed with dry CH_2Cl_2. The filtrate was concentrated under reduced pressure to afford the tittle compound as an off-white solid (300 mg, >98% yield), which was used without further purification.

^{1}H NMR (400 MHz, $CDCl_3$) δ 7.52 (t, *J* = 7.8 Hz, 2H), 7.32 (d, *J* = 7.7 Hz, 4H), 7.21 (s, 2H), 2.50 (app. quin., *J* = 6.9 Hz, 4H), 1.26 (d, *J* = 6.8 Hz, 12H), 1.23 (d, *J* = 6.8 Hz, 12H);

^{13}C NMR (101 MHz, $CDCl_3$) δ 177.7, 145.4, 133.9, 130.7, 124.3, 123.9, 123.7, 120.8, 119.3 (q, *J* = 316.1 Hz), 28.7, 24.6, 23.9.

---

[6] Meiries, S.; LeDuc, G.; Chartoire, A.; Collado, A.; Speck, K.; Arachchige, K. S. A.; Slawin, A. M. Z.; Nolan, S. P. *Chem. Eur. J.* **2013**, *19*, 17358–17368.

^{19}F {^{1}H} NMR (377 MHz, $CDCl_3$) δ –77.9.

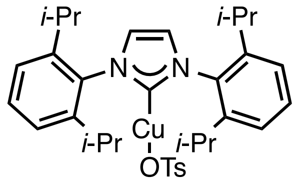

(IPr)CuOTs: (IPr)CuCl (244 mg, 0.5 mmol) and AgOTs (139 mg, 0.5 mmol) were added in turn to a 4 mL glass vial with a 1 cm stir bar. The vial was capped with a PTFE-lined cap and then it was evacuated and refilled with N_2 three times. Next, THF (2 mL) was added to the vial by syringe under N_2. The mixture was allowed to stir at room temperature for 3 hours. Then the resulting white solids were filtered off a pad of Celite (a pipette with about 5 cm Celite) and washed with dry CH_2Cl_2. The filtrate was concentrated under reduced pressure to afford the tittle compound as an off-white solid (300 mg, 96%), which was used without further purification.

4. Experimental Procedures for Substrate Preparation

4.1 General Procedure B: Synthesis of Propargylic Alcohols[7]

A round-bottom flask with a stir bar was dried in the oven and cooled under N_2. Ethynylmagnesium bromide in THF (0.5 M in THF, 1.2 eq.) was transferred to the flask via syringe. Then the reaction was cooled to 0 °C. Aldehyde (1.0 eq.) was dissolved in THF (0.5 M) and added dropwise via syringe to the above solution at 0 °C. After 30 min, the cooling bath was removed to reach room temperature. The mixture was stirred for a further 2 hours. Saturated aqueous ammonium chloride was then added and the mixture was extracted with Et_2O three times. The combined organic layers were washed with a saturated aqueous sodium chloride solution, dried over magnesium sulphate, filtered and the solvents were removed under reduced pressure. The residue was purified by silica gel chromatography (Hexane/ethyl acetate as eluent) to give the corresponding propargylic alcohol. Yields have not been optimized.

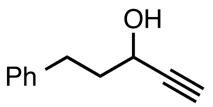

5-Phenyl-1-pentyn-3-ol. Prepared according to general procedure B using ethynylmagnesium bromide (0.5 M in THF, 48 mL, 24 mmol), 3-phenyl-propionaldehyde (13.4 g, 20 mmol). The crude material was purified by silica gel chromatography (hexanes/ethyl acetate = 4:1) to afford the title compound as a light yellow oil (3.0 g, 94% yield). All spectral data are in accordance with the literature.[8]

^{1}H NMR (400 MHz, $CDCl_3$) δ 7.32 – 7.27 (m, 2 H), 7.23 – 7.18 (m, 3 H), 4.38 (td, *J* = 6.6, 2.0 Hz, 1 H), 2.81 (t, *J* = 7.8 Hz, 2 H), 2.52 (d, *J* = 2.1 Hz, 1H), 2.11 – 2.02 (m, 3 H);

^{13}C NMR (101 MHz, $CDCl_3$) δ 141.0, 128.5, 128.4, 126.0, 84.6, 73.3, 61.5, 39.0, 31.2.

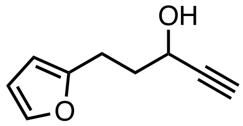

5-(2-Furyl)pent-1-yn-3-ol. Prepared according to general procedure B utilizing ethynylmagnesium bromide (0.5 M in THF, 17 mL, 8.5 mmol), 3-furan-2-yl-propionaldehyde (875 mg, 7.1 mmol). The crude material was purified by silica gel chromatography (hexanes/ethyl acetate = 4:1) to afford the title compound as a light yellow oil (1.0 g, >98% yield). All spectral data are in accordance with the literature.[9]

^{1}H NMR (400 MHz, $CDCl_3$) δ 7.31 (d, *J* = 1.9 Hz, 1H), 6.28 (dd, *J* = 3.2, 2.0 Hz, 1H), 6.02 – 6.03 (m, 1H), 4.54 – 4.32 (m, 1H), 2.91 – 2.76 (m, 2H), 2.50 (d, *J* = 2.0 Hz, 1H), 2.09 – 2.03 (m, 3H);

^{13}C NMR (101 MHz, $CDCl_3$) δ 154.8, 141.1, 110.2, 105.4, 84.4, 73.4, 61.5, 35.8, 23.6.

[7] Allegretti, P. A.; Ferreira, E. M. *Org. Lett.* **2011**, *13*, 5924–5927.
[8] Pacheco, M. C.; Gouverneur, V. *Org. Lett.* **2005**, *7*, 1267–1270.
[9] Bull, J. R.; Gordon, R.; Hunter, R. *J. Chem. Soc., Perkin Trans. 1*, **2000**, 3129–3139.

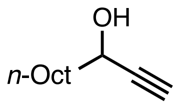

1-Undecyn-3-ol. Prepared according to general procedure B utilizing ethynylmagnesium bromide (0.5 M in THF, 36.0 mL, 18.0 mmol), nonanal (2.13 g, 15 mmol). The crude material was purified by silica gel chromatography (hexanes/ethyl acetate = 8:1) to afford the title compound as a colorless oil (1.95 g, 78% yield). All spectral data are in accordance with the literature.[10]

^{1}H NMR (400 MHz, $CDCl_3$) δ 4.36 (app. qd, *J* = 6.6, 2.1 Hz, 1H), 2.45 (d, *J* = 2.3 Hz, 1H), 2.08 (d, *J* = 5.3 Hz, 1H), 1.75 – 1.63 (m, 2H), 1.49 – 1.39 (m, 2H), 1.34 – 1.21 (m, 10H), 0.90 – 0.83 (m, 3H);

^{13}C NMR (101 MHz, $CDCl_3$) δ 85.1, 72.8, 62.3, 37.7, 31.9, 29.5, 29.2 (2C), 25.0, 22.7, 14.1.

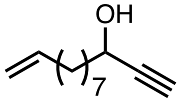

Dodec-11-en-1-yn-3-ol. Prepared according to general procedure B utilizing ethynylmagnesium bromide (0.5 M in THF, 28.0 mL, 14.0 mmol), dec-9-enal (1.84 g, 12 mmol). The crude material was purified by silica gel chromatography (hexanes/ethyl acetate = 5:1) to afford the title compound as a colorless oil (1.78 g, 83% yield).

^{1}H NMR (400 MHz, $CDCl_3$) δ 5.80 (ddt, *J* = 16.9, 10.2, 6.7 Hz, 1H), 5.06 – 4.85 (m, 2H), 4.35 (td, *J* = 6.6, 2.1 Hz, 1H), 2.45 (d, *J* = 2.3 Hz, 1H), 2.03 (app. q, *J* = 7.1 Hz, 2H), 1.75 – 1.62 (m, 2H), 1.50 – 1.23 (m, 11H);

^{13}C NMR (101 MHz, $CDCl_3$) δ 139.1, 114.1, 85.0, 72.8, 62.2, 37.6, 33.7, 29.3, 29.1, 29.0, 28.8, 25.0;

IR (neat) 3309, 2927, 2856, 2115, 1640, 1465, 910, 654 cm^{-1};

HRMS (EI) Calcd. for $C_{12}H_{20}O$ ($[M]^+$): 180.1514; Found: 180.1506.

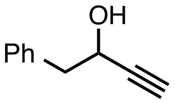

4-Phenylbut-1-yn-3-ol. Prepared according to general procedure B utilizing ethynylmagnesium bromide (0.5 M in THF, 36.0 mL, 18.0 mmol), phenylacetaldehyde (1.8 g, 15 mmol). The crude material was purified by silica gel chromatography (hexanes/ethyl acetate = 5:1) to afford the title compound as a yellow oil (1.5 g, 70% yield). All spectral data are in accordance with the literature.[11]

^{1}H NMR (400 MHz, $CDCl_3$) δ 7.38 – 7.30 (m, 5H), 4.61 (app. qd, *J* = 6.1, 2.1 Hz, 1H), 3.08 (dd, *J* = 13.4, 6.5. 1H), 3.03 (dd, *J* = 13.6, 6.5 Hz, 1H), 2.52 (d, *J* = 2.1 Hz, 1H), 2.11 (d, *J* = 5.5 Hz, 1H);

^{13}C NMR (101 MHz, $CDCl_3$) δ 136.2, 129.7, 128.4, 127.0, 84.1, 73.8, 62.9, 43.8.

[10] Santos, D.; Ariza, X.; Garcia, J.; Lloyd-Williams, P.; Martínez-Laporta, A.; Sánchez, C. *J. Org. Chem.* **2013**, 78, 1519–1524.
[11] Kong, W.; Guo, B.; Fu, C.; Ma, S. *Eur. J. Org. Chem.* **2011**, 2278–2285.

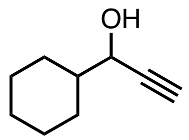

3-Cyclohexylpropyl-1-yn-3-ol. Prepared according to general procedure B utilizing ethynylmagnesium bromide (0.5 M in THF, 36.0 mL, 18.0 mmol), cyclohexanecarbaldehyde (1.68 g, 15 mmol). The crude material was purified by silica gel chromatography (hexanes/ethyl acetate = 7:1) to afford the title compound as a colorless oil (2.0 g, 99% yield). All spectral data are in accordance with the literature.[12]

^{1}H NMR (400 MHz, $CDCl_3$) δ 4.15 (app. td, *J* = 5.8, 2.1 Hz, 1H), 2.46 (d, *J* = 2.0 Hz, 1H), 1.94 (d, *J* = 5.8 Hz, 1H), 1.89 – 1.73 (m, 4H), 1.72 – 1.63 (m, 1H), 1.61 – 1.50 (m, 1H), 1.32 – 1.03 (m, 5H);

^{13}C NMR (101 MHz, $CDCl_3$) δ 84.0, 73.6, 67.0, 43.9, 28.4, 28.0, 26.3, 26.3, 25.8.

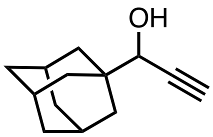

1-(1-Adamantyl)prop-2-yn-1-ol. Prepared according to general procedure B utilizing ethynylmagnesium bromide (0.5 M in THF, 36.0 mL, 18.0 mmol), 1-adamantanecarboxaldehyde (2.46 g, 15.0 mmol). The crude material was purified by silica gel chromatography (hexanes/ethyl acetate = 5:1) to afford the title compound as a white solid (2.32 g, 90% yield). All spectral data are in accordance with the literature.[13]

^{1}H NMR (400 MHz, $CDCl_3$) δ 3.87 (br. d, *J* = 4.5 Hz, 1H), 2.46 (d, *J* = 1.9 Hz, 1H), 2.01 (t, *J* = 3.4 Hz, 3H), 1.82 (d, *J* = 5.6 Hz, 1H), 1.74 – 1.56 (m, 12H);

^{13}C NMR (101 MHz, $CDCl_3$) δ 82.8, 74.2, 71.4, 37.5, 37.0, 37.1, 28.2.

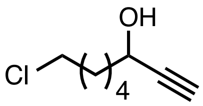

8-Chlorooct-1-yn-3-ol. Prepared according to general procedure B utilizing ethynylmagnesium bromide (0.5 M in THF, 28.4 mL, 14.2 mmol), 6-chlorohexanal (1.6 g, 11.9 mmol). The crude material was purified by silica gel chromatography (hexanes/ethyl acetate = 6:1) to afford the title compound as a yellow oil (3.0 g, 94% yield).

^{1}H NMR (400 MHz, $CDCl_3$) δ 4.38 (qd, *J* = 6.5, 2.2 Hz, 1H), 3.54 (t, *J* = 6.7 Hz, 2H), 2.47 (d, *J* = 2.0 Hz, 1H), 1.95 (d, *J* = 5.0 Hz, 1H), 1.85 – 1.68 (m, 4H), 1.53 – 1.43 (m, 4H);

^{13}C NMR (101 MHz, $CDCl_3$) δ 84.8, 73.1, 62.1, 44.9, 37.4, 32.5, 26.5, 24.3;

IR (neat) 3295, 2941, 2863, 2114, 1711, 1462, 1020, 649 cm^{-1}.

[12] Ye, L.; He, W.; Zhang, L. *J. Am. Chem. Soc.* **2010**, *132*, 8550–8551.
[13] Ziffle, V. E.; Cheng, P.; Clive, D. L. J. *J. Org. Chem*. **2010**, *75*, 8024–8038.

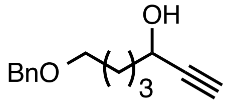

7-(Benzyloxy)hept-1-yn-3-ol. Prepared according to general procedure B utilizing ethynylmagnesium bromide (0.5 M in THF, 36 mL, 18.0 mmol), 5-benzyloxy-1-pentanal (2.9 g, 15.0 mmol). The crude material was purified by silica gel chromatography (hexanes/ethyl acetate = 4:1) to afford the title compound as a yellow oil (3.0 g, 94% yield). All spectral data are in accordance with the literature.[14]

^{1}H NMR (400 MHz, $CDCl_3$) δ 7.39 – 7.35 (m, 4H), 7.33 – 7.28 (m, 1H), 4.53 (s, 2H), 4.39 (app. qd, J = 6.4, 2.1 Hz, 1H), 3.52 (t, J = 6.4 Hz, 2H), 2.48 (s, 1H), 2.04 (d, J = 5.4 Hz, 1H), 1.80 – 1.55 (m, 6H);

^{13}C NMR (101 MHz, $CDCl_3$) δ 138.5, 128.4, 127.7, 127.6, 127.6, 84.9, 72.9, 70.1, 62.2, 37.4, 29.3, 21.8.

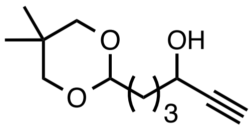

6-(5,5-Dimethyl-1,3-dioxan-2-yl)hex-1-yn-3-ol. Prepared according to general procedure B utilizing ethynylmagnesium bromide (0.5 M in THF, 16.6 mL, 8.3 mmol), 4-(5,5-dimethyl-1,3-dioxan-2-yl)butanal (1.29 g, 6.9 mmol). The crude material was purified by silica gel chromatography (hexanes/ethyl acetate = 3:1) to afford the title compound as a yellow oil (0.9 g, 61% yield).

^{1}H NMR (400 MHz, $CDCl_3$) δ 4.38 (t, J = 4.8 Hz, 1H), 4.30 (app. qd, J = 6.5, 2.3 Hz, 1H), 3.55 (d, J = 11.3 Hz, 2H), 3.37 (d, J = 10.9 Hz, 2H), 2.77 (d, J = 5.4 Hz, 1H), 2.41 (d, J = 2.0 Hz, 1H), 1.74 – 1.48 (m, 6H), 1.13 (s, 3H), 0.67 (s, 3H);

^{13}C NMR (101 MHz, $CDCl_3$) δ 101.9, 85.0, 77.3, 72.7, 61.8, 61.8, 37.3, 34.1, 30.0, 22.9, 21.7, 19.5;

IR (neat) 3434, 3296, 2955, 2869, 2112, 1737, 1471, 1396, 1135, 1105, 1041, 1014, 972 cm^{-1};

HRMS (EI) Calcd. for $C_{12}H_{20}O_3$ ($[M]^+$): 212.1412; Found: 212.1419.

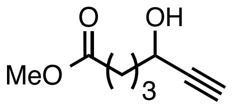

Methyl 5-hydroxy-hept-6-ynoate. A 100 mL round-bottom flask with a 2 cm stir bar was dried in the oven and cooled under N_2. 4-methoxycarbonyl-butanal (1.60 g, 14.0 mmol) and THF (20 mL) were added in turn via syringe to the flask. Then the reaction was cooled to 0 °C. Ethynylmagnesium bromide in THF (0.5 M in THF, 34.0 mL, 17.0 mmol) was added dropwise via syringe for 15 min to the above solution at 0 °C. After 30 min, the cooling bath was removed to reach room temperature. The mixture was stirred for a further 2 hours. Saturated aqueous ammonium chloride (20 mL) was then added and the mixture was extracted with Et_2O (20 mL) three

[14] Yadav, J. S.; Raju, A.; Ravindar, K.; Reddy, B. V. S.; Khazim Al Ghamdi, A. A. *Synthesis* **2012**, *44*, 585–590.

times. The combined organic layers were washed with brine (15 mL), dried over magnesium sulphate, filtered and the solvents were removed under reduced pressure. The residue was purified by Silica gel chromatography (hexanes/ethyl acetate = 2:1) to afford the title compound as a yellow oil (0.65 g, 34% yield). All spectral data are in accordance with the literature.[15]

^{1}H NMR (400 MHz, $CDCl_3$) δ 4.37 (app. qd, *J* = 6.0, 2.1 Hz, 1H), 3.65 (s, 2H), 2.48 – 2.44 (m, 2H), 2.36 (t, *J* = 7.1 Hz, 2H), 1.84 – 1.70 (m, 4H);

^{13}C NMR (101 MHz, $CDCl_3$) δ 173.9, 84.5, 73.0, 61.7, 51.6, 36.7, 33.4, 20.3.

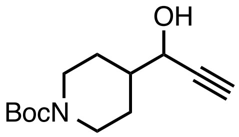

***tert*-Butyl 4-(1-hydroxyprop-2-yn-1-yl)piperidine-1-carboxylate.** Prepared according to general procedure B utilizing ethynylmagnesium (0.5 M in THF, 11.2 mL, 5.6 mmol), 4-formyl-piperidine-1-carboxylic acid tert-butyl ester (1.0 g, 4.7 mmol). The crude material was purified by silica gel chromatography (hexanes/ethyl acetate = 2:1) to afford the title compound as a light yellow oil (1.0 g, 89% yield).

^{1}H NMR (400 MHz, $CDCl_3$) δ 4.21 – 4.07 (m, 3H), 2.73 – 2.52 (m, 3H), 2.46 (d, *J* = 2.0 Hz, 1H), 1.82 – 1.77 (m, 2H), 1.74 – 1.65 (m, 1H), 1.43 (s, 9H), 1.36 – 1.19 (m, 2H);

^{13}C NMR (101 MHz, $CDCl_3$) δ 154.8, 83.3, 79.4, 74.0, 65.9, 43.5 (2C), 42.3, 28.4, 27.6, 27.1;

IR (neat) 3406, 3307, 2977, 2931, 2247, 1673, 1429, 1170, 733 cm^{-1}.

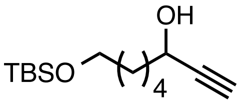

8-(*tert*-Butyldimethylsilyloxy)oct-1-yn-3-ol. Prepared according to general procedure B utilizing ethynylmagnesium bromide (0.5 M in THF, 24.0 mL, 12.0 mmol), 6-(tert-butyl-dimethyl-silanyloxy)-hexanal (2.3 g, 10.0 mmol). The crude material was purified by silica gel chromatography (hexanes/ethyl acetate = 10:1) to afford the title compound as a colorless oil (2.2 g, 85% yield). All spectral data are in accordance with the literature.[16]

^{1}H NMR (400 MHz, $CDCl_3$) δ 4.36 (app. qd, *J* = 6.5, 2.1 Hz, 1H), 3.60 (t, *J* = 6.4 Hz, 2H), 2.45 (d, *J* = 2.3 Hz, 1H), 2.09 (d, *J* = 5.5 Hz, 1H), 1.77 – 1.66 (m, 2H), 1.56 – 1.43 (m, 4H), 1.40 – 1.32 (m, 2H), 0.88 (s, 9H), 0.03 (s, 6H);

^{13}C NMR (101 MHz, $CDCl_3$) δ 85.0, 72.8, 63.1, 62.2, 37.6, 32.7, 26.0, 25.4, 24.8, 18.3, –5.3.

[15] Götz, K.; Liermann, J. C.; Thines, E.; Ankeb, H.; Opatz, T. *Org. Biomol. Chem.* **2010**, *8,* 2123–2130.
[16] Katsuhira, T.; Harada, T.; Oku, A. *J. Org. Chem.* **1994**, *59*, 4010–4014.

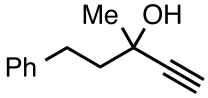

3-Methyl-5-phenylpent-1-yne-3-ol. Prepared according to general procedure B utilizing ethynylmagnesium bromide (0.5 M in THF, 48.0 mL, 24.0 mmol), 4-phenyl-butan-2-one (2.92 g, 20.0 mmol). The crude material was purified by silica gel chromatography (hexanes/ethyl acetate = 5:1) to afford the title compound as a colorless oil (2.88 g, 82% yield). All spectral data are in accordance with the literature.[17]

^{1}H NMR (400 MHz, $CDCl_3$) δ 7.38 – 7.32 (m, 2H), 7.30 – 7.22 (m, 3H), 2.99 – 2.85 (m, 2H), 2.56 (s, 1H), 2.35 (s, 1H), 2.12 – 1.97 (m, 2H), 1.61 (s, 3H);

^{13}C NMR (101 MHz, $CDCl_3$) δ 141.7, 128.4 (2C), 125.9, 87.3, 71.8, 67.9, 45.1, 31.0, 29.9.

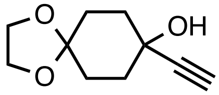

8-Ethynyl-1,4-dioxaspiro[4.5]decan-8-ol. Prepared according to general procedure B utilizing ethynylmagnesium bromide (0.5 M in THF, 36.0 mL, 18.0 mmol), 1,4-cyclohexanedione monoacetal (2.34 g, 15.0 mmol). The crude material was purified by silica gel chromatography (hexanes/ethyl acetate = 2:1) to afford the title compound as a yellow oil (1.95 g, 71% yield). All spectral data are in accordance with the literature.[18]

^{1}H NMR (400 MHz, $CDCl_3$) δ 3.91 (s, 4H), 2.50 (s, 1H), 2.46 (s, 1H), 1.98 – 1.85 (m, 4H), 1.78 – 1.74 (m, 4H);

^{13}C NMR (101 MHz, $CDCl_3$) δ 107.9, 86.9, 72.0, 67.0, 64.2, 64.2, 36.9, 31.2.

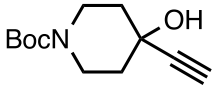

***tert*-Butyl 4-ethynyl-4-hydroxypiperidine-1-carboxylate.** Prepared according to general procedure B utilizing ethynylmagnesium bromide (0.5 M in THF, 36.0 mL, 18.0 mmol), *tert*-butyl 4-oxo-piperidine-1-carboxylate (3.0 g, 15.0 mmol). The crude material was purified by silica gel chromatography (hexanes/ethyl acetate = 3:1) to afford the title compound as a white solid (2.9 g, 82% yield). All spectral data are in accordance with the literature.[19]

^{1}H NMR (400 MHz, $CDCl_3$) δ 3.71 (br. s, 2H), 3.26 – 3.19(m, 2H), 2.48 (s, 1H), 1.92 – 1.76 (m, 2H), 1.70 – 1.64 (m, 2H), 1.41 (s, 9H);

^{13}C NMR (101 MHz, $CDCl_3$) δ 154.6, 86.2, 79.7, 72.9, 66.3, 40.6, 38.7, 28.3.

[17] Egi, M.; Yamaguchi, Y.; Fujiwara, N.; Akai, S. *Org. Lett.* **2008**, *10*, 1867–1870.
[18] Ni, Z. Laurent, G.; Alphonse, T. *Chem. Eur. J.* **2014**, *20*, 11703–11706.
[19] Xi, N. Yin, L. Li, X. Yu, N. Wu, Y. Patent: WO2013/177092 A1, **2013**

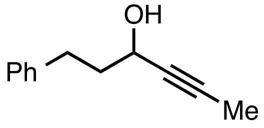

1-Phenylhex-4-yn-3-ol. Prepared according to general procedure B utilizing 1-propynylmagnesium bromide (0.5 M in THF, 24.0 mL, 12.0 mmol), 3-phenyl-propionaldehyde (1.34 g, 10.0 mmol). The crude material was purified by silica gel chromatography (hexanes/ethyl acetate = 5:1) to afford the title compound as a light yellow oil (1.5 g, 86% yield). All spectral data are in accordance with the literature.[20]

^{1}H NMR (400 MHz, $CDCl_3$) δ 7.33 – 7.27 (m, 2H), 7.25 – 7.17 (m, 3H), 4.35 (app. tq, *J* = 6.5, 2.1 Hz, 1H), 2.80 (t, *J* = 7.8 Hz, 2H), 2.09 – 1.94 (m, 3H), 1.87 (d, *J* = 2.0 Hz, 3H);

^{13}C NMR (101 MHz, $CDCl_3$) δ 141.4, 128.4, 128.3, 125.8, 81.3, 80.1, 61.9, 39.5, 31.4, 3.5.

[20] Pacheco, M. C.; Gouverneur, V. *Org. Lett.* **2005**, *7*, 1267–1270.

4.2. General Procedure C: Synthesis of Secondary Propargylic Tosylates[21]

In air, TsCl (1.1 eq.) and propargylic alcohol (1.0 eq) were added in turn to a round-bottom flask with a 2.0 cm stir bar. Et_2O (0.2 M) was added to dissolve the solid and then the reaction mixture was cooled at –10 °C. Next, freshly fine powdered KOH (7.4 eq) was added portion wise by spatula while keeping temperature below –10 °C. The mixture was stirred at –5 °C for 1 h before poured into ice water (the same volume of Et_2O). After vigorous shaking, the layers were separated and the aqueous layer was extracted twice with Et_2O. The combined organic layers were washed with brine and dried over $MgSO_4$. After the solvent was removed on a rotary evaporator, the residue was purified by silica gel chromatography (Hexane/ethyl acetate as eluent) to give the corresponding propargylic tosylate.

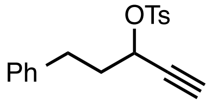

5-Phenylpent-1-yn-3-yl 4-methylbenzenesulfonate (1a-OTs). Prepared according to general procedure C utilizing alcohol (2.4 g, 15.0 mmol), TsCl (3.2 g, 16.5 mmol) and KOH (6.2 g, 111.0 mmol). The crude material was purified by silica gel chromatography (hexanes/ethyl acetate = 5:1) to afford the title compound as a light yellow oil (3.6 g, 73% yield).

^{1}H NMR (400 MHz, $CDCl_3$) δ 7.85 (d, *J* = 8.3 Hz, 2H), 7.36 (d, *J* = 8.1 Hz, 2H), 7.32 (t, *J* = 7.3 Hz, 2H), 7.24 (t, *J* = 7.4 Hz, 1H), 7.18 (d, *J* = 7.0 Hz, 2H), 5.10 (td, *J* = 6.6, 2.2 Hz, 1H), 2.87 – 2.73 (m, 2H), 2.51 (d, *J* = 2.3 Hz, 1H), 2.48 (s, 3H), 2.21 – 2.14 (m 2H);

^{13}C NMR (101 MHz, $CDCl_3$) δ 144.9, 139.9, 133.6, 129.6, 128.5, 128.3, 128.0, 126.2, 78.7, 76.6, 70.3, 37.2, 30.6, 21.6;

IR (neat) 3281, 3028, 2930, 2125, 1598, 1496, 1454, 1367, 1190, 1176, 1097, 900, 667 cm^{-1};

HRMS (ESI) Calcd. for $C_{18}H_{22}NO_3S$ ($[M+NH_4]^+$): 332.1320; Found: 332.1328.

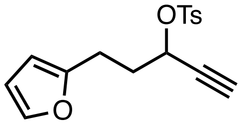

5-(Furan-2-yl)pent-1-yn-3-yl 4-methylbenzenesulfonate (1b-OTs). Prepared according to general procedure C utilizing alcohol (260 mg, 1.7 mmol), TsCl (364 mg, 1.9 mmol) and KOH (704 mg, 12.6 mmol). The crude material was purified by silica gel chromatography (hexanes/ethyl acetate = 10:1) to afford the title compound as a colorless oil (440 mg, 85% yield).

^{1}H NMR (400 MHz, $CDCl_3$) δ 7.81 (d, *J* = 8.2 Hz, 2H), 7.33 (d, *J* = 7.9 Hz, 2H), 7.28 (d, *J* = 2.2 Hz, 1H), 6.31 – 6.21 (m, 1H), 6.00 (d, *J* = 3.3 Hz, 1H), 5.09 (td, *J* = 6.5, 2.2 Hz, 1H), 2.87 – 2.74 (m, 2H), 2.44 (s, 3H), 2.20 – 2.12 (m, 2H);

[21] Condon-Gueugnot, S.; Linstrumelle, G. *Tetrahedron* **2000**, *56,* 1851–1857.

^{13}**C** NMR (101 MHz, $CDCl_3$) δ 153.5, 144.9, 141.3, 133.7, 129.7, 128.0, 110.2, 105.7, 78.5, 76.6, 70.0, 34.0, 23.2, 21.6;

IR (neat) 3286, 2930, 2126, 1597, 1367, 1177, 1190, 918, 668 cm^{-1};

HRMS (ESI) Calcd. for $C_{16}H_{17}O_4S$ ($[M+H]^+$): 305.0848; Found: 305.0863.

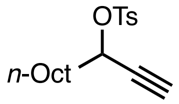

Undec-1-yn-3-yl 4-methylbenzenesulfonate (1c-OTs). Prepared according to general procedure C utilizing alcohol (672 mg, 4.0 mmol), TsCl (840 mg, 4.4 mmol) and KOH (1.66 g, 29.6 mmol). The crude material was purified by silica gel chromatography (hexanes/ethyl acetate = 6:1) to afford the title compound as a colorless oil (1.0 g, 84% yield). All spectral data are in accordance with the literature.[22]

^{1}**H** NMR (400 MHz, $CDCl_3$) δ 7.81 (d, *J* = 8.3 Hz, 2H), 7.32 (d, *J* = 8.0 Hz, 2H), 5.04 (td, *J* = 6.5, 2.1 Hz, 1H), 2.44 (s, 3H), 2.39 (d, *J* = 2.2 Hz, 1H), 1.84 – 1.77(m, 2H), 1.45 – 1.34 (m, 2H), 1.28– 1.23 (m, 10H), 0.87 (t, *J* = 6.8 Hz, 3H);

^{13}**C** NMR (101 MHz, $CDCl_3$) δ 144.8, 133.9, 129.6, 128.0, 79.1, 76.0, 71.1, 35.6, 31.8, 29.2, 29.1, 28.8, 24.5, 22.6, 21.6, 14.0;

IR (neat) 3280, 2927, 2856, 2125, 1598, 1369, 1189, 1177, 914, 668, 574 cm^{-1};

HRMS (ESI) Calcd. for $C_{18}H_{30}NO_3S$ ($[M+NH_4]^+$): 340.1963; Found: 340.1946.

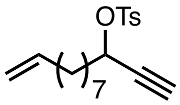

Dodec-11-en-1-yn-3-yl 4-methylbenzenesulfonate (1d-OTs). Prepared according to general procedure C utilizing alcohol (540 mg, 3.0 mmol), TsCl (630 mg, 3.3 mmol) and KOH (1.24 mg, 22.2 mmol). The crude material was purified by silica gel chromatography (hexanes/ethyl acetate = 20:1) to afford the title compound as a light yellow oil (649 mg, 65% yield).

^{1}**H** NMR (400 MHz, $CDCl_3$) δ 7.83 (d, *J* = 8.3 Hz, 2H), 7.35 (d, *J* = 8.2 Hz, 2H), 5.82 (ddt, *J* = 16.9, 10.2, 6.7 Hz, 2H), 5.06 (td, *J* = 6.6, 2.2 Hz, 1H), 5.01 (app. dq, *J* = 17.1, 1.7 Hz, 1H), 4.96 – 4.93 (m, 1H), 2.46 (s, 3H), 2.42 (d, *J* = 2.4 Hz, 1H), 2.05 (app. q, *J* = 7.3 Hz, 2H), 1.86 – 1.79 (m, 2H), 1.44 – 1.36 (m, 4H), 1.30 – 1.26 (m, 6H);

^{13}**C** NMR (101 MHz, $CDCl_3$) δ 144.8, 139.0, 133.9, 129.6, 128.0, 114.2, 79.1, 76.1, 71.1, 35.6, 33.7, 29.1, 28.8 (2C), 28.7, 24.4, 21.6;

IR (neat) 3290, 2928, 2856, 2125, 1598, 1369, 1189, 1177, 909, 668, 574 cm^{-1};

HRMS (ESI) Calcd. for $C_{19}H_{30}NO_3S$ ($[M+NH_4]^+$): 352.1946; Found: 352.1962.

[22] Zhang, Y; Hao, H.-D.; Wu, Y. *Synlett* **2010**, *6*, 905–908.

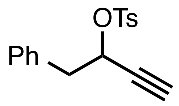

1-Phenylbut-3-yn-2-yl 4-methylbenzenesulfonate (1e-OTs). Prepared according to general procedure C utilizing alcohol (0.58 g, 4.0 mmol), TsCl (0.84 g, 4.4 mmol) and KOH (1.66 g, 29.6 mmol). The crude material was purified by silica gel chromatography (hexanes/ethyl acetate = 6:1) to afford the title compound as a white solid (0.8 g, 67% yield). Mp 64.2-64.3 °C.

^{1}H NMR (400 MHz, $CDCl_3$) δ 7.70 (d, *J* = 8.3 Hz, 2H), 7.29 – 7.27 (m, 5H), 7.21 – 7.19 (m, 2H), 5.21 (td, *J* = 6.8, 2.0 Hz, 1H), 3.18 (dd, *J* = 14.2, 6.5 Hz, 1H), 3.12 (dd, *J* = 14.1, 6.6 Hz, 1H), 2.50 (d, *J* = 2.3 Hz, 1H), 2.45 (s, 3H);

^{13}C NMR (101 MHz, $CDCl_3$) δ 144.7, 134.4, 133.4, 129.6, 129.6, 128.4, 127.9, 127.2, 78.6, 77.0, 71.2, 41.9, 21.6;

IR (neat) 3266, 2924, 2126, 1359, 1170, 992, 884, 771, 662 cm^{-1};

HRMS (ESI) Calcd. for $C_{17}H_{20}NO_3S$ ($[M+NH_4]^+$): 318.1164; Found: 318.1184.

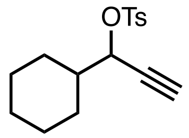

1-Cyclohexylprop-2-yn-1-yl 4-methylbenzenesulfonate (1f-OTs). Prepared according to general procedure C utilizing alcohol (548 mg, 4.0 mmol), TsCl (840 mg, 4.4 mmol) and KOH (1.66 g, 29.6 mmol). The crude material was purified by silica gel chromatography (hexanes/ethyl acetate = 10:1) to afford the title compound as a white solid (938 mg, 80% yield). Mp 67.3-67.9 °C.

^{1}H NMR (400 MHz, $CDCl_3$) δ 7.80 (d, *J* = 8.2 Hz, 2H), 7.32 (d, *J* = 8.1 Hz, 2H), 4.87 (dd, *J* = 6.0, 2.2 Hz, 1H), 2.43 (s, 3H), 2.37 (d, *J* = 2.3 Hz, 1H), 1.84 – 1.64 (m, 6H), 1.26 – 1.02 (m, 5H);

^{13}C NMR (101 MHz, $CDCl_3$) δ 144.7, 133.9, 129.6, 128.0, 78.0, 76.7, 75.3, 42.5, 28.0, 27.6, 25.9, 25.5, 25.4, 21.6;

IR (neat) 3266, 2925, 2125, 1359, 1174, 894, 849, 668 cm^{-1}.

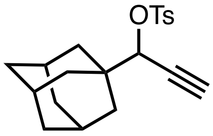

1-(Adamantyl)prop-2-yn-1-yl 4-methylbenzenesulfonate (1g-OTs). Prepared according to general procedure C utilizing alcohol (688 mg, 4.0 mmol), TsCl (840 mg, 4.4 mmol) and KOH (1.66 g, 29.6 mmol). The crude material was purified by silica gel chromatography (hexanes/ethyl acetate = 15:1) to afford the title compound as a colourless solid (1.08 g, 83% yield). Mp 98-99 °C.

^{1}H NMR (400 MHz, $CDCl_3$) δ 7.85 – 7.77 (m, 2H), 7.32 (d, *J* = 8.8 Hz, 2H), 4.63 (d, *J* = 2.2 Hz, 1H), 2.44 (s, 3H), 2.34 (d, *J* = 2.4 Hz, 1H), 1.99 (s, 3H), 1.73 – 1.55 (m, 12H);

^{13}C NMR (101 MHz, $CDCl_3$) δ 144.6, 134.0, 129.5, 128.1, 79.3, 77.1, 76.7 37.4, 37.2, 36.6, 27.9, 21.6;

IR (neat) 3296, 2912, 2852, 1362, 1174, 921, 849, 670 cm^{-1}.

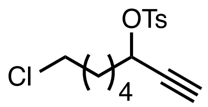

8-Chlorooct-1-yn-3-yl 4-methylbenzenesulfonate (1h-OTs). Prepared according to general procedure C utilizing alcohol (370mg, 2.3 mmol), TsCl (442 mg, 2.3 mmol) and KOH (953 mg, 17.0 mmol). The crude material was purified by silica gel chromatography (hexanes/ethyl acetate = 10:1) to afford the title compound as a colorless oil (575 mg, 80% yield).

^{1}H NMR (400 MHz, $CDCl_3$) δ 7.81 (d, *J* = 8.2 Hz, 2H), 7.33 (d, *J* = 8.0 Hz, 2H), 5.05 (td, *J* = 6.4, 2.1 Hz, 1H), 3.50 (t, *J* = 6.6 Hz, 2H), 2.44 (s, 3H), 2.40 (d, *J* = 2.4 Hz, 1H), 1.84 – 1.81 (m, 2H), 1.78 – 1.70 (m, 2H), 1.49 – 1.39 (m, 4H);

^{13}C NMR (101 MHz, $CDCl_3$) δ 144.9, 133.8, 129.7, 128.0, 78.8, 76.2, 70.8, 44.7, 35.4, 32.2, 26.0, 23.8, 21.7;

IR (neat) 3289, 2940, 2125, 1598, 1497, 1366, 1177, 1190, 897, 668 cm^{-1};

HRMS (ESI) Calcd. for $C_{15}H_{20}ClO_3S$ ($[M+H]^+$): 315.0822; Found: 315.0813.

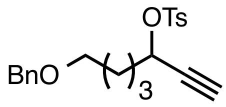

7-(Benzyloxy)hept-1-yn-3-yl 4-methylbenzenesulfonate (1i-OTs). Prepared according to general procedure C utilizing alcohol (1.27 g, 6.0 mmol), TsCl (1.26 g, 6.6 mmol) and KOH (2.48 g, 44.4 mmol). The crude material was purified by silica gel chromatography (hexanes/ethyl acetate = 6:1) to afford the title compound as a colorless solid (1.54 g, 73% yield). Mp 42.4-42.9 °C.

^{1}H NMR (400 MHz, $CDCl_3$) δ 7.84 (d, *J* = 8.4 Hz, 2H), 7.42 – 7.27 (m, 7H), 5.09 (td, *J* = 6.5, 2.2 Hz, 1H), 4.51 (s, 2H), 3.47 (t, *J* = 6.2 Hz, 2H), 2.46 (s, 3H), 2.43 (d, *J* = 2.0 Hz, 1H), 1.92 – 1.83 (m, 2H), 1.60 – 1.54 (m, 4H);

^{13}C NMR (101 MHz, $CDCl_3$) δ 144.8, 138.4, 133.8, 129.6, 128.3, 128.0, 127.5 (2C), 78.9, 76.2, 72.8, 70.9, 69.7, 35.4, 28.9, 21.6, 21.3;

IR (neat) 3274, 2947, 2361, 1357, 1172, 904, 860, 844, 740, 698, 670 cm^{-1};

HRMS (ESI) Calcd. for $C_{21}H_{25}O_4S$ ($[M+H]^+$): 373.1474; Found: 373.1458.

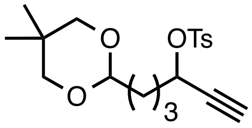

6-(5,5-Dimethyl-1,3-dioxan-2-yl)hex-1-yn-3-yl 4-methylbenzenesulfonate (1j-OTs). Prepared according to general procedure C utilizing alcohol (636 mg, 3.0 mmol), TsCl (630 mg, 3.3 mmol) and KOH (1.24

g, 22.2 mmol). The crude material was purified by silica gel chromatography (hexanes/ethyl acetate = 6:1) to afford the title compound as a colorless oil (0.76 g, 70% yield).

^{1}H NMR (400 MHz, $CDCl_3$) δ 7.78 (d, *J* = 8.3 Hz, 2H), 7.30 (d, *J* = 8.0 Hz, 2H), 5.02 (td, *J* = 6.4, 2.2 Hz, 1H), 4.35 (t, *J* = 4.6 Hz, 1H), 3.55 (d, *J* = 10.9 Hz, 2H), 3.37 (d, *J* = 10.9 Hz, 2H), 2.41 (s, 3H), 2.39 (d, *J* = 2.4 Hz, 1H), 1.89 – 1.77 (m, 2H), 1.62 – 1.47 (m, 4H), 1.14 (s, 3H), 0.68 (s, 3H);

^{13}C NMR (101 MHz, $CDCl_3$) δ 144.7, 133.7, 129.6, 128.0, 101.5, 78.9, 77.1, 76.2, 70.9, 35.4, 33.8, 30.0, 22.9, 21.7, 21.6, 19.0;

IR (neat) 3278, 2954, 2850, 2122, 1598, 1471, 1365, 1190, 1176, 900, 668 cm^{-1};

HRMS (ESI) Calcd. for $C_{19}H_{27}O_5S$ ($[M+H]^+$): 367.1579 ; Found: 367.1591.

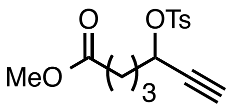

Methyl 5-(tosyloxy)hept-6-ynoate (1k-OTs). Prepared according to general procedure C utilizing alcohol (310 mg, 2.2 mmol), TsCl (468 mg, 2.5 mmol) and KOH (924 mg, 16.5 mmol). The crude material was purified by silica gel chromatography (hexanes/ethyl acetate = 6:1) to afford the title compound as a colorless oil (327 mg, 47% yield).

^{1}H NMR (400 MHz, $CDCl_3$) δ 7.80 (d, *J* = 8.3 Hz, 2H), 7.32 (d, *J* = 8.1 Hz, 2H), 5.06 (td, *J* = 6.1, 2.1 Hz, 1H), 3.65 (s, 3H), 2.43 (s, 3H), 2.41 (d, *J* = 2.3 Hz, 1H), 2.32 (t, *J* = 7.3 Hz, 2H), 1.90 – 1.72 (m, 4H);

^{13}C NMR (101 MHz, $CDCl_3$) δ 173.2, 144.9, 133.7, 129.6, 128.0, 78.5, 76.5, 70.4, 51.6, 34.8, 33.0, 21.6, 19.9;

IR (neat) 3278, 2953, 2124, 1736, 1598, 1367, 1190, 1177, 933, 901, 668. 574 cm^{-1};

HRMS (ESI) Calcd. for $C_{15}H_{22}NO_5S$ ($[M+NH_4]$): 328.1219; Found: 328.1233.

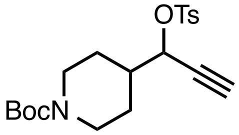

***tert*-Butyl 4-(1-(tosyloxy)prop-2-yn-1-yl)piperidine-1-carboxylate (1l-OTs)**. Prepared according to general procedure C utilizing alcohol (717m g, 3.0 mmol), TsCl (630 g, 3.3 mmol) and KOH (1.24 g, 22.2 mmol). The crude material was purified by silica gel chromatography (hexanes/ethyl acetate = 6:1) to afford the title compound as a colorless oil (850 mg, 72% yield).

^{1}H NMR (400 MHz, $CDCl_3$) δ 7.79 (d, *J* = 8.3 Hz, 2H), 7.32 (d, *J* = 8.1 Hz, 2H), 4.89 (dd, *J* = 6.3, 2.3 Hz, 1H), 4.13 (s, 2H), 2.62 (t, *J* = 8.3 Hz, 2H), 2.43 (s, 3H), 2.40 (d, *J* = 2.3 Hz, 1H), 1.87 (dddt, *J* = 12.0, 9.6, 6.6, 3.3 Hz, 1H), 1.79 – 1.68 (m, 2H), 1.42 (s, 9H), 1.37 – 1.18 (m, 2H);

^{13}C NMR (101 MHz, $CDCl_3$) δ 154.6, 144.9, 129.6, 128.0, 79.5, 77.4, 77.2, 74.1, 43.1, 41.0, 28.3, 27.3, 26.8, 21.6;

IR (neat) 3276, 2976, 2930, 2123, 1690, 1424, 1366, 1176, 937, 902, 669 cm^{-1};

HRMS (ESI) Calcd. for $C_{20}H_{28}NO_5S$ ($[M+H]^+$): 394.1688; Found: 394.1693.

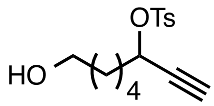

8-Hydroxyoct-1-yn-3-yl 4-methylbenzenesulfonate (1m-OTs). In air, TsCl (2.1 g, 11.0 mmol) and propargylic alchol (2.6 g, 10.0 mmol) were added in turn to a round-bottom flask with a 2.0 cm stir bar. Et_2O (50 mL) was added to dissolve the solid and then the reaction mixture was cooled at –10 °C. Next, freshly fine powdered KOH (4.1 g, 74.0 mmol) was added portionwise by spatula while keeping temperature below –10 °C. The mixture was stirred at –5 °C for 1 h before poured into ice water (50 mL). After vigorous shaking, the layers were separated and the aqueous layer was extracted twice with Et_2O (50 mL). The combined organic layers concentrated under reduced pressure. The residue was dissolved in THF (60 mL) in a round-bottom flask with a 2.0 cm stir bar. HCl (2 M, aq., 30 mL) was added dropwise via pipette at 0 °C. The reaction mixture was then stirred at room temperature for 1 h. H_2O (20 mL) and Et_2O (60 mL) were added to dilute the mixture. After the organic phase was separated, the aqueous phase was extracted twice with Et_2O (60 mL). The combined organic phases were washed with brine, dried over $MgSO_4$, filtered and then concentrated under reduced pressure. The crude oil was purified by silica gel chromatography (hexane/EtOAc = 2:1) to afford the product as a colorless oil (2.2 g, 75% yield).

^{1}H NMR (400 MHz, $CDCl_3$) δ 7.78 (d, *J* = 8.4 Hz, 2H), 7.31 (d, *J* = 8.0 Hz, 2H), 5.03 (td, *J* = 6.6, 2.2 Hz, 1H), 3.58 (t, *J* = 6.5 Hz, 2H), 2.42 (s, 3H), 2.40 (d, *J* = 2.1 Hz, 1H), 1.87 – 1.73 (m, 3H), 1.57 – 1.26 (m, 6H);

^{13}C NMR (101 MHz, $CDCl_3$) δ 144.8, 133.6, 129.6, 127.9, 78.8, 76.2, 70.9, 62.4, 35.5, 32.2, 24.9, 24.2, 21.6;

IR (neat) 3565, 3296, 2937, 2865, 2124, 1598, 1364, 1190, 1177, 911, 734, 669 cm^{-1}.

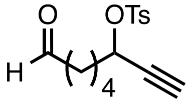

8-Oxooct-1-yn-3-yl 4-methylbenzenesulfonate (1n-OTs). A 100 mL round-bottom flask with a 1.5 cm stir bar was evacuated and refilled with N_2 three times. $(COCl)_2$ (0.2 mL, 2.2 mmol) and CH_2Cl_2 (5 mL) was added via syringe. The flask was cooled to –70 °C. A solution of DMSO (0.3 mL, 4.4 mmol) in CH_2Cl_2 (5 mL) was added to the flask by syringe at –70 °C. After addition, the mixture was stirred at this temperature for 10 min before a solution of alcohol (592 mg, 2.0 mmol) in CH_2Cl_2 (5 mL) was added dropwise. The mixture was stirred at –70 °C for further 0.5 h. Then Et_3N (1.5 mL, 10.6 mmol) was added once. After that, the cooling bath was removed and the mixture was allowed to reach room temperature. H_2O (20 mL) was added and the aqueous phase was extracted twice with CH_2Cl_2 (60 mL). The combined organic phases were washed with brine, dried over $MgSO_4$, filtered and then concentrated under reduced pressure. The crude oil was purified by silica gel chromatography (hexane/EtOAc = 3:1) to afford the product as a colorless oil (500 mg, 85% yield).

^{1}H NMR (400 MHz, $CDCl_3$) δ 9.71 (br. s, 1H), 7.78 (d, *J* = 8.2 Hz, 2H), 7.31 (d, *J* = 8.0 Hz, 2H), 5.03 (td, *J* = 6.4, 2.2 Hz, 1H), 2.43 – 2.38 (m, 6H), 1.90 – 1.77 (m, 2H), 1.65 – 1.58 (m, 2H), 1.50 – 1.38 (m, 2H);

^{13}C NMR (101 MHz, $CDCl_3$) δ 201.9, 144.9, 133.6, 129.6, 127.9, 78.6, 76.3, 70.6, 43.4, 35.3, 23.9, 21.6, 21.1;

IR (neat) 3281, 2951, 2124, 1722, 1365, 1190, 1177, 1096, 897, 668 cm^{-1};

HRMS (ESI) Calcd. for $C_{15}H_{22}NO_4S$ ($[M+NH_4]^+$): 312.1268; Found: 312.1270.

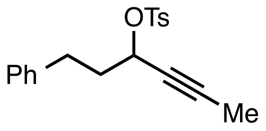

1-Phenylhex-4-yn-3-yl 4-methylbenzenesulfonate (6). Prepared according to general procedure C utilizing alcohol (522 mg, 3.0 mmol), TsCl (630 mg, 3.3 mmol) and KOH (1.24 g, 22.2 mmol). The crude material was purified by silica gel chromatography (hexanes/ethyl acetate = 10:1) to afford the title compound as a colorless oil (676 mg, 68% yield).

^{1}H NMR (400 MHz, $CDCl_3$) δ 7.84 (d, *J* = 8.3 Hz, 2H), 7.36 (d, *J* = 8.1 Hz, 2H), 7.31 (dd, *J* = 7.9, 6.4 Hz, 2H), 7.24 (d, *J* = 7.1 Hz, 1H), 7.19 – 7.15 (m, 2H), 5.09 (ddq, *J* = 6.4, 4.2, 2.1 Hz, 1H), 2.79 – 2.74 (m, 2H), 2.48 (s, 3H), 2.15 – 2.08 (m, 2H), 1.69 (d, *J* = 2.1 Hz, 3H);

^{13}C NMR (101 MHz, $CDCl_3$) δ 144.5, 140.3, 134.3, 129.4, 128.4 (2C), 128.3, 128.1, 126.1, 85.4, 74.5, 71.8, 37.5, 30.8, 21.6, 3.4;

IR (neat) 3028, 2922, 2248, 1599, 1364, 1189, 1176, 893, 667, 566 cm^{-1};

HRMS (ESI) Calcd. for $C_{19}H_{24}NO_3S$ ($[M+NH_4]^+$): 346.1477; Found: 346.1453.

4.3 General Procedure D: Preparation of Tertiary Propargylic Trichloroacetimidates[23]

NaH[24] (1.0 eq, a 60% dispersion in oil, which had been previously washed twice with hexane) was added to a flask with a stir bar. The flask was evacuated and refilled with N_2 three times. Then THF (2.0 M) was added to the flask, followed by a solution of tertiary propargylic alcohol (1.0 eq) in THF (1.0 M) at room temperature. After the mixture was stirred for 30 min at room temperature, the yellow alkoxide solution was added dropwise to a solution of freshly distilled trichloroacetonitrile (2.0 eq) in THF (2.0 M) at 0 °C. The resulting brown solution was stirred at 0 °C for 1 h and then concentrated to afford a dark oil. Pentane (the same volume of THF, containing 1% methanol) was added, and a small amount of dark, insoluble material was removed by filtration through Celite. The filtrate was concentrated under reduced pressure and the crude oil was purified by silica gel chromatography (Hexane/ethyl acetate + 1% Et_3N) to give the corresponding tertiary propargylic trichloroacetimidate. Yields have not been optimized.

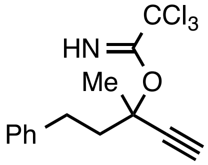

3-methyl-5-phenylpent-1-yn-3-yl 2,2,2-trichloroacetimidate (4a). Prepared according to general procedure D utilizing alcohol (1.74 g, 10.0 mmol), NaH (240 mg, 10.0 mmol) and Cl_3CCN (2.0 mL, 20.0 mmol). The crude material was purified by silica gel chromatography (hexanes/ethyl acetate = 40:1+1% triethylamine) to afford the title compound as a yellow solid (860 mg, 27% yield). Mp 42-43 °C.

^{1}H NMR (400 MHz, $CDCl_3$) δ 8.63 (s, 1H), 7.34 (app. t, J = 7.4 Hz, 2H), 7.29 – 7.23 (m, 3H), 3.00 – 2.94 (m, 2H), 2.73 (s, 1H), 2.46 – 2.27 (m, 2H), 1.93 (s, 3H);

^{13}C NMR (101 MHz, $CDCl_3$) δ 159.6, 141.3, 128.4 (2C), 126.0, 92.0, 82.6, 78.1, 74.6, 43.8, 30.4, 25.5;

IR (neat) 3321, 3271, 2966, 2116, 1666, 1309, 1067, 842, 795, 685 cm^{-1};

HRMS (ESI) Calcd. for $C_{14}H_{15}NOCl_3$ ($[M+H]^+$): 318.0219; Found: 318.0224.

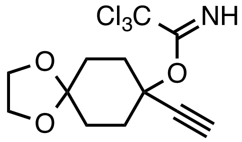

8-Ethynyl-1,4-dioxaspiro[4.5]decan-8-yl 2,2,2-trichloroacetimidate (4b). Prepared according to general procedure D utilizing alcohol (910 mg, 5.0 mmol), NaH (120 mg, 5.0 mmol) and Cl_3CCN (1.0 mL, 10.0 mmol). The crude material was purified by silica gel chromatography (hexanes/ethyl acetate = 8:1+1% triethylamine) to afford the title compound as a yellow solid (500 mg, 31% yield). Mp 36-37 °C.

[23] Overman, L. E.; Clizbe, L. A.; Freerks, R. L. *J. Am. Chem. Soc.* **1981**, *103*, 2807–2815.
[24] Catalytic amount of NaH gave trace product.

^{1}H NMR (400 MHz, $CDCl_3$) δ 8.56 (s, 1H), 3.95 (s, 4H), 2.65 (s, 1H), 2.58 – 2.40 (m, 2H), 2.31 – 2.18 (m, 2H), 1.87 – 1.73 (m, 4H);

^{13}C NMR (101 MHz, $CDCl_3$) δ 159.6, 107.4, 91.8, 82.2, 74.8, 64.4, 64.3, 33.9, 30.6;

IR (neat) 3244, 2961, 2889, 2116, 1666, 1310, 1244, 1066, 922, 794 cm^{-1};

HRMS (ESI) Calcd. for $C_{12}H_{15}NO_3Cl_3$ ($[M+H]^+$): 326.0125; Found: 318.0224.

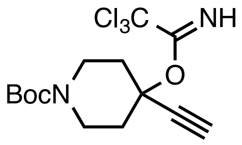

***tert*-Butyl 4-ethynyl-4-(2,2,2-trichloro-1-iminoethoxy)piperidine-1-carboxylate (4c).** Prepared according to general procedure D utilizing alcohol (750 mg, 3.3 mmol), NaH (80 mg, 3.3 mmol) and Cl_3CCN (0.7 mL, 6.7 mmol). The crude material was purified by silica gel chromatography (hexanes/ethyl acetate = 8:1+1% triethylamine) to afford the title compound as a white solid (552 mg, 45% yield). Mp 64-65 °C.

^{1}H NMR (400 MHz, $CDCl_3$) δ 8.60 (s, 1H), 3.54 (app. t, J = 5.7 Hz, 4H), 2.70 (s, 1H), 2.29 – 2.15 (m, 4H), 1.44 (s, 9H);

^{13}C NMR (101 MHz, CDCl3) δ 159.3, 154.4, 91.6, 81.4, 79.8, 76.2, 75.8, 40.1, 35.8, 28.3;

IR (neat) 3241, 2975, 2877, 2107, 1674, 1423, 1302, 1148, 1063, 1022, 793 cm^{-1};

HRMS (ESI) Calcd. for $C_{14}H_{20}N_2O_3Cl_3$ ($[M+H]^+$): 369.0540; Found: 369.0549.

5. General Procedure E: Fluorination of Secondary Propargylic Tosylates (Table 2)

Propargylic tosylate (0.5 mmol) and (IPr)CuCl (0.05 mmol) were added in turn to a 4 mL vial with a 1 cm stir bar. The vial was capped with a PTFE-lined cap and then it was evacuated and refilled with N_2 three times. Then THF (2.5 mL) was added via 5 mL syringe to dissolve the solid, followed by Et_3N•3HF (0.25 mL, 1.5 mmol) added in one portion via 1 mL syringe at room temperature. The reaction mixture was then stirred at 30 °C. After 24 h, the mixture was transferred to a 25 mL round-bottom flask via pipette and solvent was removed under reduced pressure. The residue was purified by silica gel chromatography (hexane/ethyl acetate) to afford the corresponding propargylic fluoride.

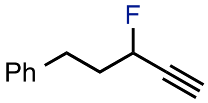

(3-Fluoropent-4-yn-1-yl)benzene (2a). Prepared according to general procedure E. The crude material was purified by silica gel chromatography (hexanes/ethyl acetate = 10:1) to afford the title compound as a colorless oil.

First run: 68 mg (84% yield). Second run: 73 mg (90% yield);

^{1}H NMR (400 MHz, $CDCl_3$) δ 7.39 – 7.35 (m, 2H), 7.30 – 7.26 (m, 3H), 5.14 (dddd, J = 48.0, 7.2, 5.3, 2.1 Hz, 1H), 2.96 – 2.81 (m, 2H), 2.76 (dd, J = 5.6, 2.1 Hz, 1H), 2.38 – 2.10 (m, 2H);

^{13}C NMR (101 MHz, $CDCl_3$) δ 140.4, 128.5, 128.4, 126.2, 81.5 (d, J = 167.6 Hz), 80.1(d, J = 25.3 Hz), 76.6 (d, J = 10.8 Hz), 37.3 (d, J = 22.6 Hz), 30.5 (d, J = 4.8 Hz);

^{19}F {^{1}H} NMR (377 MHz, $CDCl_3$) δ –177.4;

IR (neat) 3298, 2126, 1603, 1496, 1455, 1030, 992, 746, 699 cm^{-1};

HRMS (EI) Calcd. for $C_{11}H_{11}F$ ($[M]^+$): 162.0845; Found: 162.0837.

Gram Scale: 5-Phenylpent-1-yn-3-yl 4-methylbenzenesulfonate (3.14 g, 10 mmol) and (IPr)CuCl (487 mg, 1 mmol) were added in turn to a 100 mL round-bottom flask with a 2 cm stir bar. The flask was evacuated and refilled with N_2 three times. Then THF (50 mL) was added via syringe to dissolve the solid, followed by Et_3N•3HF (5 mL, 30 mmol) added in one portion via syringe at room temperature. The reaction mixture was then stirred at 30 °C. After 24 h, the solvent was removed under reduced pressure. The residue was purified by silica gel chromatography (hexane/ethyl acetate hexanes/ethyl acetate = 10:1) to afford the corresponding (3-Fluoropent-4-yn-1-yl)benzene as colorless oil (1.46 g, 90% yield).

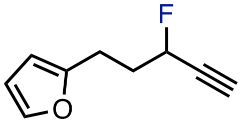

2-(3-Fluoropent-4-yn-1-yl)furan (2b). Prepared according to general procedure E. The crude material was purified by silica gel chromatography (hexanes/ethyl acetate = 20:1) to afford the title compound as a colorless oil.

First run: 46 mg (61% yield). Second run: 43 mg (57% yield);

^{1}H NMR (400 MHz, $CDCl_3$) δ 7.32 (d, *J* = 1.6 Hz, 1H), 6.34 – 6.24 (m, 1H), 6.13 – 5.96 (m, 1H), 5.13 (dddd, *J* = 48.0, 7.3, 5.7, 2.0 Hz, 1H), 2.92 – 2.78 (m, 2H), 2.70 (dd, *J* = 5.4, 2.0 Hz, 1H), 2.33 – 2.10 (m, 2H);

^{13}C NMR (101 MHz, $CDCl_3$) δ 154.0, 141.3, 110.2, 105.6, 81.4 (d, *J* = 166.6 Hz), 79.8 (d, *J* = 25.7 Hz), 76.7 (d, *J* = 9.9 Hz), 34.2 (d, *J* = 25.0 Hz), 23.0 (d, *J* = 4.3 Hz);

^{19}F {^{1}H} NMR (377 MHz, $CDCl_3$) δ –177.3;

IR (neat) 3298, 2935, 2127, 1599, 1508, 1344, 1146, 1038, 1006, 735, 650 cm^{-1};

HRMS (EI) Calcd. for C_9H_9FO ($[M]^+$): 152.0637; Found: 152.064.

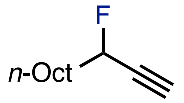

3-Fluoroundec-1-yne (2c).[25] Prepared according to general procedure E. The crude material was purified by silica gel chromatography (hexanes) to afford the title compound as a colorless oil.

First run: 73 mg (86% yield). Second run: 76 mg (89% yield);

^{1}H NMR (400 MHz, $CDCl_3$) δ 5.08 (br. dt, *J* = 48.3, 6.3 Hz, 1H), 2.65 (d, *J* = 5.7 Hz, 1H), 1.91 – 1.77 (m, 2H), 1.54 – 1.42 (m, 2H), 1.33 – 1.27 (m, 10H), 0.88 (t, *J* = 6.6 Hz, 3H);

^{13}C NMR (101 MHz, $CDCl_3$) δ 82.4 (d, *J* = 166.0 Hz), 80.5(d, *J* = 25.6 Hz), 76.1 (d, *J* = 10.8 Hz), 35.8 (d, *J* = 21.9 Hz), 31.8, 29.4, 29.2, 29.1, 24.3 (d, *J* = 4.2 Hz), 22.6, 14.1;

^{19}F {^{1}H} NMR (377 MHz, $CDCl_3$) δ –175.0;

IR (neat) 3310, 2927, 2857, 2126, 1467, 1345, 992, 673, 634 cm^{-1}.

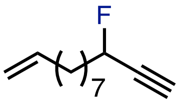

10-Fluorododec-1-en-11-yne (2d). Prepared according to general procedure E. The crude material was purified by silica gel chromatography (hexanes/ethyl acetate = 20:1) to afford the title compound as a colorless oil. All spectral data are in accordance with the literature.[17]

First run: 84 mg (92% yield). Second run: 80 mg (88% yield);

^{1}H NMR (400 MHz, $CDCl_3$) δ 5.81 (ddt, *J* = 16.8, 10.1, 6.5 Hz, 1H), 5.20 – 4.81 (m, 3H), 2.70 – 2.62 (m, 1H), 2.04 (app. q, *J* = 6.9 Hz, 2H), 1.89 – 1.78 (m, 2H), 1.53 – 1.25 (m, 10H);

^{13}C NMR (101 MHz, $CDCl_3$) δ 139.1, 114.2, 82.4 (d, *J* = 166.0 Hz), 80.4 (d, *J* = 25.7 Hz), 76.1 (d, *J* = 10.8 Hz), 35.7 (d, *J* = 21.8 Hz), 33.7, 29.2, 29.0, 28.9, 28.8, 24.3 (d, *J* = 4.5 Hz);

^{19}F {^{1}H} NMR (377 MHz, $CDCl_3$) δ –175.1;

IR (neat) 3305, 2928, 2857, 2127, 1640, 1466, 995, 911, 675, 638 cm^{-1}.

---

[25] Jiang, H.; Falcicchio, A.; Jensen, K.L.; Paixão, M.W.; Bertelsen, S.; Jørgensen, K. A. *J. Am. Chem. Soc.* **2009**, *131*, 7153–7157.

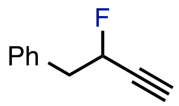

(2-Fluorobut-3-yn-1-yl)benzene (2e). Prepared according to general procedure E. The crude material was purified by silica gel chromatography (hexanes/ethyl acetate = 20:1) to afford the title compound as a colorless oil. All spectral data are in accordance with the literature.[17]

First run: 51 mg (69% yield). Second run: 49 mg (66% yield);

^{1}H NMR (400 MHz, CDCl3) δ 7.41 – 7.28 (m, 5H), 5.39 – 5.18 (m, 1H), 3.29 – 3.10 (m, 3H), 2.71 (dd, *J* = 5.6, 2.2 Hz, 1H);

^{13}C NMR (101 MHz, CDCl3) δ 135.0, 129.6, 128.4, 127.1, 82.5 (d, *J* = 170.0 Hz), 79.8(d, *J* = 25.8 Hz), 77.1 (d, *J* = 10.4 Hz), 42.2 (d, *J* = 22.6Hz);

^{19}F {^{1}H} NMR (377 MHz, $CDCl_3$) δ –173.0;

IR (neat) 3297, 3032, 2929, 2127, 1496, 1455, 1008, 746, 700 cm^{-1};

HRMS (EI) Calcd. for $C_{10}H_9F$ ($[M]^+$): 148.0688; Found: 148.0686.

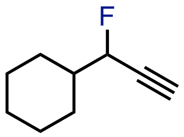

(1-Fluoroprop-2-yn-1-yl)cyclohexane (2f). Prepared according to general procedure E. The crude material was purified by silica gel chromatography (hexanes) to afford the title compound as a colorless oil.

First run: 63 mg (90% yield). Second run: 60 mg (86% yield);

^{1}H NMR (400 MHz, $CDCl_3$) δ 4.85 (ddd, *J* = 48.0, 5.9, 2.1 Hz, 1H), 2.66 (dd, *J* = 5.7, 2.2 Hz, 1H), 1.92 – 1.65 (m, 6H), 1.33 – 1.04 (m, 5H);

^{13}C NMR (101 MHz, $CDCl_3$) δ 86.5 (d, *J* = 168.7 Hz), 79.5(d, *J* = 26.7 Hz), 76.8 (d, *J* = 10.4 Hz), 42.7(d, *J* = 20.6 Hz), 27.5, 26.1, 25.6;

^{19}F {^{1}H} NMR (377 MHz, $CDCl_3$) δ –180.8;

IR (neat) 3305, 2931, 2856, 2125, 1451, 1336, 1005, 986, 736, 684 cm^{-1};

HRMS (EI) Calcd. for C_9H_{13} ($[M\text{-}F]^+$): 121.1017; Found: 121.1011.

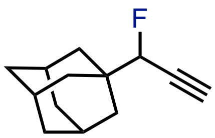

1-(1-fluoroprop-2-yn-1-yl)adamantine (2g). Prepared according to general procedure E. The crude material was purified by silica gel chromatography (hexanes/ethyl acetate = 20:1) to afford the title compound as a colorless oil.

First run: 78 mg (81% yield). Second run: 91 mg (95% yield);

^{1}**H** NMR (400 MHz, $CDCl_3$) δ 4.57 (dd, *J* = 48.0, 2.3 Hz, 1H), 2.65 (dd, *J* = 5.8, 2.4 Hz, 1H), 2.03 (app. quin., *J* = 3.2 Hz, 3H), 1.77 – 1.58 (m, 12H);

^{13}**C** NMR (101 MHz, $CDCl_3$) δ 90.2 (d, *J* = 171.4 Hz), 78.3 (d, *J* = 26.1 Hz), 77.2 (d, *J* = 10.2 Hz), 37.0 (2C), 36.8, 27.9;

^{19}**F {^{1}H}** NMR (377 MHz, $CDCl_3$) δ –189.3;

IR (neat) 3286, 2906, 2850, 2118, 1452, 1344, 1018, 666, 657 cm^{-1}.

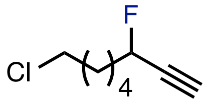

8-Chloro-3-fluorooct-1-yne (2h). Prepared according to general procedure E. The crude material was purified by silica gel chromatography (hexanes/ethyl acetate = 20:1) to afford the title compound as a colorless oil.

First run: 67 mg (83% yield). Second run: 60 mg (74% yield);

^{1}**H** NMR (400 MHz, $CDCl_3$) δ 5.10 (br. dt, *J* = 47.9, 6.2 Hz, 1H), 3.54 (t, *J* = 6.6 Hz, 2H), 2.67 (dd, *J* = 5.6, 1.6 Hz, 1H), 1.91– 1.76 (m, 4H), 1.56 – 1.48 (m, 4H);

^{13}**C** NMR (101 MHz, $CDCl_3$) δ 82.2 (d, *J* = 166.4 Hz), 80.2 (d, *J* = 26.0 Hz), 76.3 (d, *J* = 10.6 Hz), 44.8, 35.5 (d, *J* = 22.1 Hz), 32.3, 26.3, 23.6 (d, *J* = 4.3 Hz);

^{19}**F {^{1}H}** NMR (377 MHz, $CDCl_3$) δ –175.3;

IR (neat) 3299, 2946, 2867, 2126, 1463, 1346, 991, 735, 650 cm^{-1}.

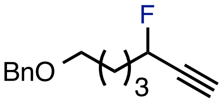

(((5-Fluorohept-6-yn-1-yl)oxy)methyl)benzene (2i). Prepared according to general procedure E. The crude material was purified by silica gel chromatography (hexanes/ethyl acetate = 20:1) to afford the title compound as a colorless oil.

First run: 95 mg (89% yield). Second run: 105 mg (98% yield);

^{1}**H** NMR (400 MHz, $CDCl_3$) δ 7.40 – 7.27 (m, 5H), 5.11 (dtd, *J* = 48.3, 6.4, 2.2 Hz, 1H), 4.53 (s, 2H), 3.51 (t, *J* = 6.2 Hz, 2H), 2.69 (dd, *J* = 5.8, 2.0 Hz, 1H), 2.00 – 1.81 (m, 2H), 1.74 – 1.56 (m, 4H);

^{13}**C** NMR (101 MHz, $CDCl_3$) δ 138.4, 128.3, 127.6, 127.5, 82.2 (d, *J* = 166.2 Hz), 80.3 (d, *J* = 25.8 Hz), 76.3 (d, *J* = 10.5 Hz), 72.9, 69.9, 35.5 (d, *J* = 22.3 Hz), 29.1, 21.1 (d, *J* = 5.0 Hz);

^{19}**F {^{1}H}** NMR (377 MHz, CDCl3) δ –175.1;

IR (neat) 3297, 3028, 2990, 2122, 1497, 1454, 1094, 884, 698 cm^{-1};

HRMS (EI) Calcd. for $C_{14}H_{17}FO$ ($[M]^+$): 220.1263; Found: 220.1271.

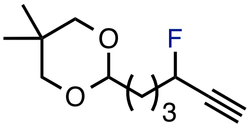

2-(4-Fluorohex-5-yn-1-yl)-5,5-dimethyl-1,3-dioxane (2j). Prepared according to general procedure E. The crude material was purified by silica gel chromatography (hexanes/ethyl acetate = 20:1) to afford the title compound as a colorless oil.

First run: 85 mg (82% yield). Second run: 85 mg (82% yield);

^{1}**H** NMR (400 MHz, $CDCl_3$) δ 5.05 (br. dt, J = 48.2, 6.2 Hz, 1H), 4.40 (t, J = 4.3 Hz, 1H), 3.56 (d, J = 11.2 Hz, 2H), 3.39 (d, J = 10.8 Hz, 2H), 2.65 (dd, J = 5.7, 2.1 Hz, 1H), 1.94 – 1.51 (m, 6H), 1.15 (s, 3H), 0.69 (s, 3H);

^{13}**C** NMR (101 MHz, $CDCl_3$) δ 101.7, 82.2 (d, J = 165.9 Hz), 80.2 (d, J = 25.5 Hz), 77.1 (2C), 76.3 (d, J = 10.4 Hz), 35.6 (d, J = 22.3 Hz), 34.1, 30.1, 22.9, 21.8, 18.9 (d, J = 4.8 Hz);

^{19}**F {^{1}H}** NMR (377 MHz, $CDCl_3$) δ –175.3;

IR (neat) 3299, 2956, 2849, 2124, 1471, 1395, 1139, 1113, 1041, 910, 666 cm^{-1};

HRMS (EI) Calcd. for $C_{12}H_{19}FO_2$ ($[M]^+$): 214.1369; Found: 214.1360.

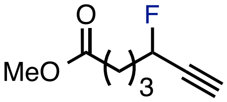

Methyl 5-fluorohept-6-ynoate (2k). Prepared according to general procedure E. The crude material was purified by silica gel chromatography (hexanes/ethyl acetate = 20:1) to afford the title compound as a colorless oil.

First run: 63 mg (80% yield). Second run: 64 mg (81% yield);

^{1}**H** NMR (400 MHz, $CDCl_3$) δ 5.08 (dtd, J = 47.6, 5.9, 2.2 Hz, 1H), 3.64 (s, 3H), 2.72 – 2.62 (m, 1H), 2.35 (t, J = 7.2 Hz, 2H), 1.95 – 1.72 (m, 4H);

^{13}**C** NMR (101 MHz, $CDCl_3$) δ 173.3, 81.9 (d, J = 166.9 Hz), 79.8 (d, J = 25.7 Hz), 76.6 (d, J = 10.4 Hz), 51.5, 34.9 (d, J = 22.4 Hz), 33.1, 19.7 (d, J = 3.5 Hz);

^{19}**F {^{1}H}** NMR (377 MHz, $CDCl_3$) δ –175.7;

IR (neat) 3297, 2955, 2125, 1737, 1438, 1252, 1201, 1165, 994, 687 cm^{-1};

HRMS (EI) Calcd. for $C_8H_{11}FO_2$ ($[M]^+$): 158.0743; Found: 158.0736.

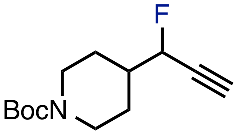

***tert*-Butyl 4-(1-fluoroprop-2-yn-1-yl)piperidine-1-carboxylate (2l)**. Prepared according to general procedure E. The crude material was purified by silica gel chromatography (hexanes/ethyl acetate = 3:1) to afford the title compound as a colorless oil.

First run: 100 mg (83% yield). Second run: 96 mg (79% yield);

^{1}H NMR (400 MHz, $CDCl_3$) δ 4.90 (ddd, *J* = 47.9, 6.1, 2.1 Hz, 1H), 4.18 (br. d, *J* = 13.3 Hz, 2H), 2.78 – 2.59 (m, 3H), 1.98 – 1.72 (m, 3H), 1.45 (s, 9H), 1.40 – 1.26 (m, 2H);

^{13}C NMR (101 MHz, $CDCl_3$) δ 154.6, 85.3 (d, *J* = 169.3 Hz), 79.4, 78.5(d, *J* = 26.0 Hz), 77.5 (d, *J* = 10.8 Hz), 43.1, 41.1(d, *J* = 21.6 Hz), 28.3, 26.6, 26.7;

^{19}F {^{1}H} NMR (377 MHz, $CDCl_3$) δ –181.2;

IR (neat) 3301, 3234, 2977, 2931, 2122, 1689, 1424, 1171, 1008, 980, 735 cm^{-1}.

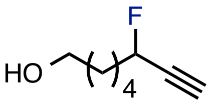

6-Fluorooct-7-yn-1-ol (2m). Prepared according to general procedure E. The crude material was purified by silica gel chromatography (hexanes/ethyl acetate = 3:1) to afford the title compound as a colorless oil.

First run: 42 mg (58% yield). Second run: 38 mg (53% yield);

^{1}H NMR (400 MHz, $CDCl_3$) δ 5.08 (dtd, *J* = 48.3, 6.2, 2.2 Hz, 1H), 3.65 (t, *J* = 6.5 Hz, 2H), 2.66 (dd, *J* = 5.6, 2.1 Hz, 1H), 1.95 – 1.76 (m, 2H), 1.65 – 1.39 (m, 7H);

^{13}C NMR (101 MHz, $CDCl_3$) δ 82.3(d, *J* = 166.0 Hz), 80.3(d, *J* = 25.6 Hz), 76.2 (d, *J* = 9.8 Hz), 62.6, 35.6 (d, *J* = 21.7 Hz), 32.4, 25.2, 24.1 (d, *J* = 4.6 Hz);

^{19}F {^{1}H} NMR (377 MHz, $CDCl_3$) δ –175.1;

IR (neat) 3300, 2939, 2865, 2124, 1345, 1057, 1001, 737, 677, 646 cm^{-1};

HRMS (CI) Calcd. for $C_8H_{17}NOF$ ($[M+NH_4]^+$): 162.1294; Found: 162.1295.

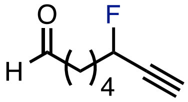

6-Fluorooct-7-ynal (2n). Prepared according to general procedure E. The crude material was purified by silica gel chromatography (hexanes/ethyl acetate = 6:1) to afford the title compound as a colorless oil.

First run: 60 mg (85% yield). Second run: 64 mg (90% yield);

^{1}H NMR (400 MHz, $CDCl_3$) δ 9.76 (br. s, 1 H), 5.09 (dtd, *J* = 48.0, 6.1, 2.1 Hz, 1H), 2.67 (dd, *J* = 5.7, 2.2 Hz, 1H), 2.47 (td, *J* = 7.3, 1.5 Hz, 2H), 1.93 – 1.81 (m, 2H), 1.72 – 1.63 (m, 2H), 1.58 – 1.48 (m, 2H);

^{13}C NMR (101 MHz, $CDCl_3$) δ 202.1, 82.0 (d, *J* = 166.2 Hz), 80.0 (d, *J* = 25.7 Hz), 76.4 (d, *J* = 11.0 Hz), 43.6, 35.4(d, *J* = 22.3 Hz), 23.9 (d, *J* = 4.5 Hz), 21.4;

^{19}F {^{1}H} NMR (377 MHz, $CDCl_3$) δ –175.4;

IR (neat) 3298, 2952, 2124, 1711, 1348, 1140, 990, 735, 680, 649 cm^{-1};

HRMS (CI) Calcd. for $C_8H_{15}NOF$ ($[M+NH_4]^+$): 160.1146; Found: 160.1138.

6. General Procedure F: Fluorination of Tertiary Propargylic Trichloroacetimidates (Scheme 1)

Propargylic trichloroacetimidate (0.5 mmol) and (IPr)CuOTf (0.05 mmol) were added in turn to a 4 mL vial with a 1 cm stir bar. The vial was capped with a PTFE-lined cap and then it was evacuated and refilled with N_2 three times. Then THF (2.5 mL) was added via 5 mL syringe to dissolve the solid, followed by $Et_3N{\cdot}3HF$ (0.25 mL, 1.5 mmol) added in one portion via 1 mL syringe at room temperature. The reaction mixture was then stirred at 30 °C. After 2 h, the mixture was transferred to a 25 mL round-bottom flask via pipette and solvent was removed under reduced pressure. The residue was purified by silica gel chromatography (hexane/ethyl acetate) to afford the corresponding tertiary propargylic fluoride.

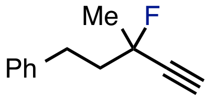

(3-Fluoro-3-methylpent-4-yn-1-yl)benzene (5a). Prepared according to general procedure F. The crude material was purified by silica gel chromatography (hexanes) to afford the title compound as a colorless oil.

First run: 54 mg (61% yield). Second run: 58 mg (66% yield);

^{1}H NMR (400 MHz, $CDCl_3$) δ 7.38 – 7.31 (m, 2H), 7.28 – 7.24 (m, 3H), 2.94 (t, J = 8.8 Hz, 2H), 2.74 (d, J = 5.1 Hz, 1H), 2.29 – 2.04 (m, 2H), 1.73 (d, J = 20.7 Hz, 3H);

^{13}C NMR (101 MHz, $CDCl_3$) δ 141.2, 128.5, 128.3, 126.0, 89.4 (d, J = 166.8 Hz), 83.0 (d, J = 29.7 Hz), 74.7 (d, J = 8.7 Hz), 43.4 (d, J = 24.1 Hz), 30.5 (d, J = 5.1 Hz), 27.7 (d, J = 26.3 Hz);

^{19}F {^{1}H} NMR (377 MHz, $CDCl_3$) δ –136.1;

IR (neat) 3297, 2944, 2865, 2124, 1454, 1363, 1102, 992, 737, 698 cm^{-1};

HRMS (EI) Calcd. for $C_{12}H_{13}F$ ($[M]^+$): 176.1001; Found: 176.1107.

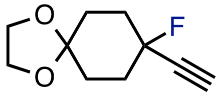

8-ethynyl-8-fluoro-1,4-dioxaspiro[4.5]decane (5b). Prepared according to general procedure F. The crude material was purified by silica gel chromatography (hexanes/ethyl acetate = 10:1) to afford the title compound as a light yellow oil.

First run: 70 mg (76% yield). Second run: 71 mg (77% yield);

^{1}H NMR (400 MHz, $CDCl_3$) δ 3.93 (s, 4H), 2.64 (d, J = 5.0 Hz, 1H), 2.21 – 1.95 (m, 4H), 1.90 – 1.69 (m, 4H);

^{13}C NMR (101 MHz, $CDCl_3$) δ 107.3, 88.1(d, J = 169.4 Hz), 82.8(d, J = 29.4 Hz), 74.6 (d, J = 8.9 Hz), 64.3, 34.8 (d, J = 23.8 Hz), 30.6 (d, J = 4.5 Hz);

^{19}F {^{1}H} NMR (377 MHz, $CDCl_3$) δ –146.7;

IR (neat) 3295, 2958, 2885, 2119, 1376, 1163, 1107, 1031, 960, 927, 661 cm^{-1};

HRMS (EI) Calcd. for $C_{10}H_{12}O_2$ ($[M-HF]^+$): 164.0837 ; Found: 164.0843.

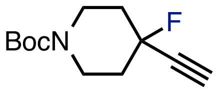

***tert*-Butyl 4-ethynyl-4-fluoropiperidine-1-carboxylate (5c).** Prepared according to general procedure F. The crude material was purified by silica gel chromatography (hexanes/ethyl acetate = 10:1) to afford the title compound as a light yellow oil.

First run: 65 mg (57% yield). Second run: 70 mg (62% yield);

^{1}H NMR (400 MHz, $CDCl_3$) δ 3.61 – 3.39 (m, 4H), 2.70 (d, *J* = 5.3 Hz, 1H), 1.93 (app. dt, *J* = 14.5, 5.4 Hz, 4H), 1.43 (s, 9H);

^{13}C NMR (101 MHz, $CDCl_3$) δ 154.4, 87.5 (d, *J* = 170.7 Hz), 82.0 (d, *J* = 29.6 Hz), 79.9, 75.6 (d, *J* = 9.6 Hz), 40.0, 36.6 (d, *J* = 22.4 Hz), 28.3;

^{19}F {^{1}H} NMR (377 MHz, $CDCl_3$) δ –143.4;

IR (neat) 3302, 2973, 2119, 1686, 1419, 1245, 1148, 1046, 914, 732 cm^{-1};

HRMS (EI) Calcd. for $C_{12}H_{18}FNO_2$ ($[M]^+$): 227.1322; Found: 227.1316.

7. Mechanistic Studies (Equations 6–8)

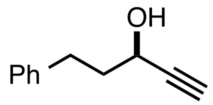

(*R*)-5-Phenyl-1-pentyn-3-ol was prepared by analogy to the literature protocol for enzymatic kinetic resolution of propargylic alcohols.[26] A 250 mL round-bottomed flask was charged with 5-phenyl-1-pentyn-3-ol (2.48 g, 15.5 mmol) and a stir bar, and toluene (100 mL) was added. *Candida antarctica* lipase (Novozyme 435; 100 mg) was added, followed by vinyl acetate (1.43 g, 15.5 mmol). The reaction mixture was stirred for 16 h at room temperature, filtered through a plug of Celite, rinsing with CH_2Cl_2 (50 mL), and concentrated under reduced pressure. The crude residue was purified by silica gel chromatography (gradient elution: 5% 7% 10% EtOAc in Hexanes) to give the product as a colourless oil (974 mg, 39%). All spectral data were in agreement with the racemic material described previously.

HPLC conditions: Chiracel IC column, 95% Hexane, 5% *i*-PrOH; 35 °C; 1.0 mL/min; 210 nm; t_R (minor) = 9.06 min; t_R (major) = 9.92 min; <1 : >99 er;

$[\alpha]^{27}_D$ = –21.4 ($CHCl_3$, *c* = 1.0).

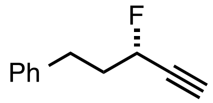

(*S*)-(3-Fluoropent-4-yn-1-yl)benzene, (*S*)-2a, was prepared by analogy to the literature protocol for dehydrofluorination of propargylic alcohols, with a minor adjustment to the rate of addition of the reagent.[27] A 25 mL round-bottomed flask equipped with a stir bar was dried under vacuum using a heat gun and cooled to room temperature. The flask was charged with (*R*)-5-Phenyl-1-pentyn-3-ol (320 mg, 2.0 mmol), a septum was fitted and the flask was evacuated and back-filled with nitrogen three times. CH_2Cl_2 (8 mL) was added using a syringe and the solution was stirred at –50 °C using a cryo-cool to maintain reaction temperature. Meanwhile, an oven-dired 4 mL vial fitted with a stir bar was charged with diethylaminosulfur trifluoride (DAST; 262 μL, 2.0 mmol), dissolved in CH_2Cl_2 (2 mL), and stirred for 5 mins. The DAST solution was withdrawn into a 3 mL syringe and added over a period of 5 minutes to the solution of the alcohol. Dropwise addition was crucial and the solution was added down the side of the round-bottomed flask to prohibit significant heat fluctuations. After 1.5 h, the septum was removed and a saturated aqueous solution of Na_2CO_3 (10 mL) was added and the mixture was allowed to warm to room temperature. The organic solution was separated and the aqueous phase was extracted using CH_2Cl_2 (3 x 30 mL), dried (Na_2SO_4) and concentration under reduced pressure. The crude

---

[26] Raminelli, C.; Comasseto, J. V.; Andrade, L. H.; Porto, A. L. M. *Tetrahedron: Asymmetry* **2004**, *15*, 3117–3122.

[27] Manthati, V. L.; Krishna Murthy, A. S.; Caijo, F.; Drouin, D.; Lesot, P.; Grée, D.; Grée, R. *Tetrahedron: Asymmetry* **2006**, *17*, 2306–2310.

residue was purified by silica gel chromatography (hexanes) to afford the product as a colourless oil (135 mg, 42%). All spectral data were in agreement with the racemic material described previously.

HPLC conditions: Chiracel IC column, 100% Hexane; 35 °C; 1.0 mL/min; 210 nm; t_R (minor) = 8.30 min; t_R (major) = 9.01 min; 3 : 97 er;

$[\alpha]^{27}_D$ = +13.5 ($CHCl_3$, *c* = 1.0).

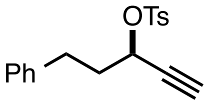

(*R*)-5-Phenylpent-1-yn-3-yl 4-methylbenzenesulfonate, (*R*)-1a-OTs, was synthesized according to general procedure C for preparation of propargylic tosylate utilizing (*R*)-5-Phenyl-1-pentyn-3-ol (320 mg, 2 mmol), TsCl (420 mg, 2.2 mmol) and KOH (829 mg, 4.8 mmol). The crude material was purified by silica gel chromatography (hexanes/ethyl acetate = 5:1) to afford the title compound as a colorless oil (593 mg, 94% yield).

HPLC conditions: Chiracel IC column, 90% Hexane, 10% *i*-PrOH; 35 °C; 1.0 mL/min; 210 nm; t_R (minor) = 14.10 min; t_R (major) = 15.49 min; 1 : > 99 er;

$[\alpha]^{27}_D$ = +22.3 ($CHCl_3$, *c* = 1.0).

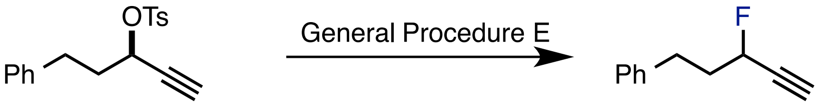

(*R*)-**1a-OTs**
>99:1 er

(±)-**2a**
50:50 er

The reaction was performed according to general procedure D utilizing (*R*)-5-Phenylpent-1-yn-3-yl 4-methylbenzenesulfonate (94.2 mg, 0.3 mmol), (IPr)CuCl (14.6 mg, 0.03 mmol) and Et_3N•3HF (0.15 mL, 0.9 mmol). The crude material was purified by silica gel chromatography (hexanes) to afford the title compound as a colorless oil (42 mg, 94% yield). 1.4% ee, HPLC conditions: Chiracel IC column, 100% Hexane; 35 °C; 1.0 mL/min; 210 nm; t_R (minor) = 7.72 min; t_R (major) = 8.71 min.

Repeating the transformation described above but for just 10 h: The reaction mixture was filtered through a pad of silica gel (a pipette with about 5 cm silica gel) and washed with ethyl acetate (10 mL). The filtrate was concentrated under reduced pressure. The residue was dissolved in $CDCl_3$ and CH_2Br_2 (0.1 mmol) was added as internal standard for ^1H NMR analysis to determine the reaction conversion (52%). Then the remaining starting material and product were separated by silica gel chromatography (hexanes).

For product: HPLC conditions: Chiracel IC column, 100% Hexane; 35 °C; 1.0 mL/min; 210 nm; t_R (minor) = 7.81 min; t_R (major) = 8.78 min; ca. 50 : 50 er.

For remaining starting material: HPLC conditions: Chiracel IC column, 90% Hexane, 10% *i*-PrOH; 35 °C; 1.0 mL/min; 210 nm; t_R (minor) = 14.66 min; t_R (major) = 16.17 min; 3.5 : 96.5 er.

8. Derivations of Propargylic Fluoride 2a (Scheme 2)

Procedure for Sonogashira coupling reaction[28]

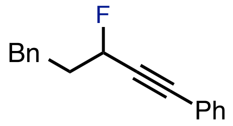

(3-Fluoropent-1-yne-1,5-diyl)dibenzene (7). Propargylic fluoride (81 mg, 0.5 mmol), iodobenzene (81.6 mg, 0.4 mmol), $Pd(PPh_3)_4$ (58 mg, 0.05 mmol) and CuI (10 mg, 0.05 mmol) were added in turn to a 4 mL vial with a 1 cm stir bar. The vial was capped with a PTFE-lined cap and then it was evacuated and refilled with N_2 three times. Triethylamine (1.0 mL) was added by syringe at room temperature and the resulting solution was then stirred at 60 °C for 8 hours. After cooled to room temperature, the crude mixture was passed through a short pad of silica gel (a pipette with about 5 cm silica gel), washed with ethyl acetate (20 mL) and concentrated. The residue was purified by silica gel chromatography (hexane) to afford the desired product (100 mg, 84%) as a colorless oil.

^{1}H NMR (400 MHz, $CDCl_3$) δ 7.52 (dd, *J* = 7.5, 2.1 Hz, 2H), 7.44 – 7.33 (m, 5H), 7.32 – 7.25 (m, 3H), 5.37 (dt, *J* = 48.5, 6.3 Hz, 1H), 3.04 – 2.85 (m, 2H), 2.47 – 2.19 (m, 2H);

^{13}C NMR (101 MHz, $CDCl_3$) δ 140.6, 131.8, 128.9, 128.5 (2C), 128.3, 126.2, 121.8 (d, *J* = 3.1 Hz), 88.3 (d, *J* = 10.4 Hz), 85.3 (d, *J* = 25.7 Hz), 82.3 (d, *J* = 165.9 Hz), 37.6 (d, *J* = 22.6 Hz), 30.8 (d, *J* = 5.0 Hz);

^{19}F {^{1}H} NMR (377 MHz, $CDCl_3$) δ –173.5;

IR (neat) 3062, 3027, 2956, 2928, 2232, 1490, 1348, 1031, 909, 1041, 754, 691 cm^{-1};

HRMS (EI) Calcd. for $C_{17}H_{15}F([M]^+)$: 238.1158; Found: 238.1160.

Procedure for synthesis of allene[29]

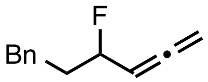

(3-Fluorohexa-4,5-dien-1-yl)benzene (8). $(HCHO)_n$ (37.5 mg, 1.25 mmol), CuI (47.5 mg, 0.25 mmol) were added in turn to a 10 mL schlenk flask with a 1 cm stir bar. The flask was capped with a rubber septum and then it was evacuated and refilled with N_2 three times. 1,4-Dioxane (2.0 mL) was added to the flask by a syringe at room temperature followed by $(c\text{-Hex})_2NH$ (0.18 mL, 0.9 mmol) and propargylic fluoride (81.0 mg, 0.5 mmol). The resulting mixture was then placed in a pre-heated oil bath at 110 °C and stirred for 24 h. After cooling to room temperature, the crude reaction mixture was concentrated, filtered through a short pad of silica gel (a pipette with about 5 cm silica gel) and washed with EtOAc (15 mL). The filtrate was concentrated under reduced pressure and diluted by Et_2O (20 mL) and 5% HCl (20 mL). The organic phase was separated and the water

[28] Nishimura, T.; Guo, X.-X.; Uchiyama, N.; Katoh, T. Hayashi, T. *J. Am. Chem. Soc.* **2008**, *130*, 1576–1577.
[29] Kuang, J.; Ma, S. *J. Org. Chem.* **2009**, *74*, 1763–1765.

phase was extracted with Et_2O (3 x 20 mL). The combined organic phases were washed with brine, dried over $MgSO_4$, filtered and then concentrated. The crude oil was purified by silica gel chromatography (hexane) to afford the desired product (62 mg, 70% yield) as a colorless oil.

^{1}H NMR (400 MHz, $CDCl_3$) δ 7.39 – 7.32 (m, 2H), 7.29 – 7.24 (m, 3H), 5.43 – 5.35 (m, 1H), 5.11 – 4.88 (m, 3H), 2.91 – 2.72 (m, 2H), 2.25 – 1.95 (m, 2H);

^{13}C NMR (101 MHz, $CDCl_3$) δ 208.9 (d, *J* = 10.9 Hz), 141.2, 128.5 (2C), 126.1, 90.8 (d, *J* = 24.3 Hz), 90.3 (d, *J* = 168.9 Hz), 77.3, 37.0 (d, *J* = 22.3 Hz), 31.1 (d, *J* = 4.7 Hz);

^{19}F {^{1}H} NMR (377 MHz, $CDCl_3$) δ –170.5;

IR (neat) 3028, 2951, 1956, 1497, 1455, 1020, 849, 745, 698 cm^{-1}.

Procedure for hydrogenation of 2a[30]

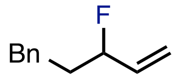

(3-Fluoropent-4-en-1-yl)benzene (9). Propargylic fluoride (81 mg, 0.5 mmol) and EtOAc (3 mL) were added to a 25 mL flask with a 1.5 cm stir bar. Next, Lindlar catalyst (25 mg) and quinoline (60μL, 0.5 mmol) were added to the above solution at room temperature under air. While the mixture was stirred, the flask was evacuated and refilled with H_2 from a balloon. After being stirred under H_2 atmosphere at room temperature for 24 h, the mixture was filtered through a pad of silica gel (a pipette with about 5 cm silica gel). The filtrate was concentrated under reduced pressure and purified by silica gel chromatography (hexane) to afford the product (78 mg, 95%) as a colorless oil.

^{1}H NMR (400 MHz, $CDCl_3$) δ 7.41 – 7.33 (m, 2H), 7.31 – 7.24 (m, 3H), 6.08 – 5.89 (m, 1H), 5.46 – 5.35 (m, 1H), 5.35 – 5.26 (m, 1H), 5.07 – 4.86 (m, 1H), 2.93 – 2.72 (m, 2H), 2.21 – 1.90 (m, 2H);

^{13}C NMR (101 MHz, $CDCl_3$) δ 141.2, 136.4 (d, *J* = 19.3 Hz), 128.5 (2C), 126.0, 117.1 (d, *J* = 11.5 Hz), 92.6 (d, *J* = 166.3 Hz), 36.9 (d, *J* = 22.2 Hz), 30.9 (d, *J* = 4.4 Hz);

^{19}F {^{1}H} NMR (377 MHz, $CDCl_3$) δ –178.8;

IR (neat) 3028, 2949, 1603, 1497, 1454, 1030, 989, 931, 747, 700 cm^{-1};

HRMS (EI) Calcd. for $C_{11}H_{13}F([M]^+)$: 164.1001; Found: 164.1008.

Procedure for Hydration of 2a[31]

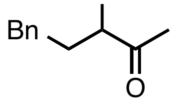

[30] He, Y.; Yang H.; Yao, Z. *Tetrahedron* **2002**, *58*, 8805–8810.
[31] Thuong, M. B. T.; Mann, A.; Wagner, A. *Chem. Commun.* **2012**, *48*, 434–436.

3-Fluoro-5-phenylpentan-2-one (10). Propargylic fluoride (81 mg, 0.5 mmol) was added to a 4 mL vial with a 1 cm stir bar. In air, MeOH (0.75 mL) was added to the vial, followed by water (75 µL) and $AgSbF_6$ (17.2 mg, 0.05 mmol) at room temperature. The vial was capped with a PTFE-lined cap and stirred at 75 °C for 24 hours. Then the mixture was concentrated and Et_2O (50 mL) was added. The organic phase was washed with aqueous ammonia (10 mL), dried over Na_2SO_4 and concentrated. The residue was purified by silica gel chromatography (hexane/EtOAc = 15:1) to afford the desired product (52 mg, 58% yield) as pale yellow oil.

^{1}H NMR (400 MHz, $CDCl_3$) δ 7.33 – 7.29 (m, 2H), 7.27 – 7.23 (m, 3H), 4.71 (ddd, J = 50.1, 8.2, 4.4 Hz, 1H), 2.88 – 2.71 (m, 2H), 2.27 (d, J = 4.6 Hz, 3H), 2.21 – 2.03 (m, 2H);

^{13}C NMR (101 MHz, $CDCl_3$) δ 208.1(d, J = 25.4 Hz), 140.1, 128.6 (2C), 126.3, 94.9 (d, J = 183.1 Hz), 33.3 (d, J = 20.6 Hz), 30.5 (d, J = 2.7 Hz), 25.8;

^{19}F {^{1}H} NMR (377 MHz, $CDCl_3$) δ –190.7;

IR (neat) 3028, 2929, 1725, 1357, 1081, 1041, 914, 735, 700 cm^{-1};

HRMS (CI) Calcd. for $C_{11}H_{17}NOF$ ($[M+NH_4]^+$): 198.1294; Found: 198.1291.

Procedure for the click reaction[32]

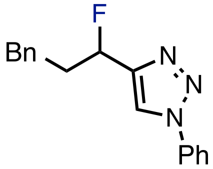

4-(1-Fluoro-3-phenylpropyl)-1-phenyl-1H-1,2,3-triazole (11). Sodium ascorbate (19.8 mg, 0.1 mmol) and $CuSO_4·5H_2O$ (12.5 mg, 0.05 mmol) were added to a 4 mL via with a 1 cm stir bar. The vial was capped with a PTFE-lined cap and then it was evacuated and refilled with N_2 three times. A mixture of *t*-BuOH/H_2O (V/V = 1:1, 3.0 mL) was added to the vial and then progargylic fluoride (81 mg, 0.5 mmol), azidobenzene (1.1 mL, 0.5 M in 2-Me-THF, 0.55 mmol) were added sequentially at room temperature. The above mixture was stirred at ambient temperature for 18 h, upon which the mixture was diluted with NH_4Cl (10 mL), extracted with EtOAc (3 x 15 mL). The combined organic phases were washed with brine, dried over $MgSO_4$, filtered and then concentrated. The crude oil was purified by silica gel chromatography (hexane/EtOAc = 10:1) to afford the desired product (102 mg, 82% yield) as a pale yellow oil.

^{1}H NMR (400 MHz, $CDCl_3$) δ 8.04 (s, 1H), 7.76 (d, J = 7.7 Hz, 2H), 7.56 (t, J = 7.6 Hz, 2H), 7.48 (t, J = 7.3 Hz, 1H), 7.38 – 7.22 (m, 5H), 5.78 (ddd, J = 48.1, 8.5, 4.5 Hz, 1H), 3.00 – 2.82 (m, 2H), 2.61 – 2.35 (m, 2H);

^{13}C NMR (101 MHz, $CDCl_3$) δ 147.7 (d, J = 24.3 Hz), 140.6, 136.8, 129.7 (2C), 128.9, 128.5 (d, J = 2.7 Hz), 126.1, 120.5, 119.8, 86.8 (d, J = 165.9 Hz), 36.6 (d, J = 22.1 Hz), 30.9 (d, J = 4.5 Hz);

^{19}F {^{1}H} NMR (377 MHz, $CDCl_3$) δ –175.8;

---

[32] Jiang, H.; Falcicchio, A.; Jensen, K.L.; Paixão, M.W.; Bertelsen, S.; Jørgensen, K. A. *J. Am.Chem.Soc.* **2009**, *131*, 7153–7157.

IR (neat) 3266, 3134, 2955, 1502, 1243, 1219, 1037, 904, 860, 752, 699 cm^{-1}.

HRMS (ESI) Calcd. for $C_{17}H_{17}FN_3$([M+H]$^+$): 282.1407; Found: 282.1416.

Procedure for [2+2+2] cyclotrimerization reaction[33]

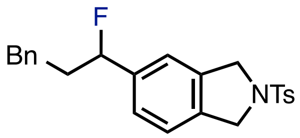

5-(1-Fluoro-3-phenylpropyl)-2-tosylisoindoline (12). Propargylic fluoride (81 mg, 0.5 mmol) and Cp*RuCl(cod) (4.8 mg, 0.0125 mmol) were added to a 4 mL vial with a 1 cm stir bar. The vial was capped with a PTFE-lined cap and then it was evacuated and refilled with N_2 three times. Then dry degassed 1,2-dichloroethane (1 mL) was added to the vial. A solution of *N*,*N*-bis(propargyl)-tosylsulfonamide (61.7 mg, 0.25 mmol) in dry degassed 1,2-dichloroethane (1 mL) was added to the above solution via a syringe pump at room temperature for 15 min. After the addition completion, the mixture was stirred at room temperature for another 15 min. Then the mixture was concentrated and the residue was purified by silica gel chromatography (hexane) to afford the desired product (75 mg, 74%) as a brown oil.

^{1}H NMR (400 MHz, $CDCl_3$) δ 7.78 (d, *J* = 8.1 Hz, 2H), 7.36 – 7.29 (m, 4H), 7.24 – 7.13 (m, 6H), 5.39 (ddd, *J* = 47.6, 8.5, 4.3 Hz, 1H), 4.62 (s, 3H), 2.83 – 2.67 (m, 2H), 2.40 (s, 3H), 2.32 – 1.99 (m, 2H);

^{13}C NMR (101 MHz, $CDCl_3$) δ 143.7, 140.8, 140.1(d, *J* = 20.0 Hz), 136.5, 136.2, 133.5, 129.8 (2C), 128.4 (2C), 127.5, 126.1, 125.2 (d, *J* = 6.5 Hz), 122.7, 119.8 (d, *J* = 6.7 Hz), 93.2 (d, *J* = 170.5 Hz), 53.5, 38.8 (d, *J* = 23.8 Hz), 31.2 (d, *J* = 4.1 Hz), 21.4;

^{19}F {^{1}H} NMR (377 MHz, $CDCl_3$) δ –175.7;

IR (neat) 3028, 2924, 1598, 1374, 1165, 1098, 910, 735, 668, 568, 548 cm^{-1};

HRMS (ESI) Calcd. for $C_{24}H_{25}NO_2SF$ ([M+H]$^+$): 410.1590; Found: 410.1591.

[33] Yamamoto, Y.; Arakawa, T.; Ogawa, R.; Itoh, K. *J. Am. Chem. Soc.* **2003**, *125*, 12143–12160.

9. HPLC Data for Enantioenriched Substrates

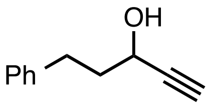

5-Phenyl-1-pentyn-3-ol

```
Data File C:\CHEM32\1\DATA\DEF_LC 2014-12-04 08-54-07\CJC-70-ROH4.D
Sample Name: ROH4

    =====================================================================
    Acq. Operator   : SYSTEM                          Seq. Line :  11
                                                       Location : Vial 11
    Injection Date  : 04/12/2014 11:28:35                    Inj :   1
    Acq. Method     : IC_5%_20.M
    Analysis Method : C:\CHEM32\1\METHODS\IF_2%_10.M
    Last changed    : 07/07/2015 18:44:04 by SYSTEM
                      (modified after loading)
    Additional Info : Peak(s) manually integrated
```

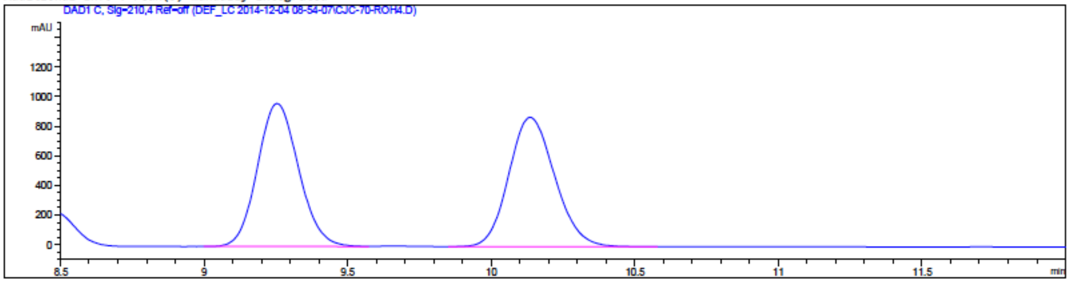


```
Data File C:\CHEM32\1\DATA\DEF_LC 2014-12-04 08-54-07\CJC-70-ROH4.D
Sample Name: ROH4

    Signal 1: DAD1 C, Sig=210,4 Ref=off

    Peak RetTime Type  Width     Area      Height     Area
      #   [min]        [min]   [mAU*s]     [mAU]       %
    ----|-------|----|-------|----------|----------|--------|
       1   9.253 BV    0.1543 9617.16992  965.02753  49.8223
       2  10.135 VB    0.1735 9685.78809  872.35760  50.1777

    Totals :                  1.93030e4  1837.38513

    =====================================================================
                              *** End of Report ***
```

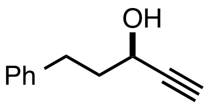

(*R*)-5-Phenyl-1-pentyn-3-ol

Data File C:\CHEM32\1\DATA\DEF_LC 2014-12-05 07-41-17\CJC-70-1.D
Sample Name: CJC-70-1

```
=====================================================================
Acq. Operator   : SYSTEM                        Seq. Line :  15
                                                 Location : Vial 14
Injection Date  : 05/12/2014 11:20:29                 Inj :   1
Acq. Method     : IC_5%_20.M
Analysis Method : C:\CHEM32\1\METHODS\IF_2%_10.M
Last changed    : 07/07/2015 18:48:55 by SYSTEM
                  (modified after loading)
Additional Info : Peak(s) manually integrated
```

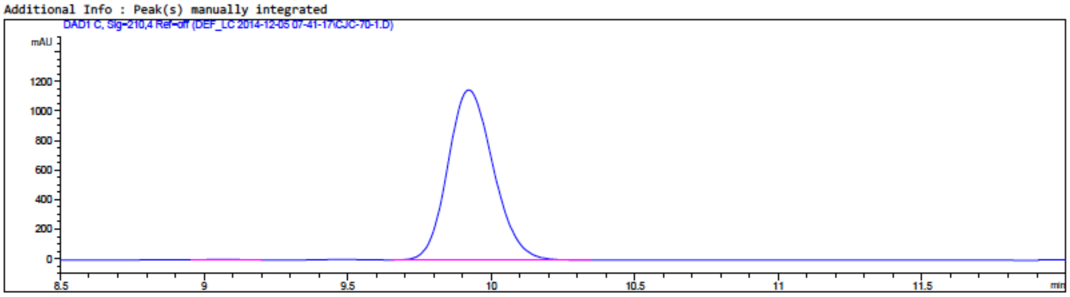

Data File C:\CHEM32\1\DATA\DEF_LC 2014-12-05 07-41-17\CJC-70-1.D
Sample Name: CJC-70-1

Signal 1: DAD1 C, Sig=210,4 Ref=off

Peak #	RetTime [min]	Type	Width [min]	Area [mAU*s]	Height [mAU]	Area %
1	9.064	MM	0.1507	32.68003	3.61542	0.2573
2	9.919	MM	0.1836	1.26672e4	1149.65955	99.7427

Totals : 1.26999e4 1153.27497

===
*** End of Report ***

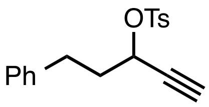

5-Phenylpent-1-yn-3-yl 4-methylbenzenesulfonate (1a-OTs)

```
Data File C:\CHEM32\1\DATA\LJC-4-74-IC 2015-03-16 16-40-58\LJC-4-74-RAC.D
Sample Name: ljc-4-74-rac

    =====================================================================
    Acq. Operator   : SYSTEM                        Seq. Line :   1
                                                     Location : Vial 3
    Injection Date  : 16/03/2015 16:42:36                 Inj :   1
    Acq. Method     : IC_10%_30.M
    Analysis Method : C:\CHEM32\1\METHODS\IF_2%_10.M
    Last changed    : 07/07/2015 18:58:50 by SYSTEM
                      (modified after loading)
    Additional Info : Peak(s) manually integrated
```

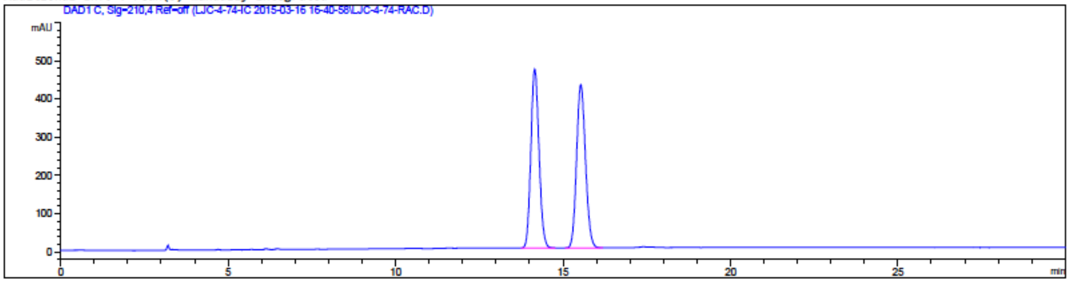


```
Data File C:\CHEM32\1\DATA\LJC-4-74-IC 2015-03-16 16-40-58\LJC-4-74-RAC.D
Sample Name: ljc-4-74-rac

    Signal 1: DAD1 C, Sig=210,4 Ref=off
```

Peak #	RetTime [min]	Type	Width [min]	Area [mAU*s]	Height [mAU]	Area %
1	14.153	BB	0.2637	7928.90430	467.84317	50.0176
2	15.528	BB	0.2885	7923.31201	427.00876	49.9824

Totals : 1.58522e4 894.85193

===

*** End of Report ***

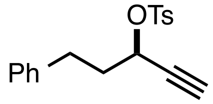

(*R*)-5-Phenylpent-1-yn-3-yl 4-methylbenzenesulfonate, (*R*)-1a-OTs

Data File C:\CHEM32\1\DATA\LJC-4-74-IC-2 2015-03-17 09-39-40\LJC-4-74-OPT.D
Sample Name: ljc-4-74-opt

===

Acq. Operator : SYSTEM Seq. Line : 3
Location : Vial 4
Injection Date : 17/03/2015 10:23:57 Inj : 1
Acq. Method : IC_10%_30.M
Analysis Method : C:\CHEM32\1\METHODS\IF_2%_10.M
Last changed : 07/07/2015 19:03:10 by SYSTEM
(modified after loading)
Additional Info : Peak(s) manually integrated

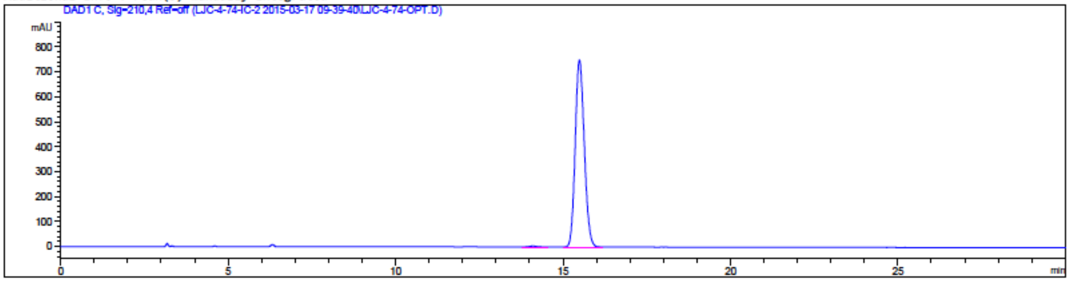

Data File C:\CHEM32\1\DATA\LJC-4-74-IC-2 2015-03-17 09-39-40\LJC-4-74-OPT.D
Sample Name: ljc-4-74-opt

Signal 1: DAD1 C, Sig=210,4 Ref=off

Peak #	RetTime [min]	Type	Width [min]	Area [mAU*s]	Height [mAU]	Area %
1	14.099	BB	0.2604	77.48779	4.51176	0.5426
2	15.490	BB	0.2947	1.42041e4	750.84528	99.4574

Totals : 1.42816e4 755.35704

===
*** End of Report ***

Agilent 1260 07/07/2015 19:03:17 SYSTEM Page 2 of 2

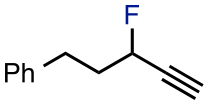

(3-Fluoropent-4-yn-1-yl)benzene (2a).

Data File C:\CHEM32\1\DATA\CJC-71-1 2014-12-08 13-32-38\CJC-71-RAC-5.D
Sample Name: CJC-71-RAC5

===
Acq. Operator : SYSTEM Seq. Line : 7
Location : Vial 2
Injection Date : 08/12/2014 14:51:36 Inj : 1
Acq. Method : IC_0%_15.M
Analysis Method : C:\CHEM32\1\METHODS\IF_2%_10.M
Last changed : 07/07/2015 18:22:57 by SYSTEM
(modified after loading)
Additional Info : Peak(s) manually integrated

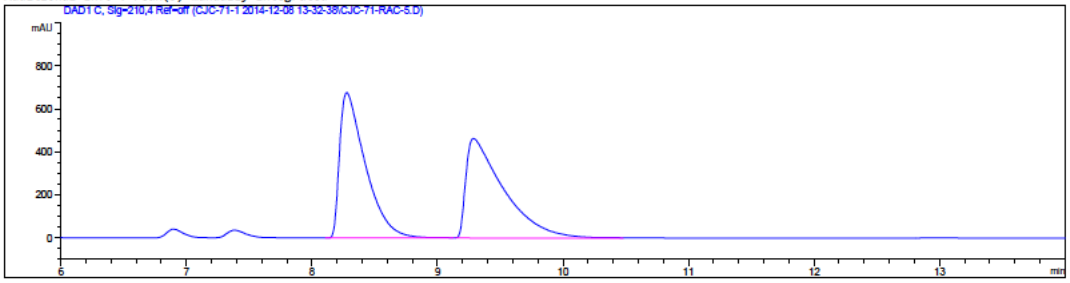

Data File C:\CHEM32\1\DATA\CJC-71-1 2014-12-08 13-32-38\CJC-71-RAC-5.D
Sample Name: CJC-71-RAC5

Signal 1: DAD1 C, Sig=210,4 Ref=off

Peak #	RetTime [min]	Type	Width [min]	Area [mAU*s]	Height [mAU]	Area %
1	8.280	BV	0.2090	9674.55273	675.94666	50.0271
2	9.290	MM	0.3486	9664.05469	462.07916	49.9729
Totals :				1.93386e4	1138.02582	

===
*** End of Report ***

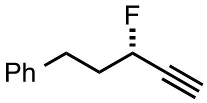

(*S*)-(3-Fluoropent-4-yn-1-yl)benzene, (*S*)-2a

Data File C:\CHEM32\1\DATA\CJC-71-1 2014-12-08 13-32-38\CJC-71-4.D
Sample Name: CJC-71-4

===
Acq. Operator : SYSTEM Seq. Line : 5
Location : Vial 31
Injection Date : 08/12/2014 14:23:58 Inj : 1
Acq. Method : IC_0%_15.M
Analysis Method : C:\CHEM32\1\METHODS\IF_2%_10.M
Last changed : 07/07/2015 18:13:27 by SYSTEM
(modified after loading)
Additional Info : Peak(s) manually integrated

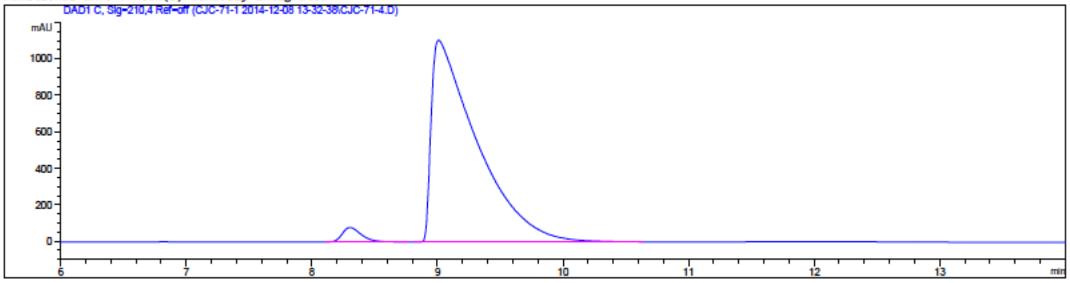

Data File C:\CHEM32\1\DATA\CJC-71-1 2014-12-08 13-32-38\CJC-71-4.D
Sample Name: CJC-71-4

Signal 1: DAD1 C, Sig=210,4 Ref=off

Peak #	RetTime [min]	Type	Width [min]	Area [mAU*s]	Height [mAU]	Area %
1	8.301	MM	0.1755	837.89923	79.55779	2.9161
2	9.007	BB	0.3565	2.78955e4	1105.12329	97.0839

Totals : 2.87334e4 1184.68108

===
*** End of Report ***

10. ^{1}H and ^{13}C NMR Spectra for Unknown Compounds

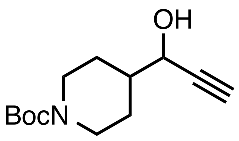

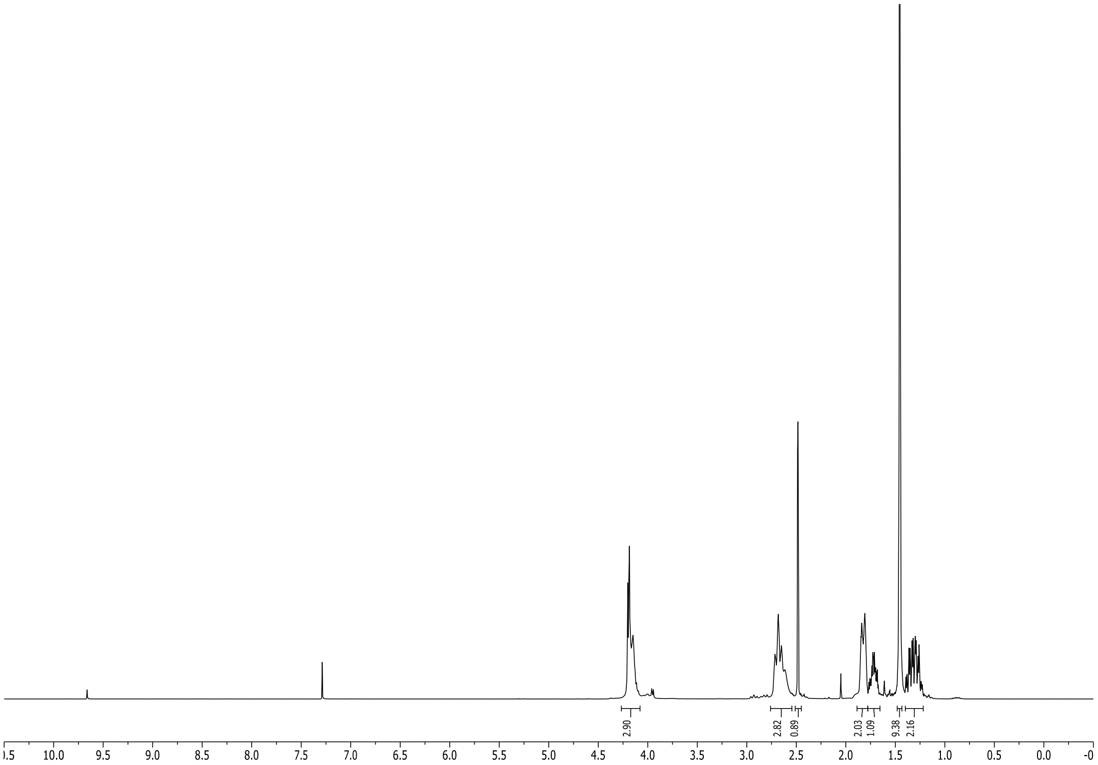

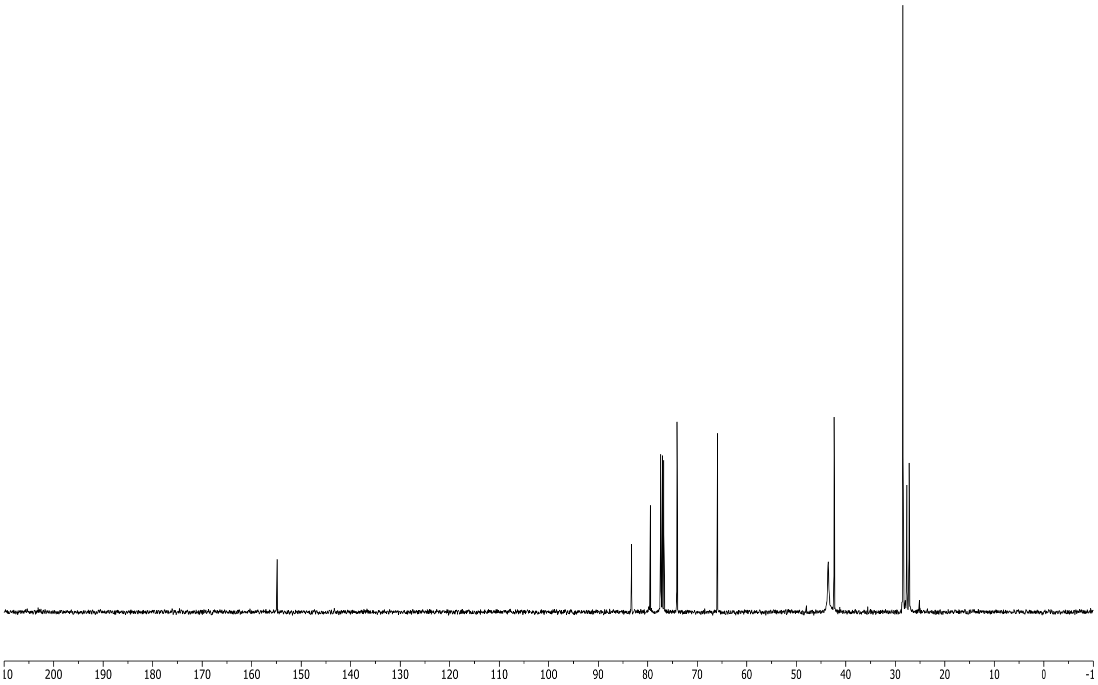

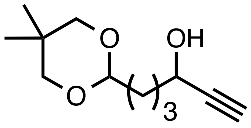
OH
O
O
3

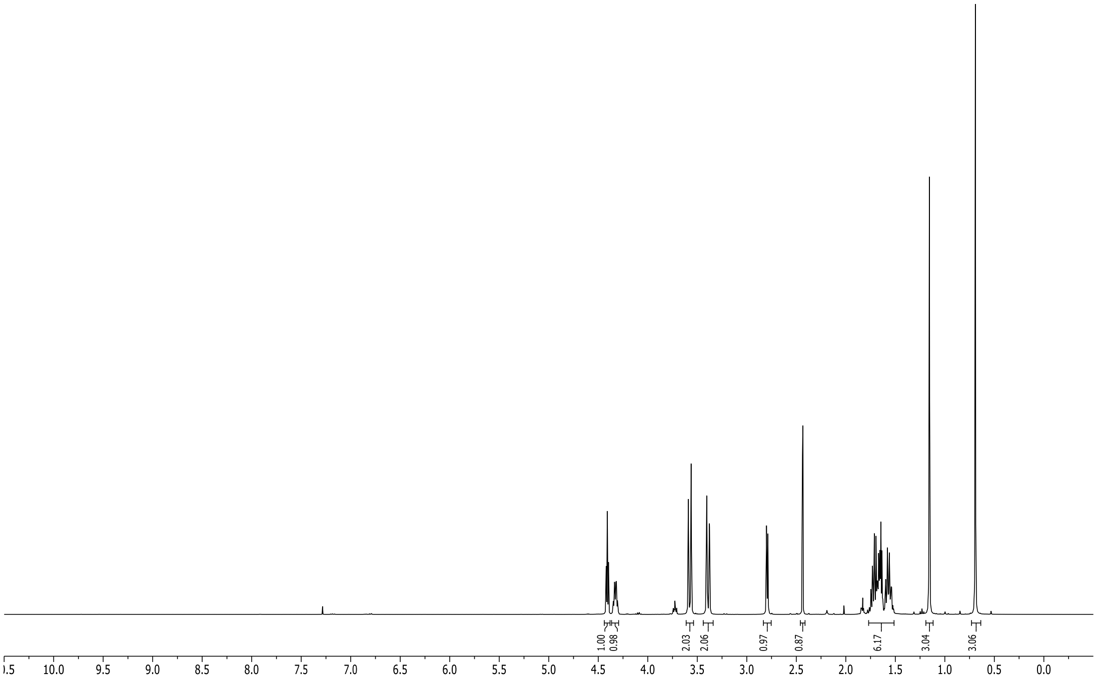
1.00
0.98
2.03
2.06
0.97
0.87
6.17
3.04
3.06
10.0 9.5 9.0 8.5 8.0 7.5 7.0 6.5 6.0 5.5 5.0 4.5 4.0 3.5 3.0 2.5 2.0 1.5 1.0 0.5 0.0

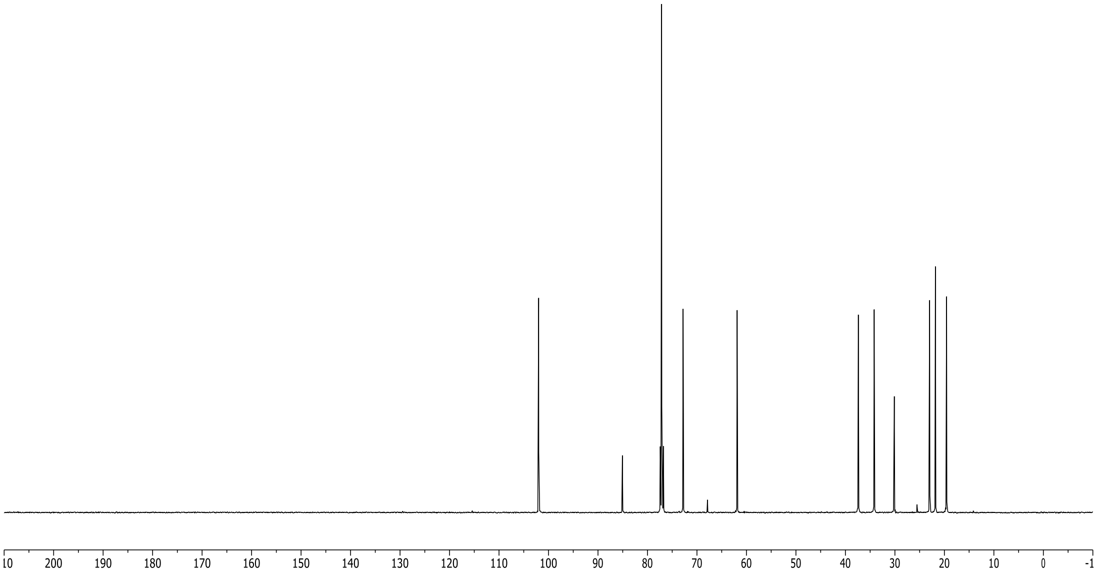
200 190 180 170 160 150 140 130 120 110 100 90 80 70 60 50 40 30 20 10 0

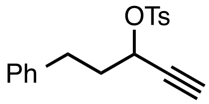
OTs
Ph

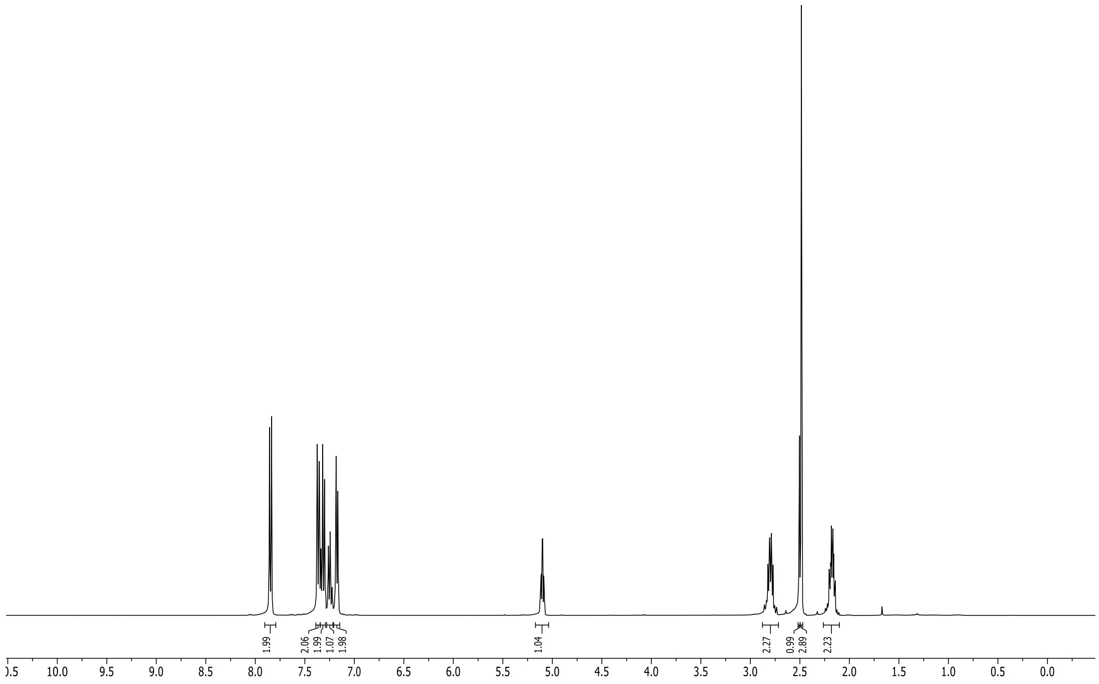
1.99
2.06
1.99
1.07
1.98
1.04
2.27
0.99
2.89
2.23
10.0 9.5 9.0 8.5 8.0 7.5 7.0 6.5 6.0 5.5 5.0 4.5 4.0 3.5 3.0 2.5 2.0 1.5 1.0 0.5 0.0

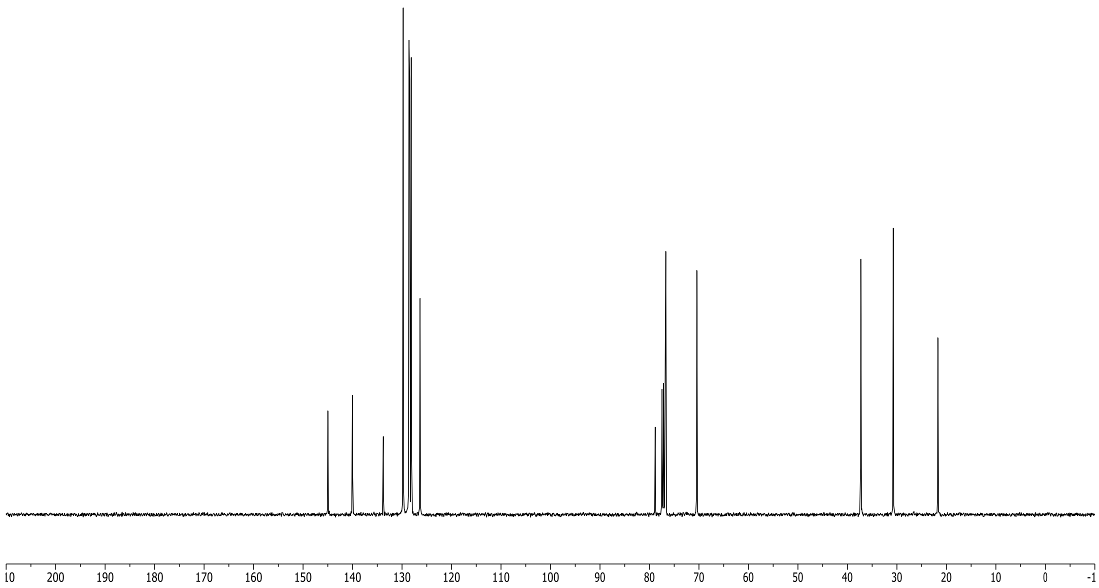
200 190 180 170 160 150 140 130 120 110 100 90 80 70 60 50 40 30 20 10 0

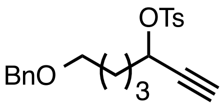
OTs
BnO
3

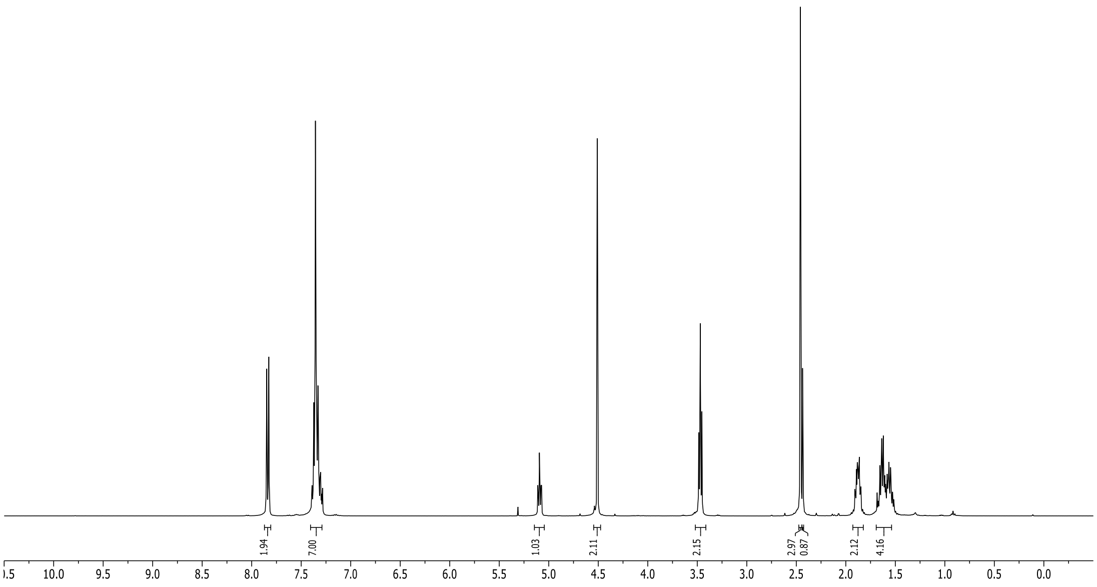
1.94
7.00
1.03
2.11
2.15
2.97
0.87
2.12
4.16
10.0 9.5 9.0 8.5 8.0 7.5 7.0 6.5 6.0 5.5 5.0 4.5 4.0 3.5 3.0 2.5 2.0 1.5 1.0 0.5 0.0

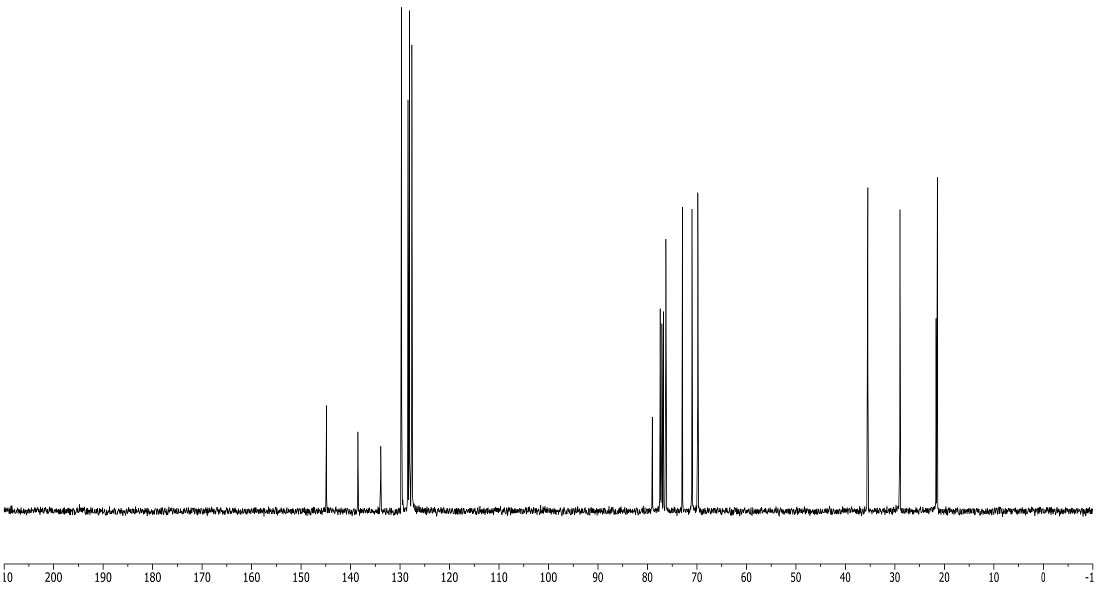
200 190 180 170 160 150 140 130 120 110 100 90 80 70 60 50 40 30 20 10 0

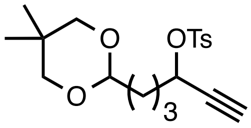
OTs
O
O
3

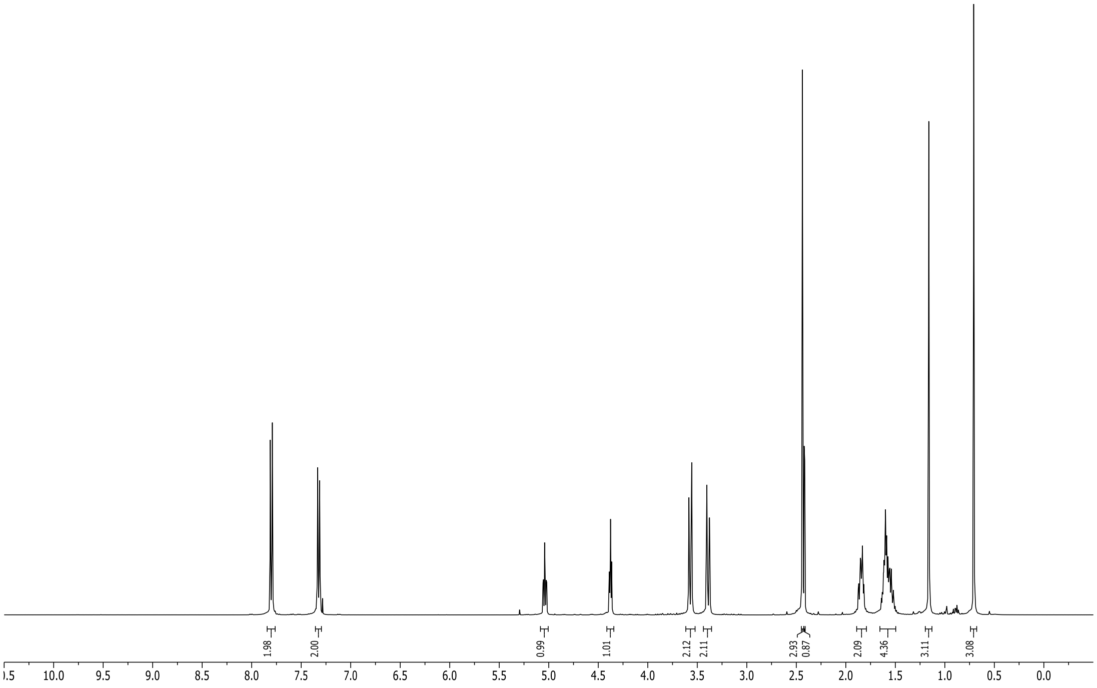

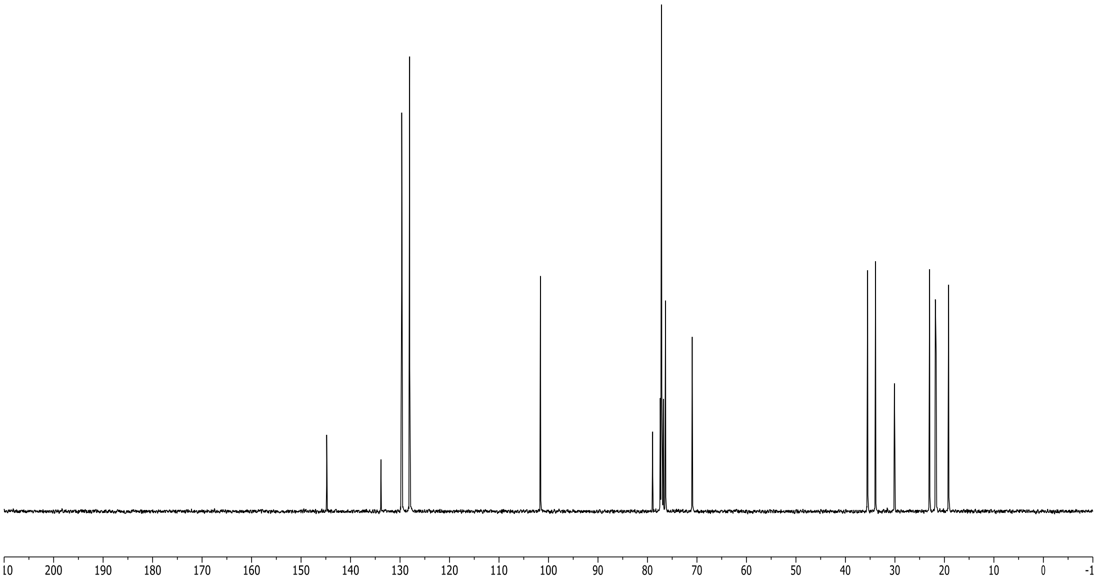

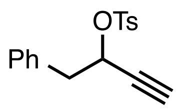
OTs
Ph

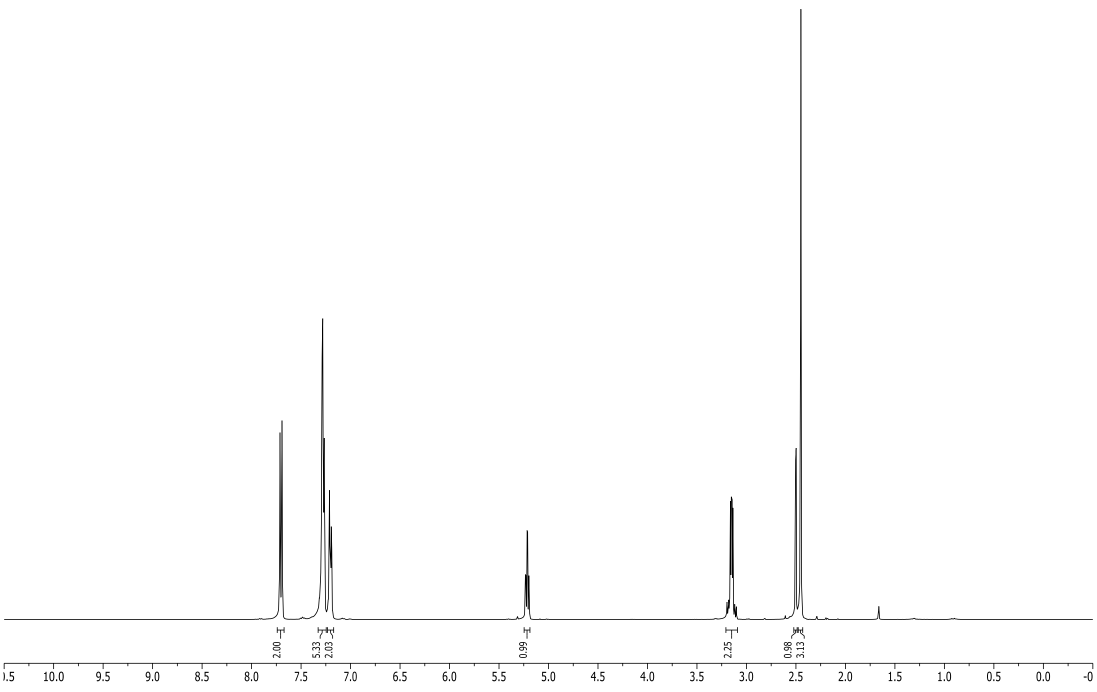

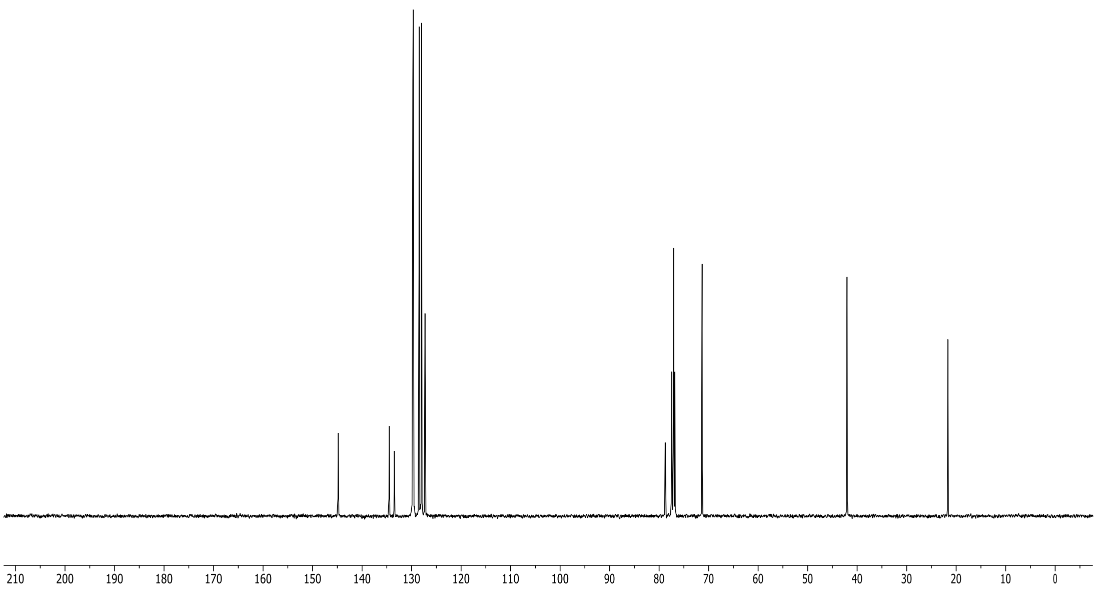

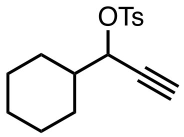
OTs

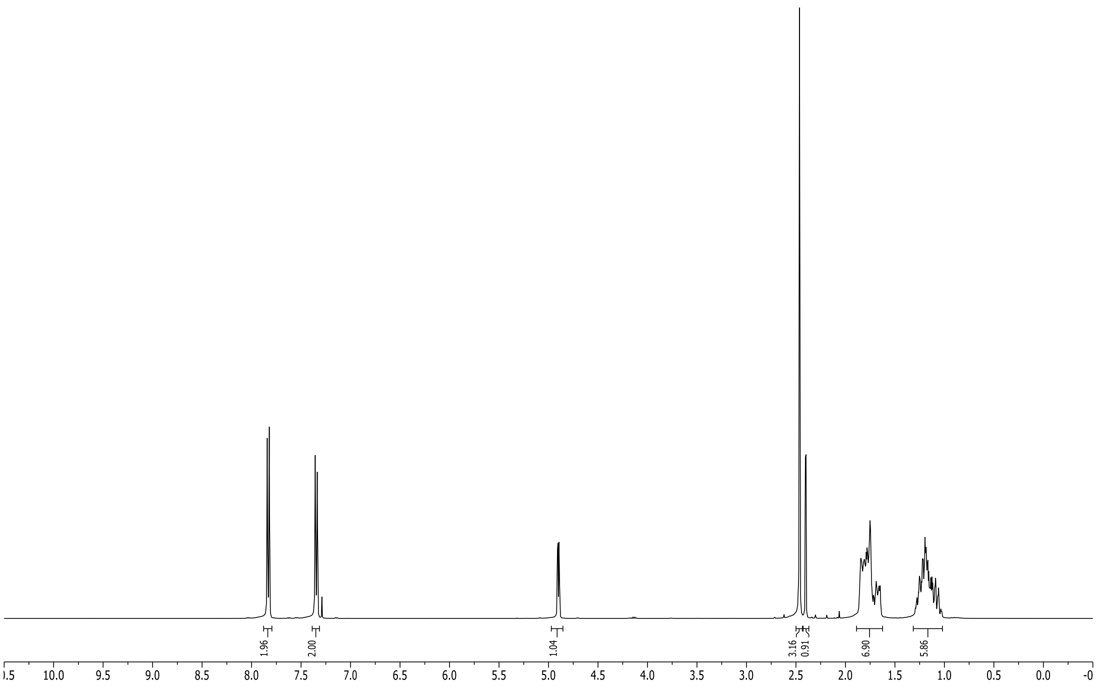

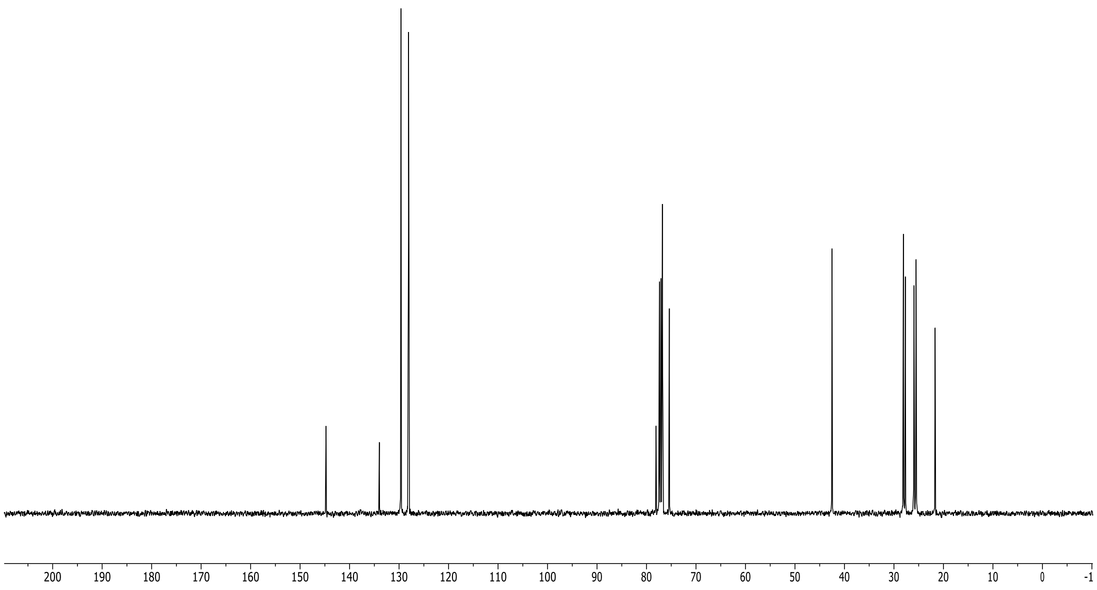

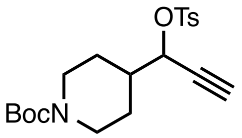
OTs
BocN

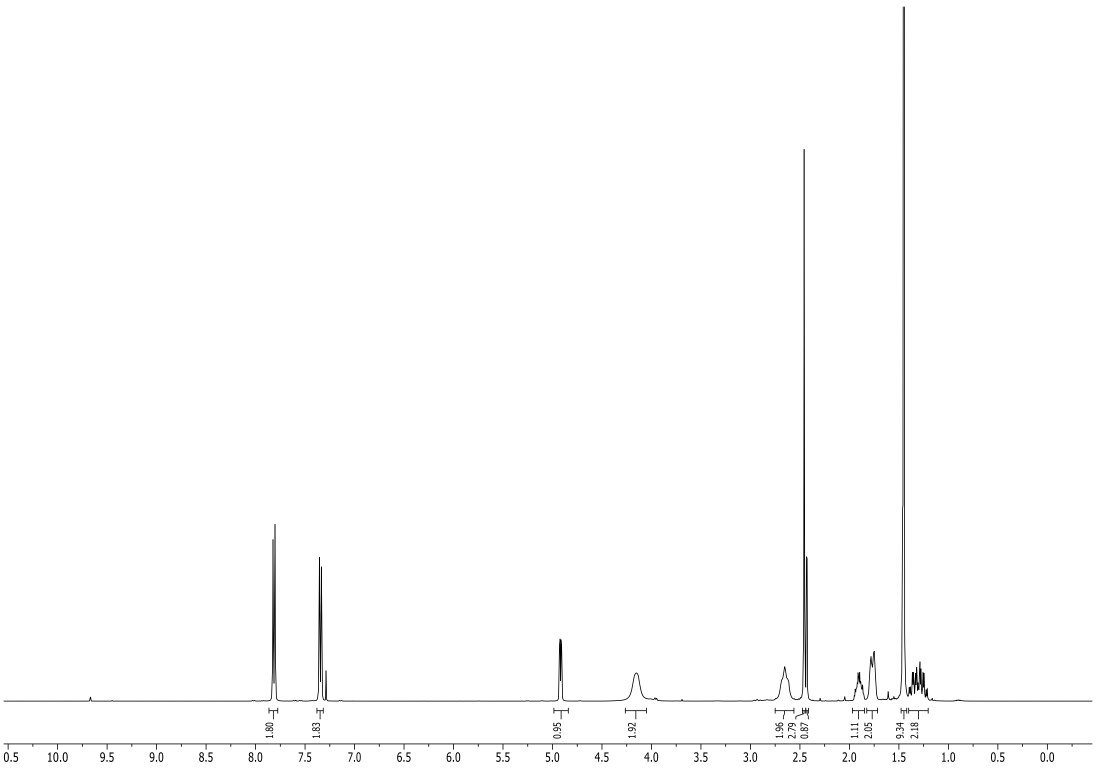

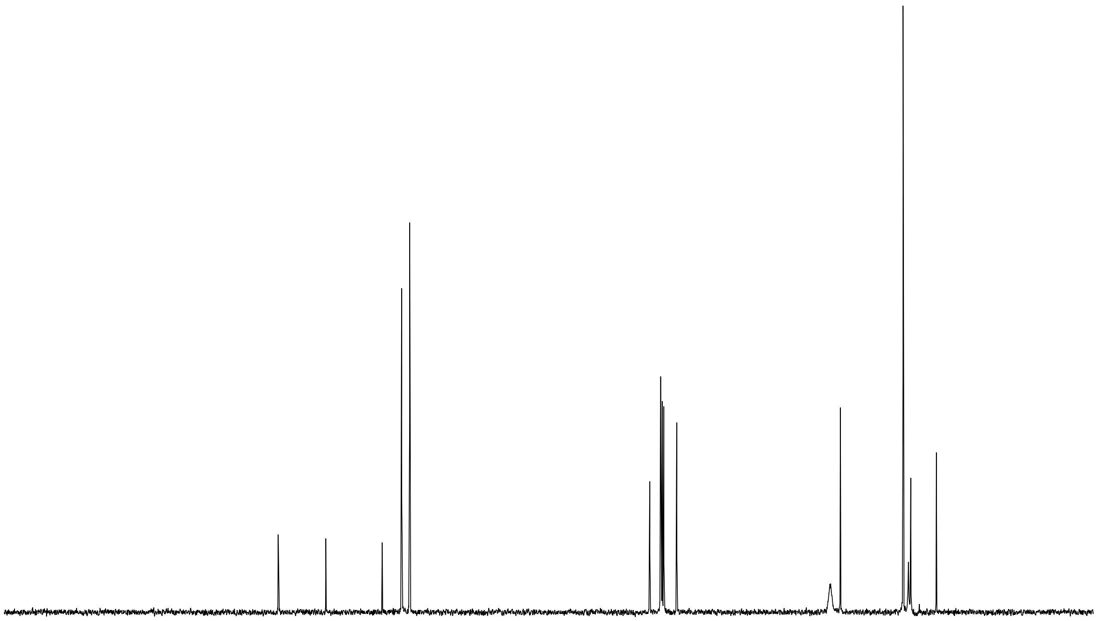

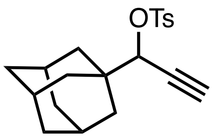
OTs

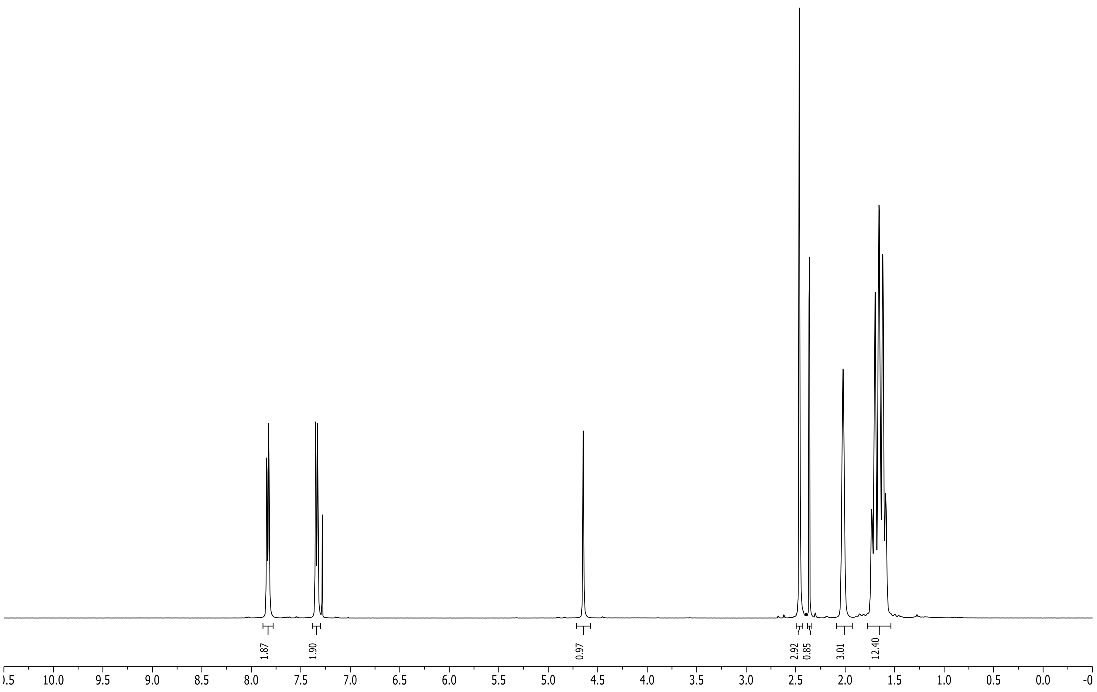

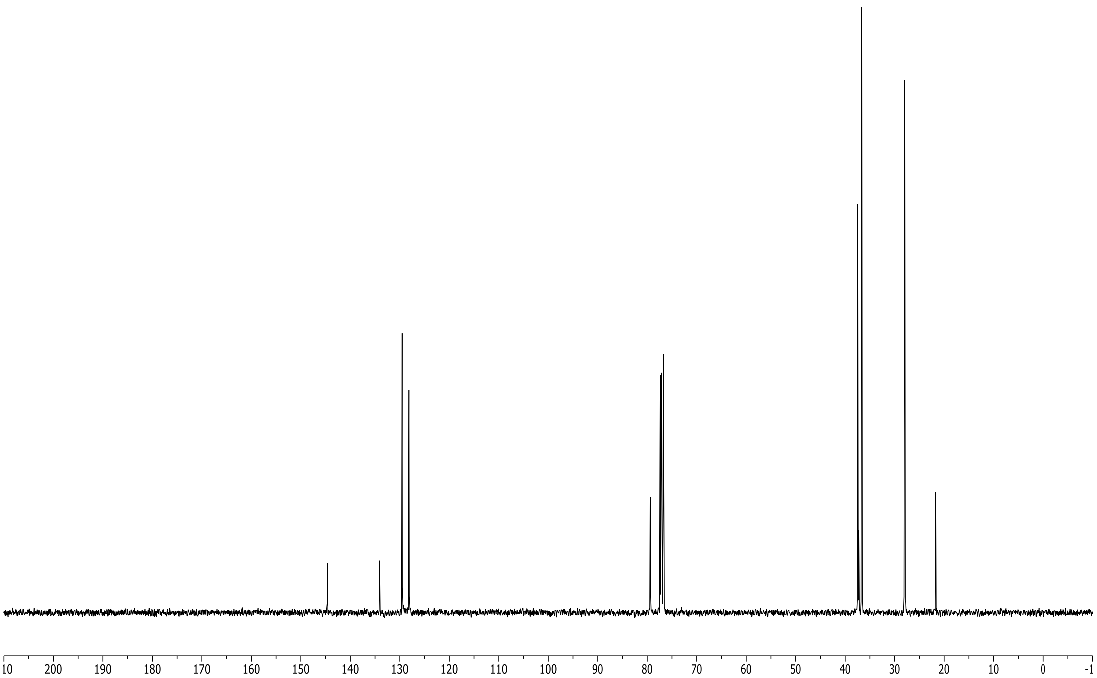

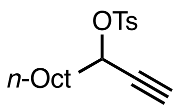
OTs
n-Oct

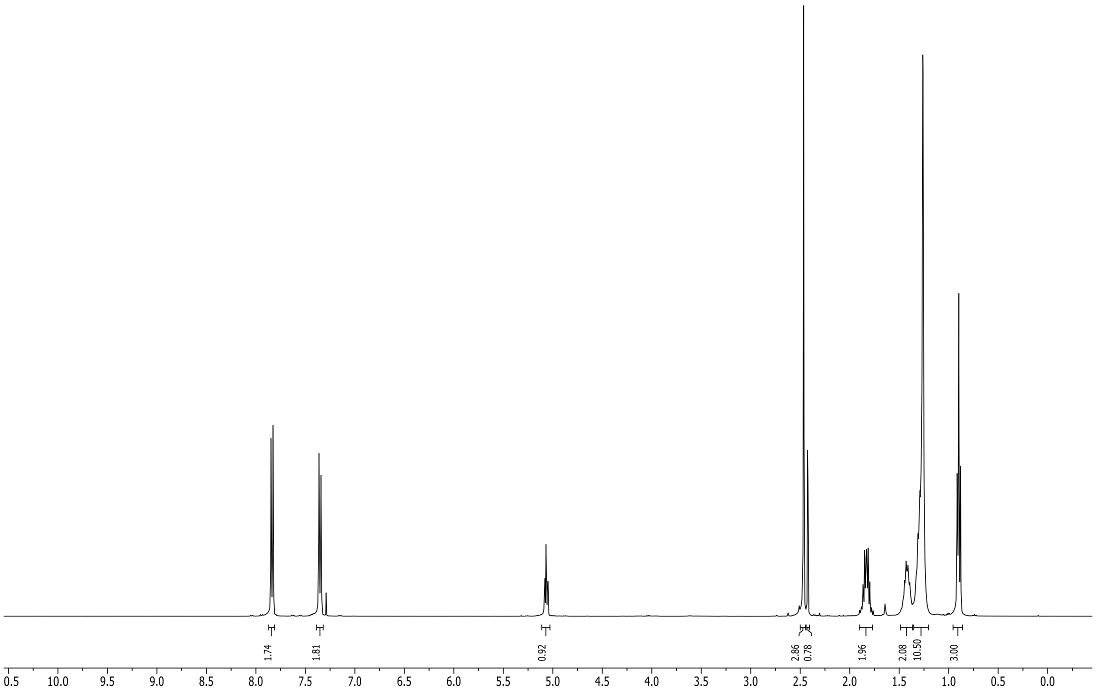

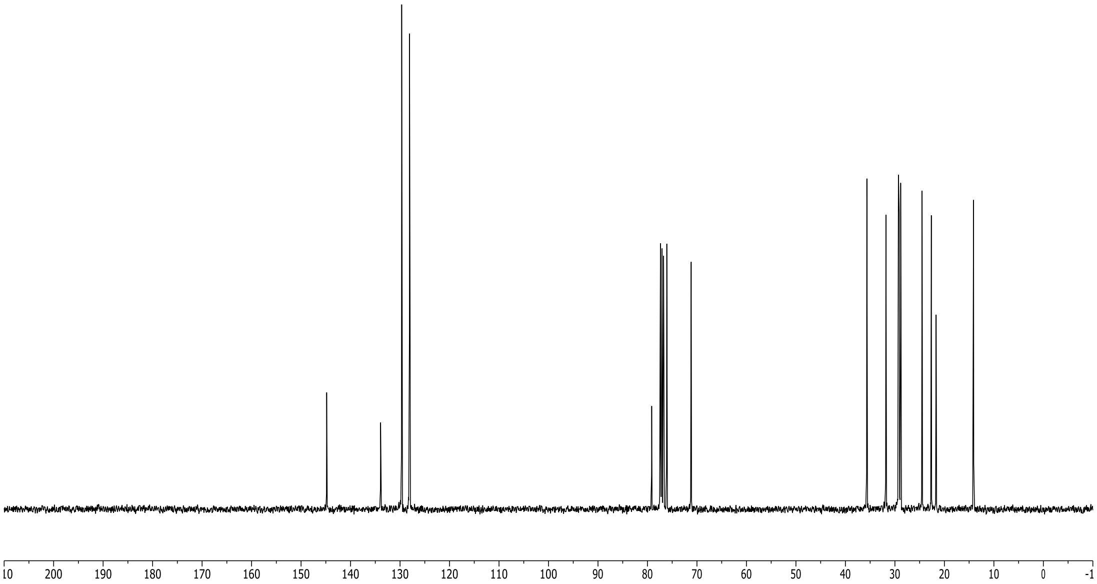

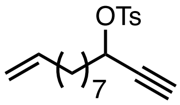
OTs
7

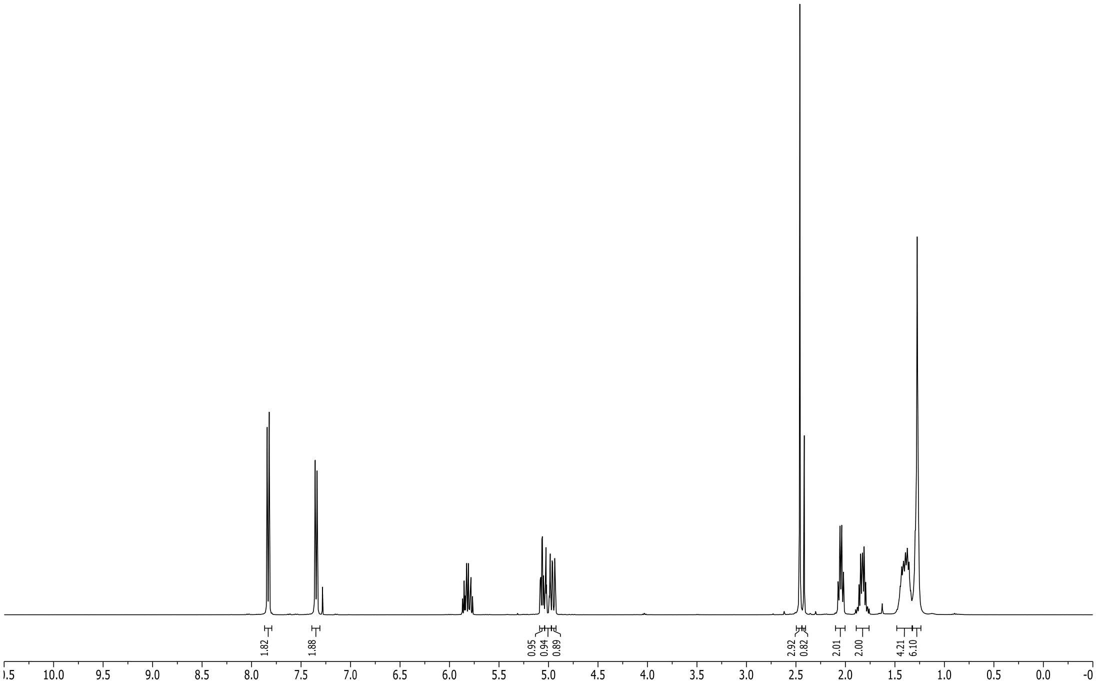
1.82
1.88
0.95
0.94
0.89
2.92
0.82
2.01
2.00
4.21
6.10
10.0 9.5 9.0 8.5 8.0 7.5 7.0 6.5 6.0 5.5 5.0 4.5 4.0 3.5 3.0 2.5 2.0 1.5 1.0 0.5 0.0

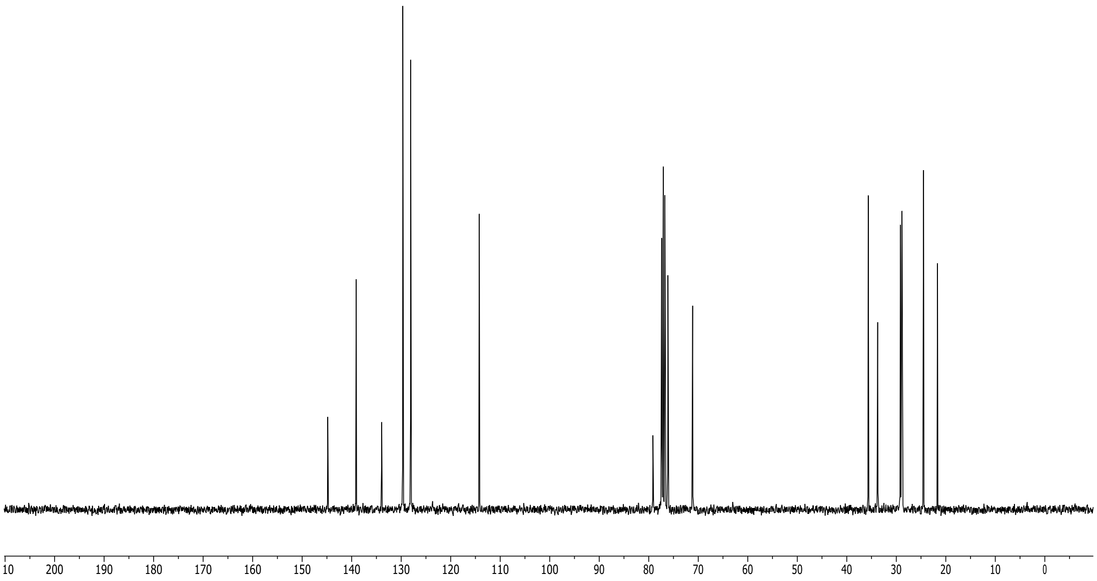
200 190 180 170 160 150 140 130 120 110 100 90 80 70 60 50 40 30 20 10 0

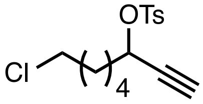
OTs
Cl
4

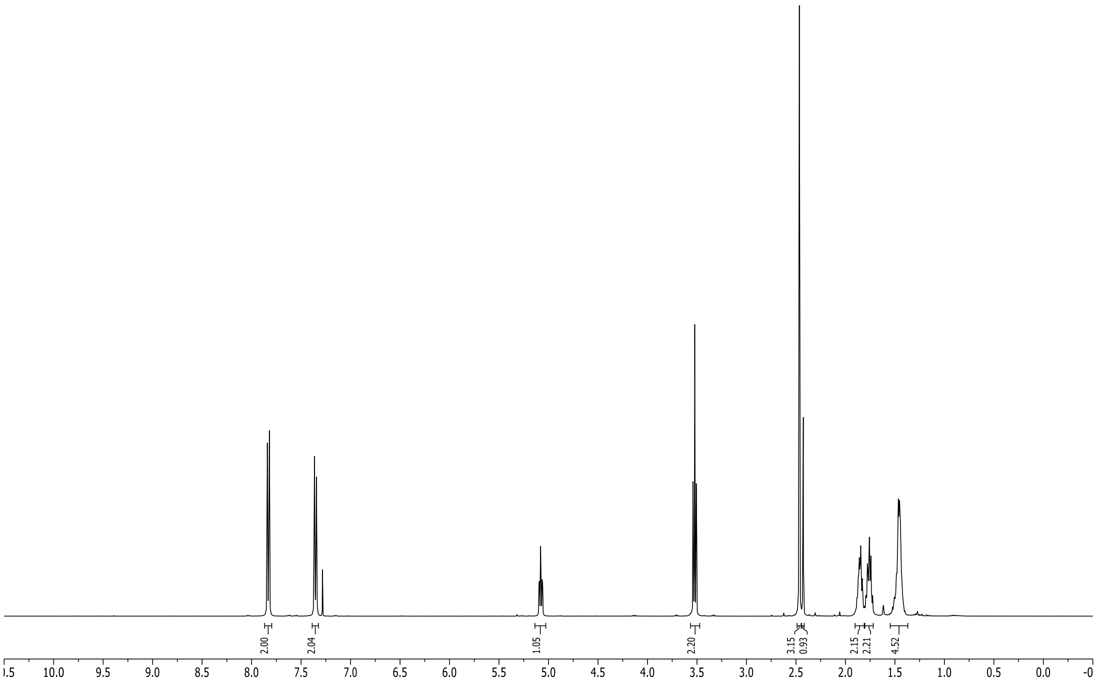

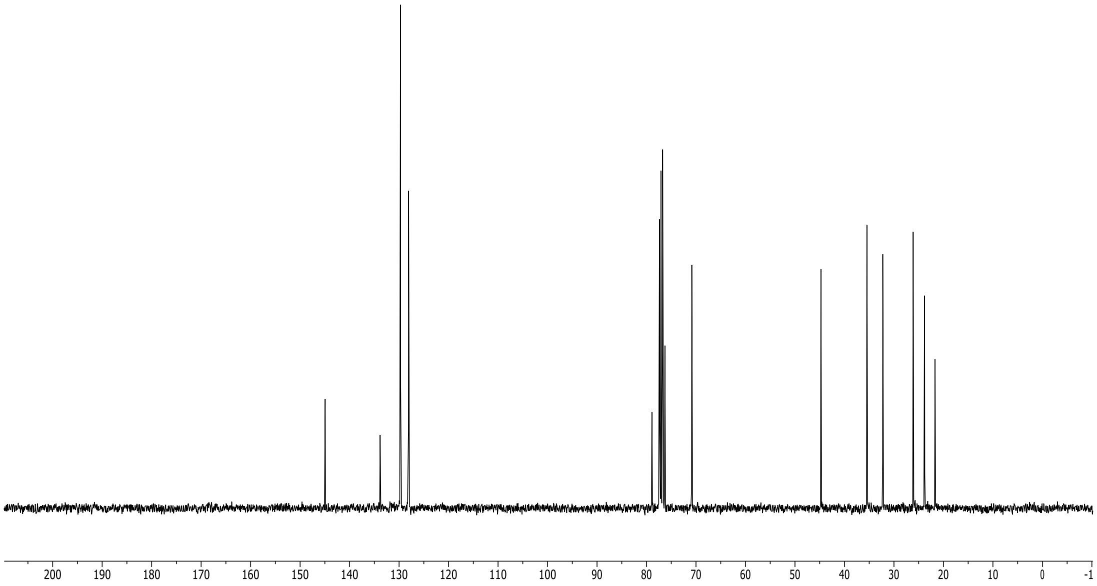

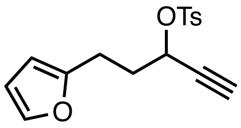
OTs
O

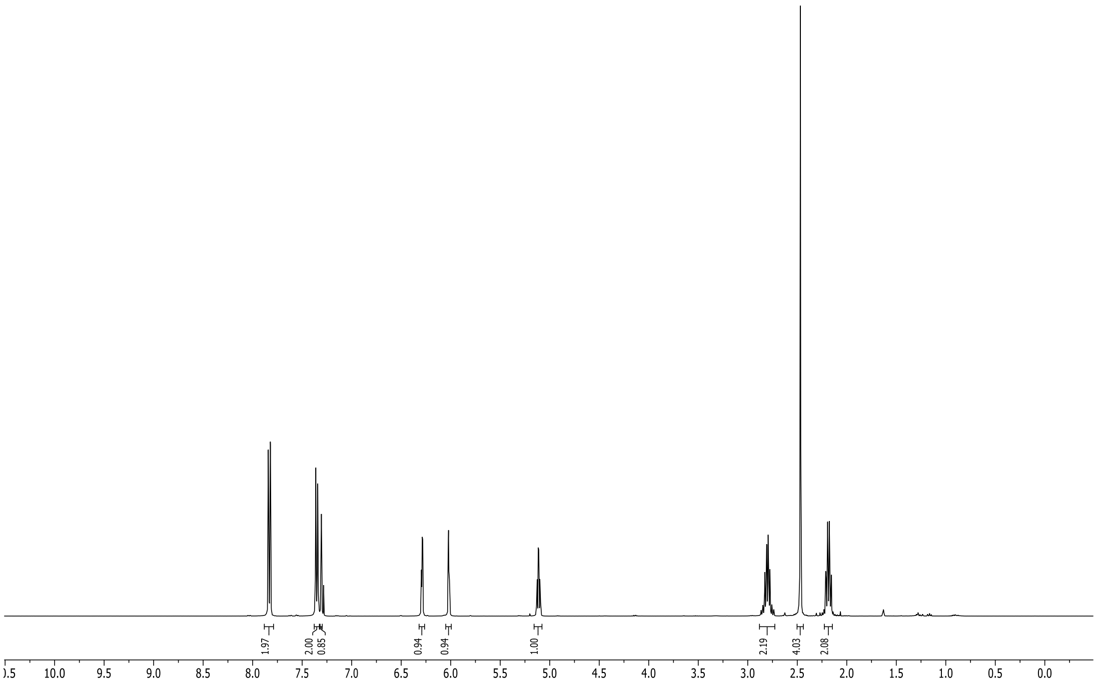
1.97
2.00
0.85
0.94
0.94
1.00
2.19
4.03
2.08
10.0
9.5
9.0
8.5
8.0
7.5
7.0
6.5
6.0
5.5
5.0
4.5
4.0
3.5
3.0
2.5
2.0
1.5
1.0
0.5
0.0

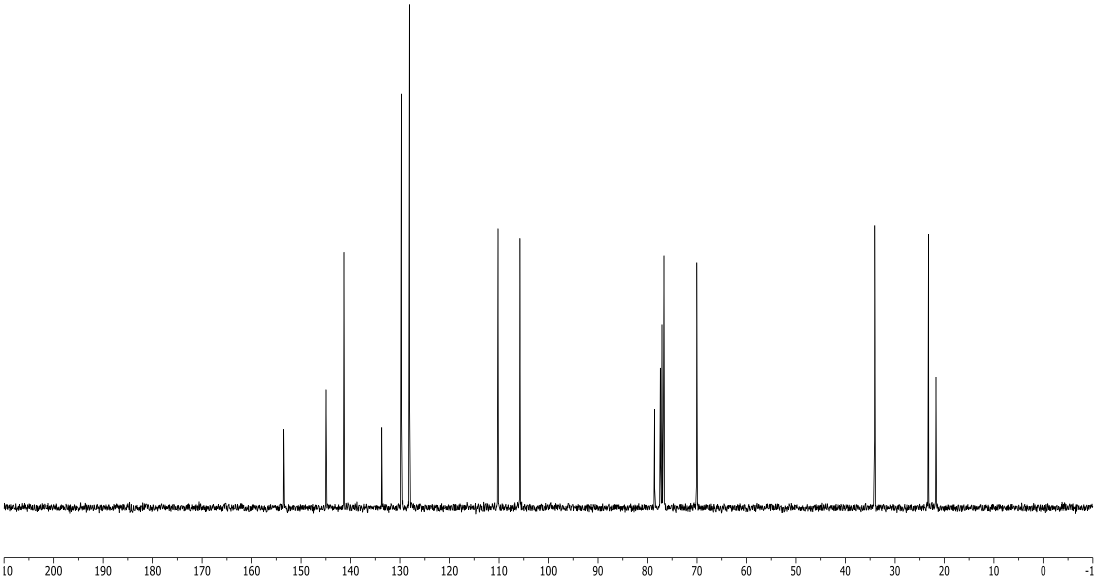
200
190
180
170
160
150
140
130
120
110
100
90
80
70
60
50
40
30
20
10
0

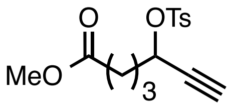
OTs
MeO
3

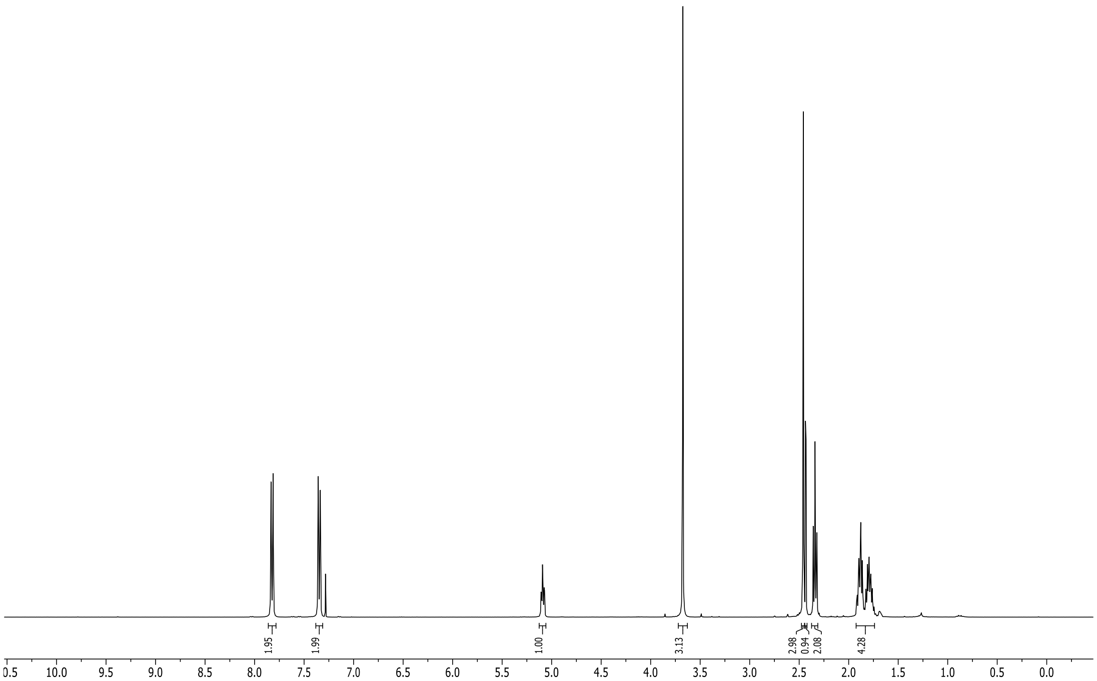

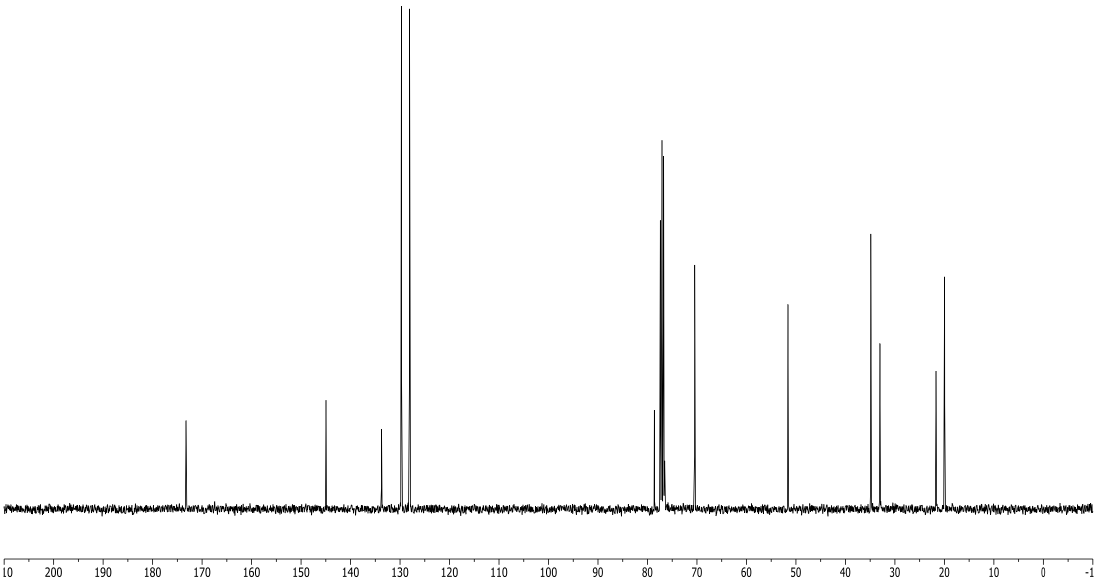

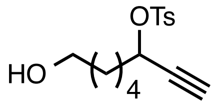
OTs
HO
4

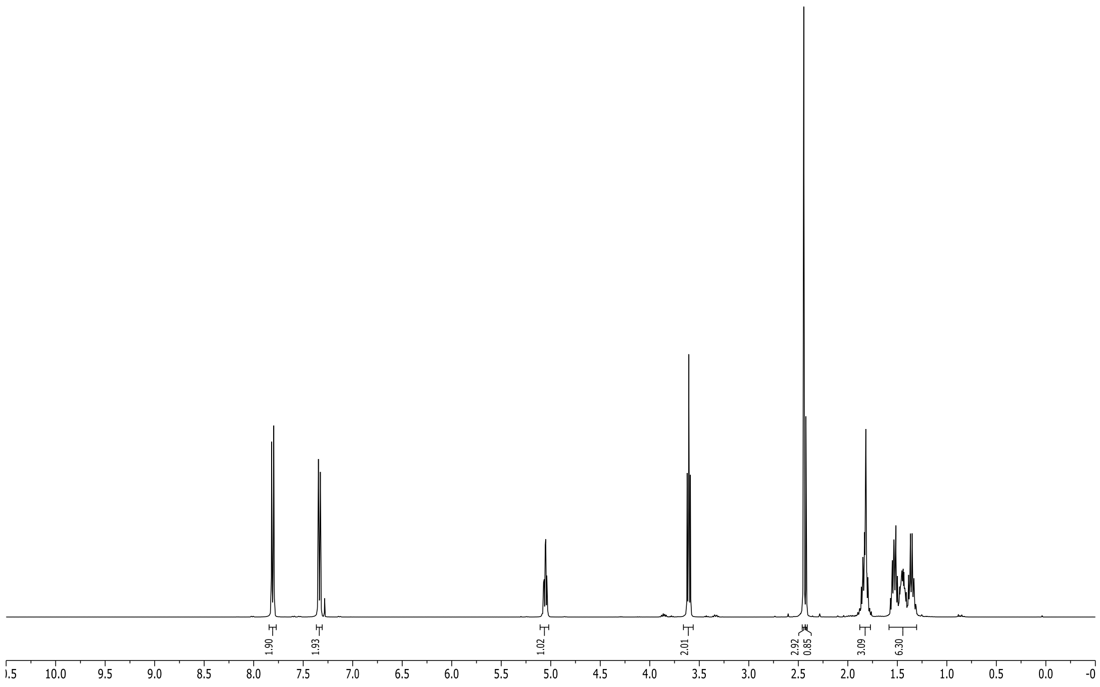
1.90
1.93
1.02
2.01
2.92
0.85
3.09
6.30
10.0 9.5 9.0 8.5 8.0 7.5 7.0 6.5 6.0 5.5 5.0 4.5 4.0 3.5 3.0 2.5 2.0 1.5 1.0 0.5 0.0

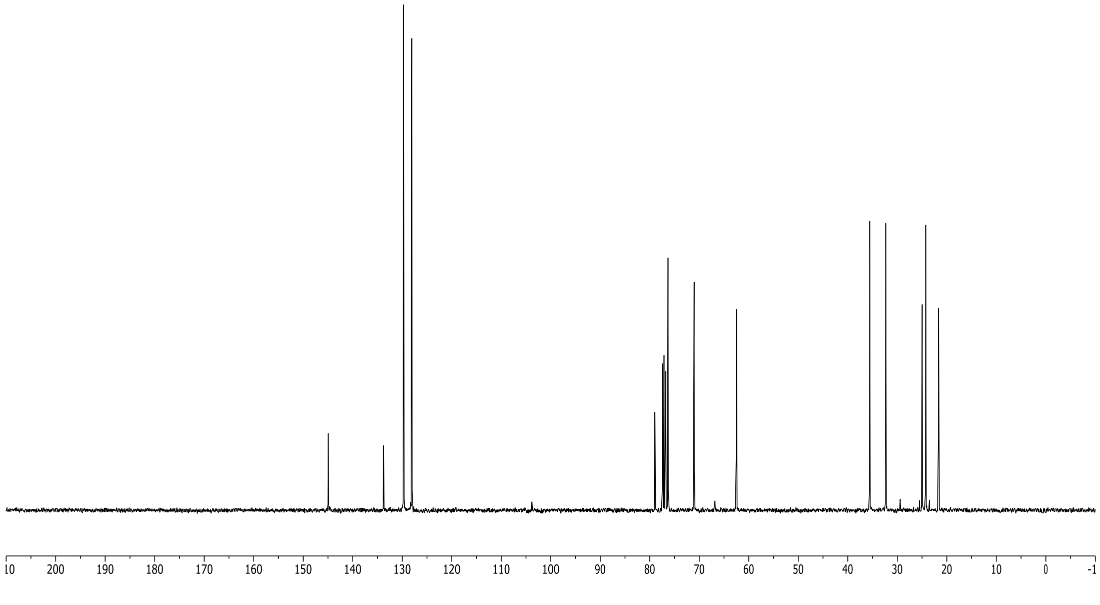
200 190 180 170 160 150 140 130 120 110 100 90 80 70 60 50 40 30 20 10 0

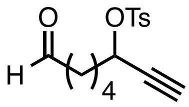
O
OTs
H
4

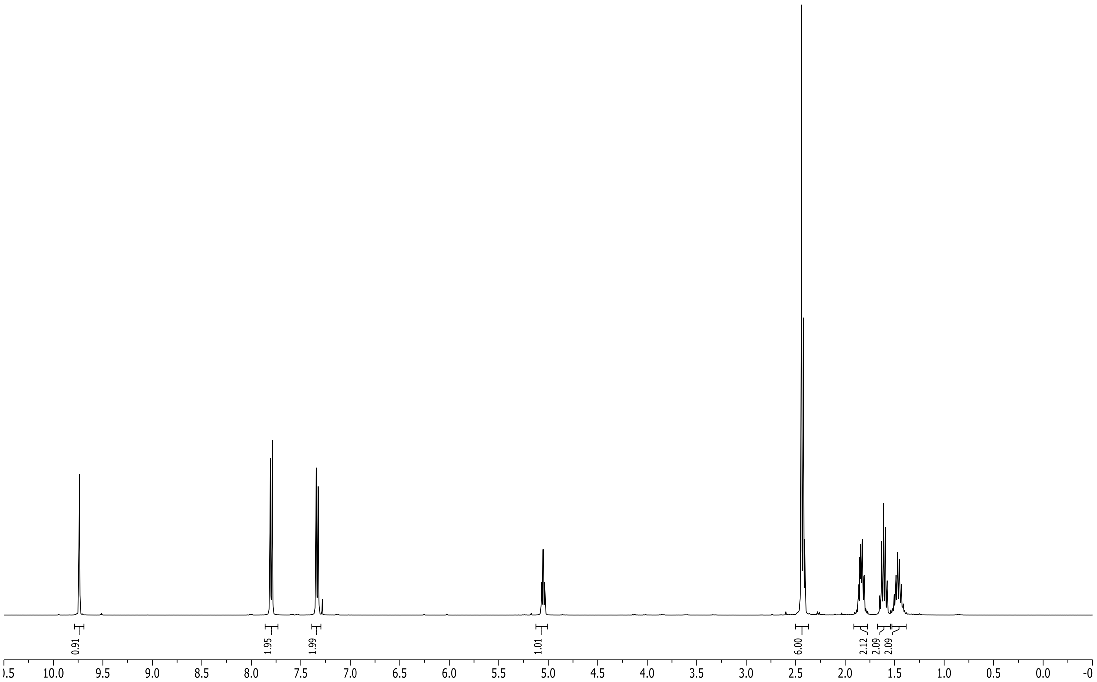
0.91
1.95
1.99
1.01
6.00
2.12
2.09
2.09
10.0
9.5
9.0
8.5
8.0
7.5
7.0
6.5
6.0
5.5
5.0
4.5
4.0
3.5
3.0
2.5
2.0
1.5
1.0
0.5
0.0

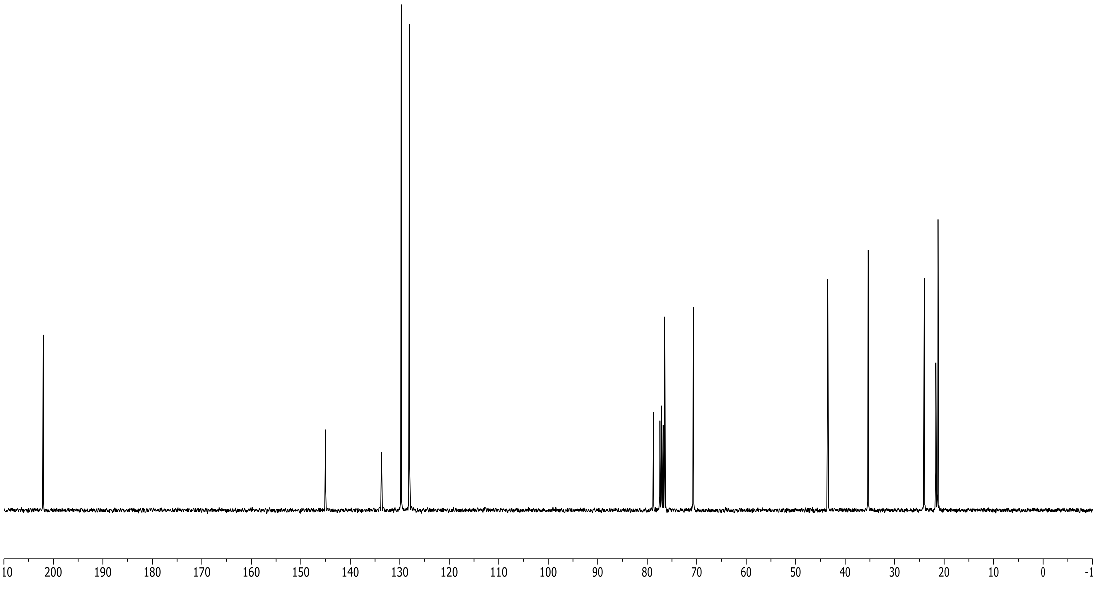
200
190
180
170
160
150
140
130
120
110
100
90
80
70
60
50
40
30
20
10
0

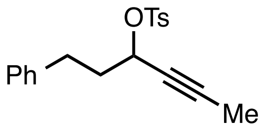
OTs
Ph
Me

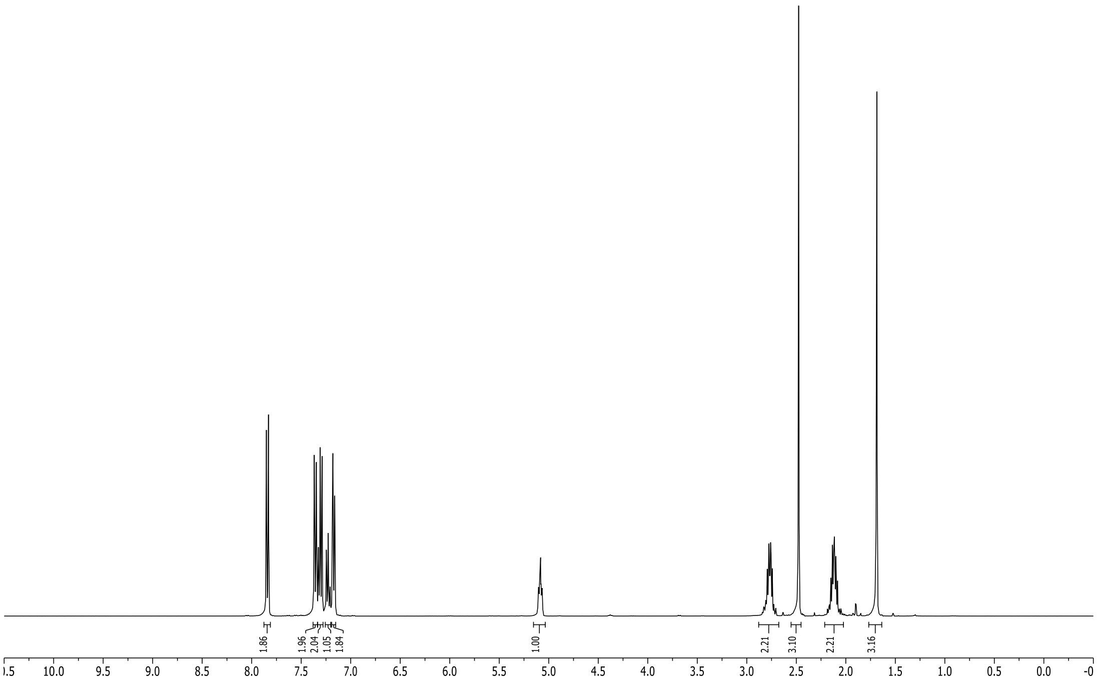

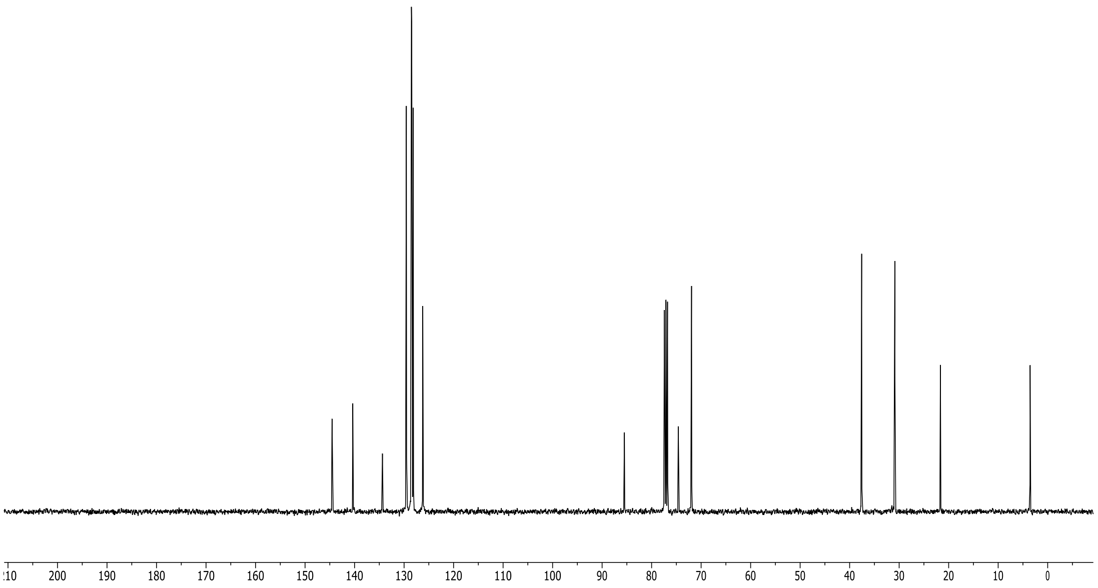

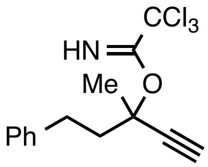
CCl$_3$
HN
Me
O
Ph

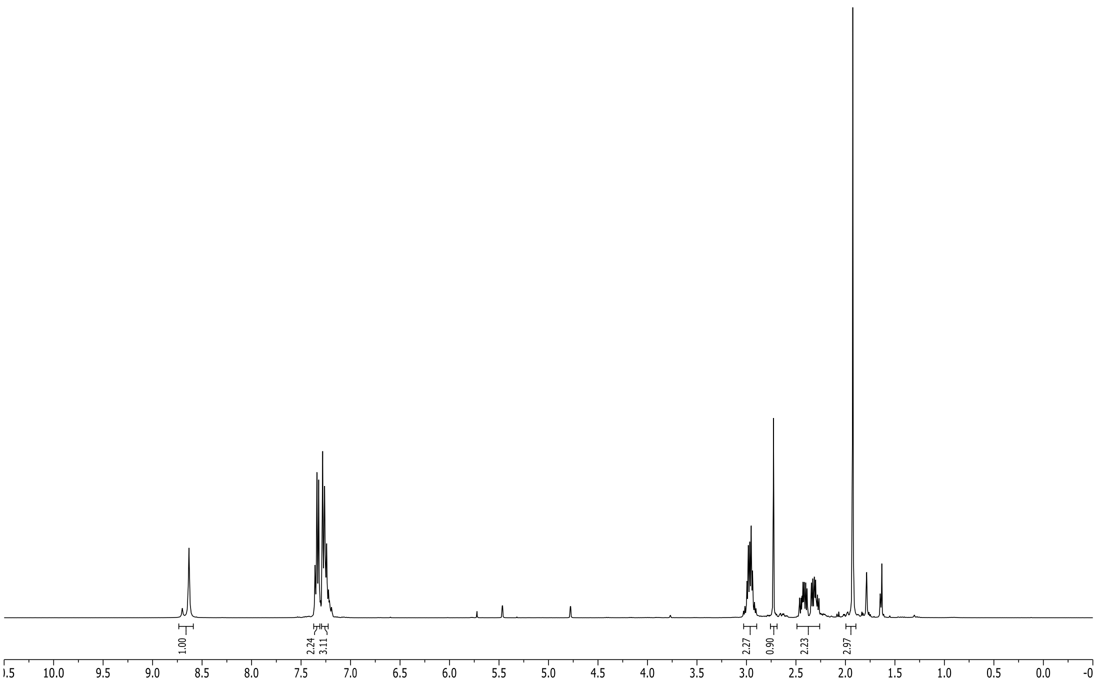

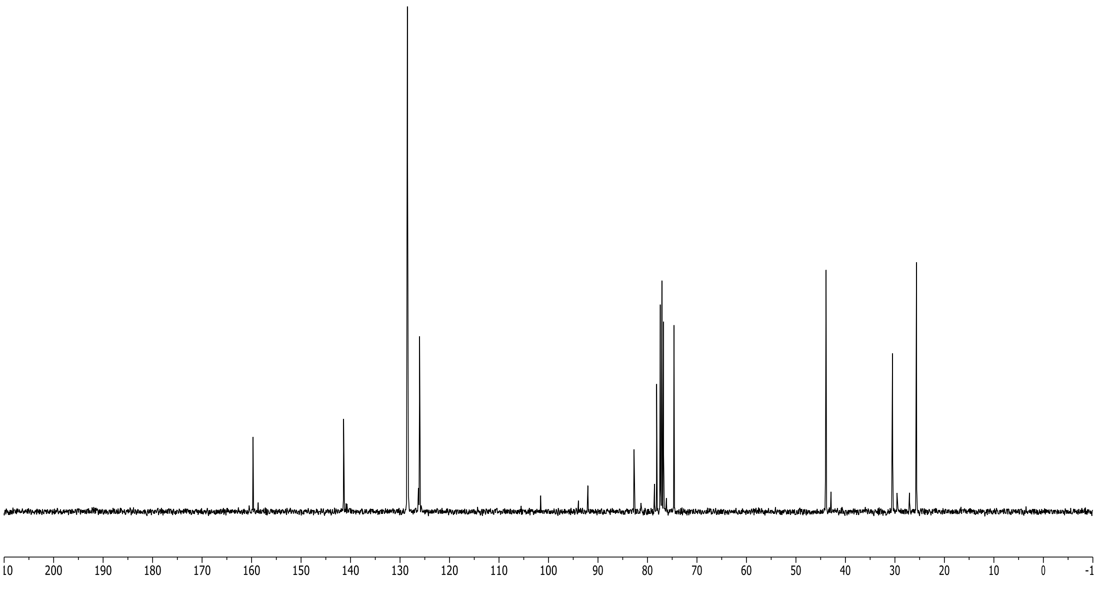

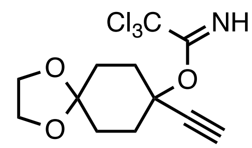
Cl3C
NH
O
O
O

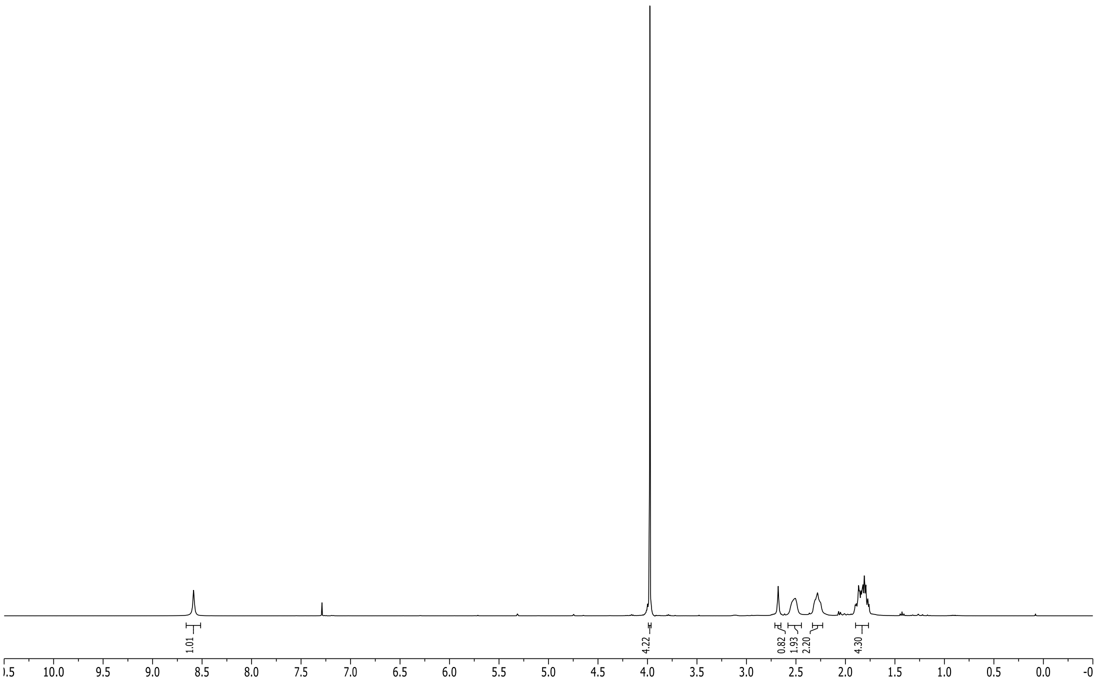
1.01
4.22
0.82
1.93
2.20
4.30
10.0 9.5 9.0 8.5 8.0 7.5 7.0 6.5 6.0 5.5 5.0 4.5 4.0 3.5 3.0 2.5 2.0 1.5 1.0 0.5 0.0

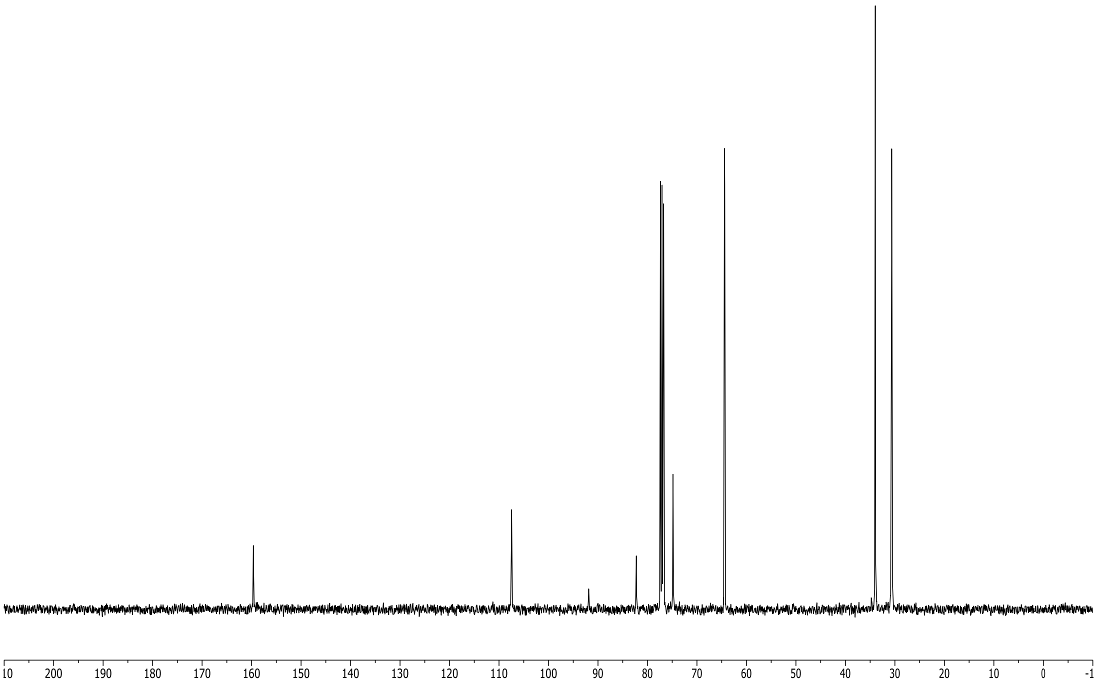
200 190 180 170 160 150 140 130 120 110 100 90 80 70 60 50 40 30 20 10 0

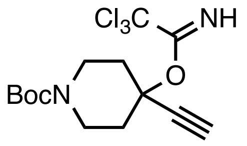
Cl3C
NH
O
BocN

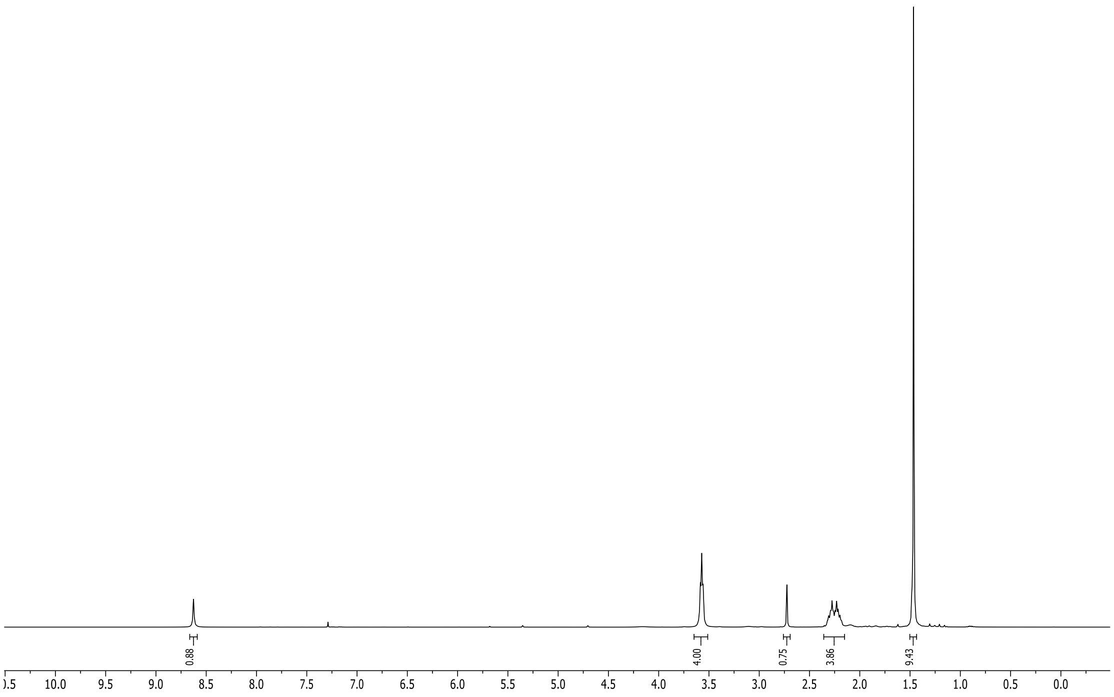
0.88
4.00
0.75
3.86
9.43
10.0 9.5 9.0 8.5 8.0 7.5 7.0 6.5 6.0 5.5 5.0 4.5 4.0 3.5 3.0 2.5 2.0 1.5 1.0 0.5 0.0

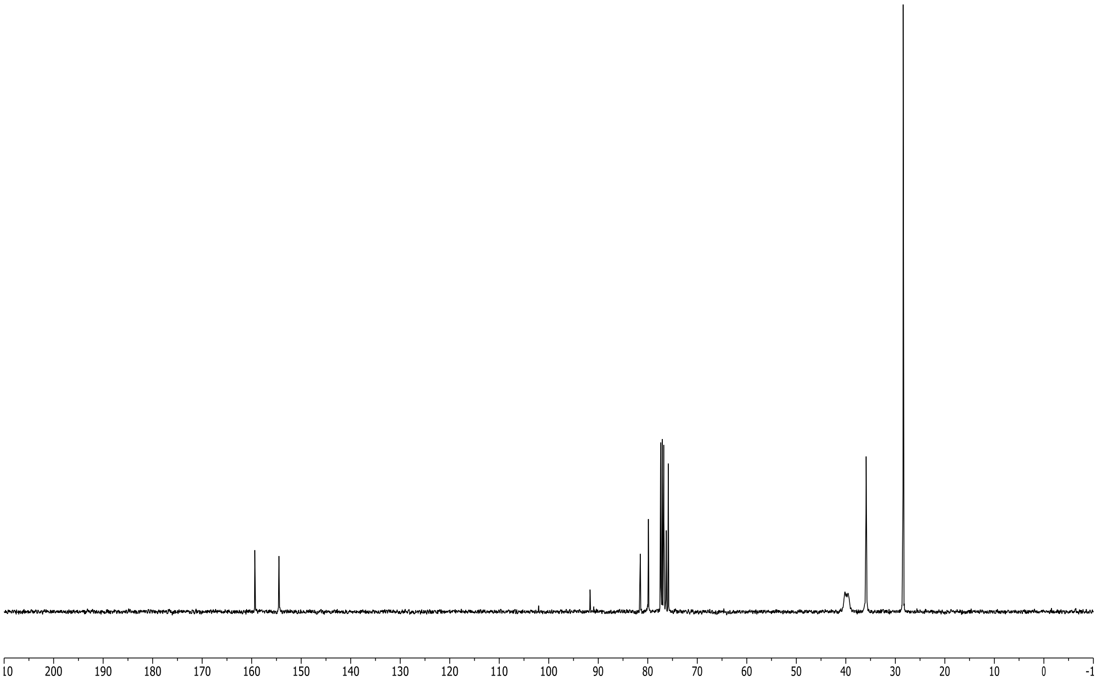
200 190 180 170 160 150 140 130 120 110 100 90 80 70 60 50 40 30 20 10 0

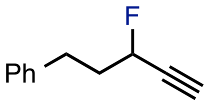
F
Ph

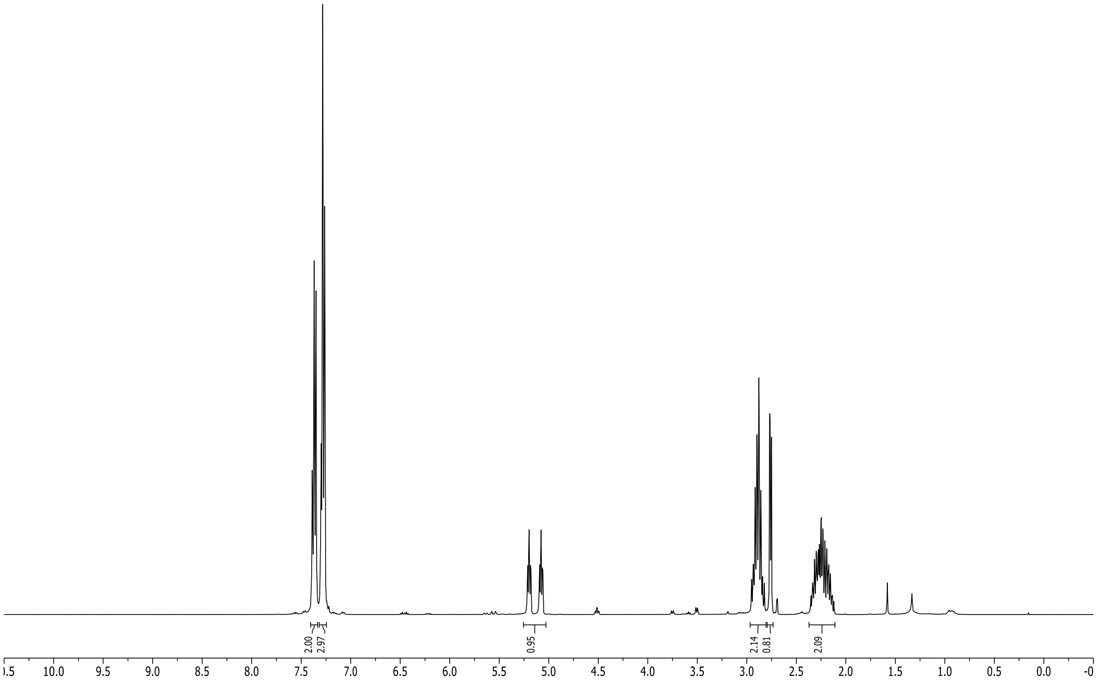
2.00
2.97
0.95
2.14
0.81
2.09

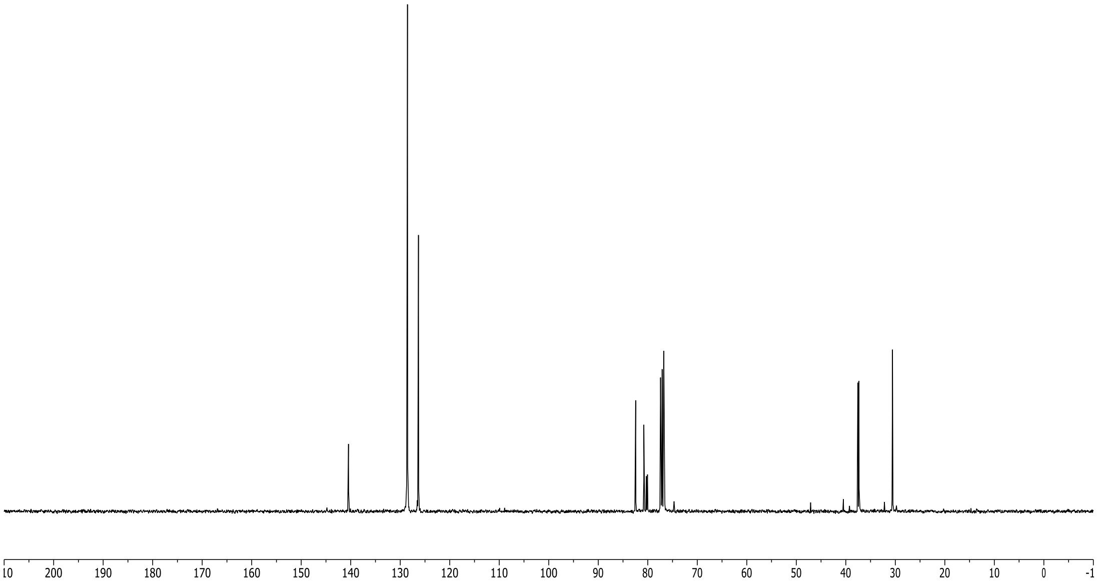

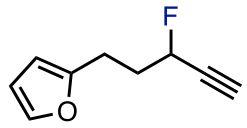

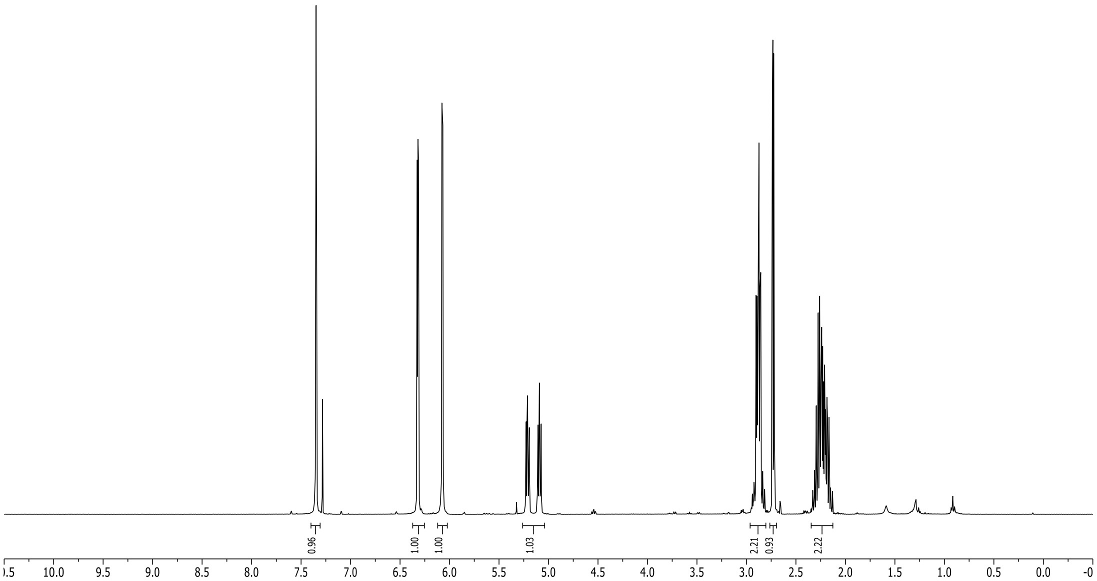
0.96
1.00
1.00
1.03
2.21
0.93
2.22
10.0 9.5 9.0 8.5 8.0 7.5 7.0 6.5 6.0 5.5 5.0 4.5 4.0 3.5 3.0 2.5 2.0 1.5 1.0 0.5 0.0

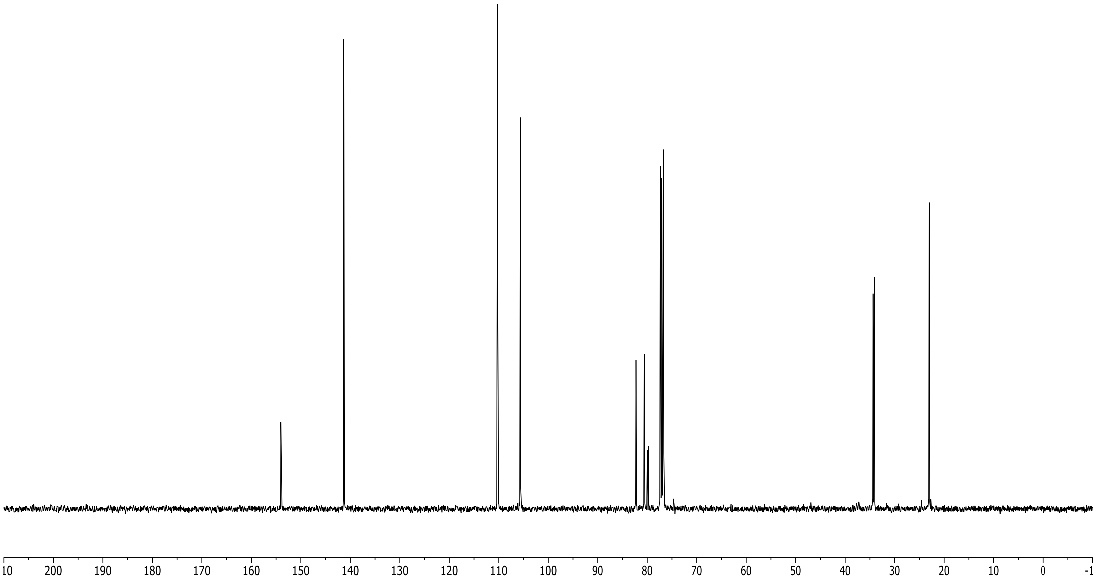
200 190 180 170 160 150 140 130 120 110 100 90 80 70 60 50 40 30 20 10 0

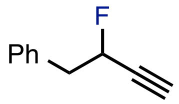
F
Ph

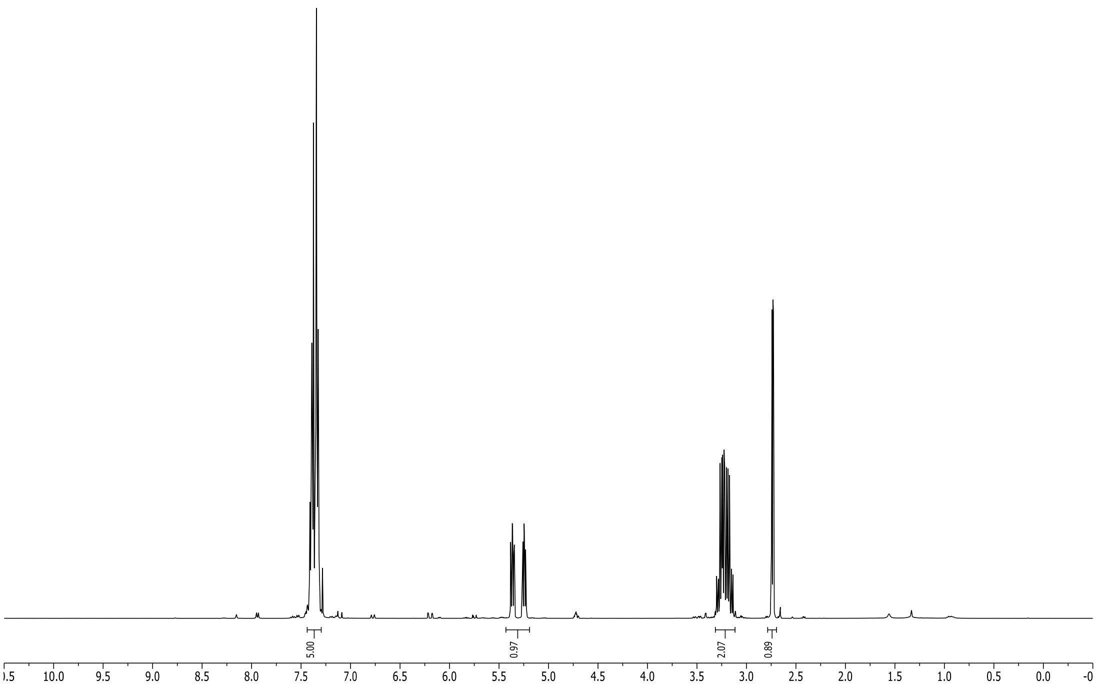

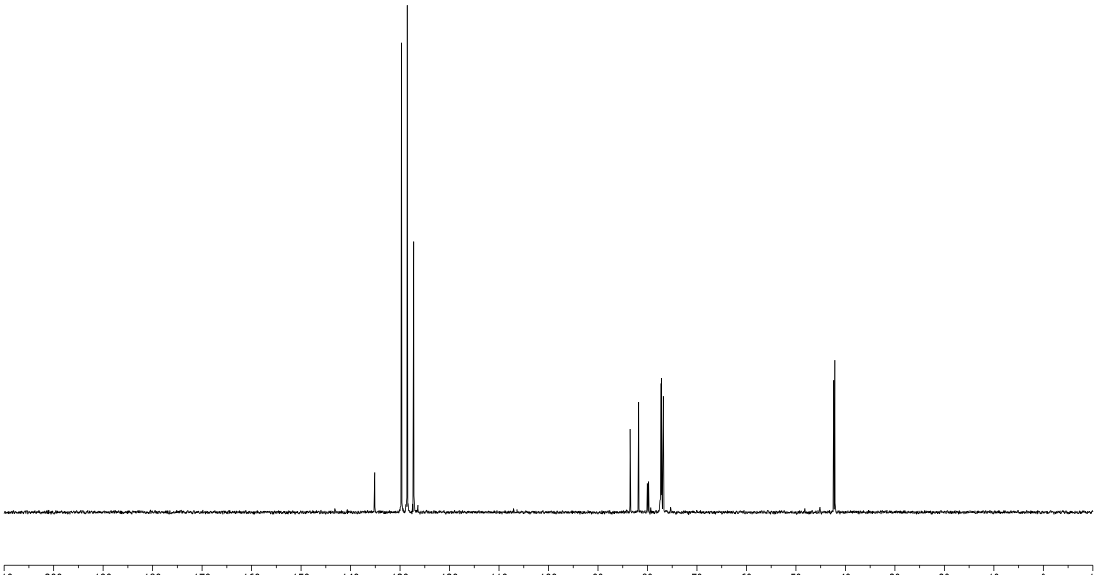

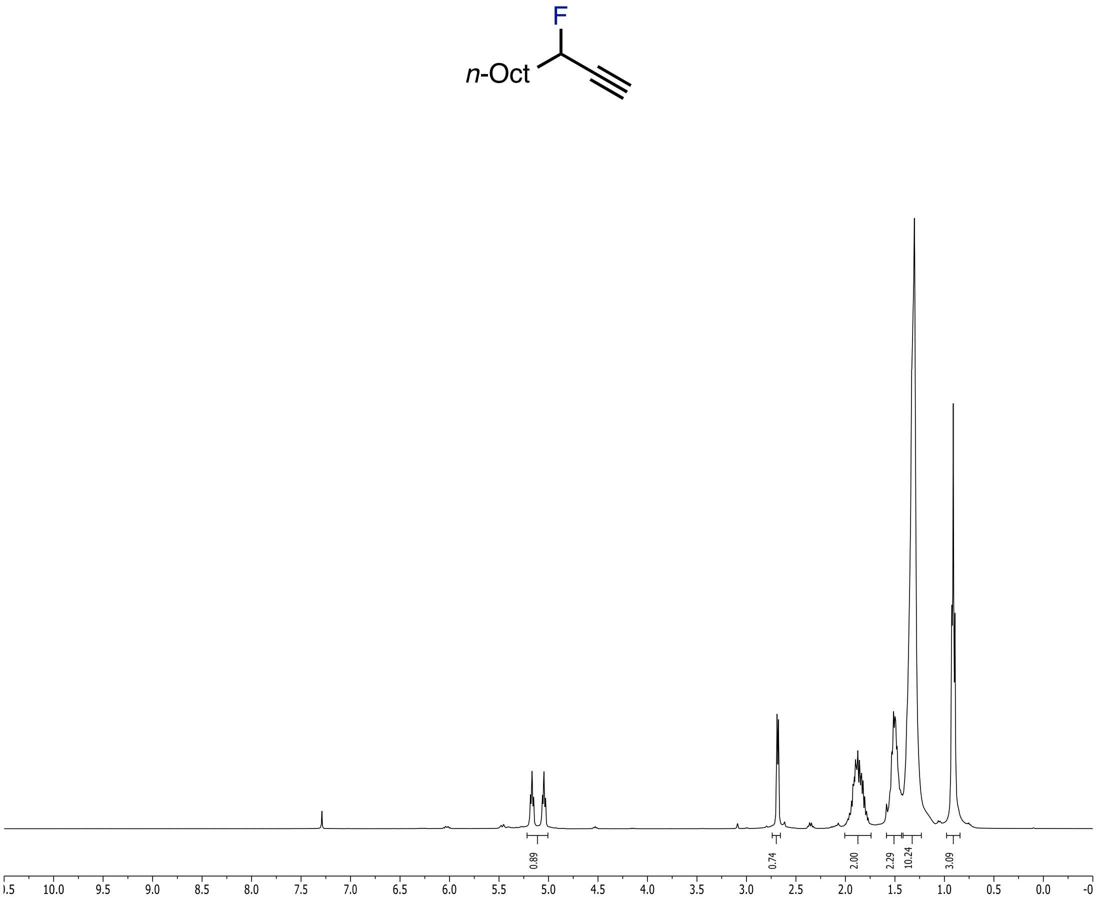
F
n-Oct
0.89
0.74
2.00
2.29
10.24
3.09
10.0 9.5 9.0 8.5 8.0 7.5 7.0 6.5 6.0 5.5 5.0 4.5 4.0 3.5 3.0 2.5 2.0 1.5 1.0 0.5 0.0

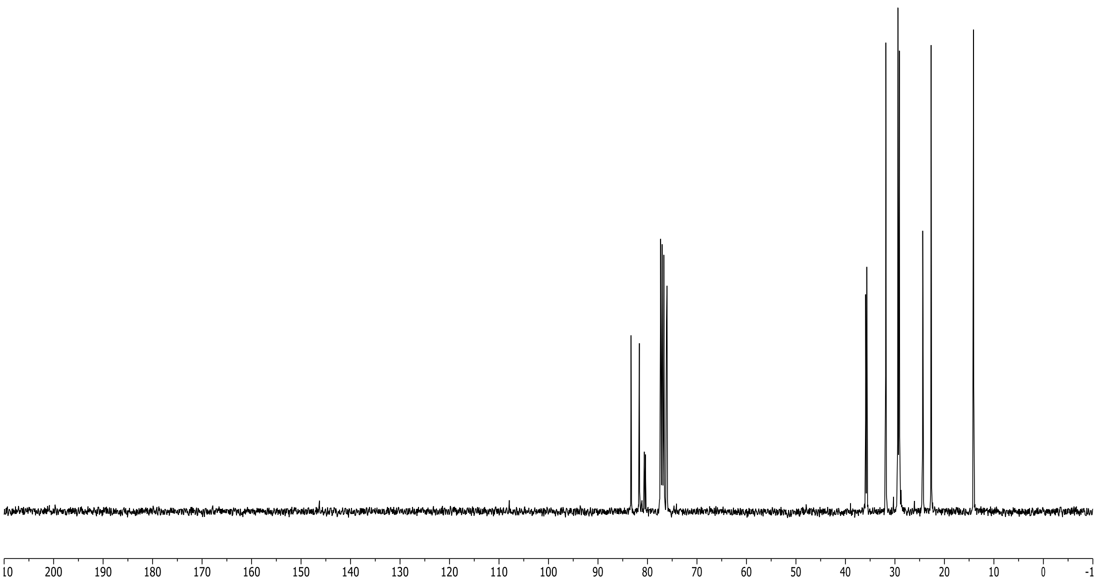
200 190 180 170 160 150 140 130 120 110 100 90 80 70 60 50 40 30 20 10 0

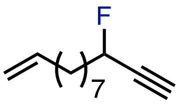
F
7

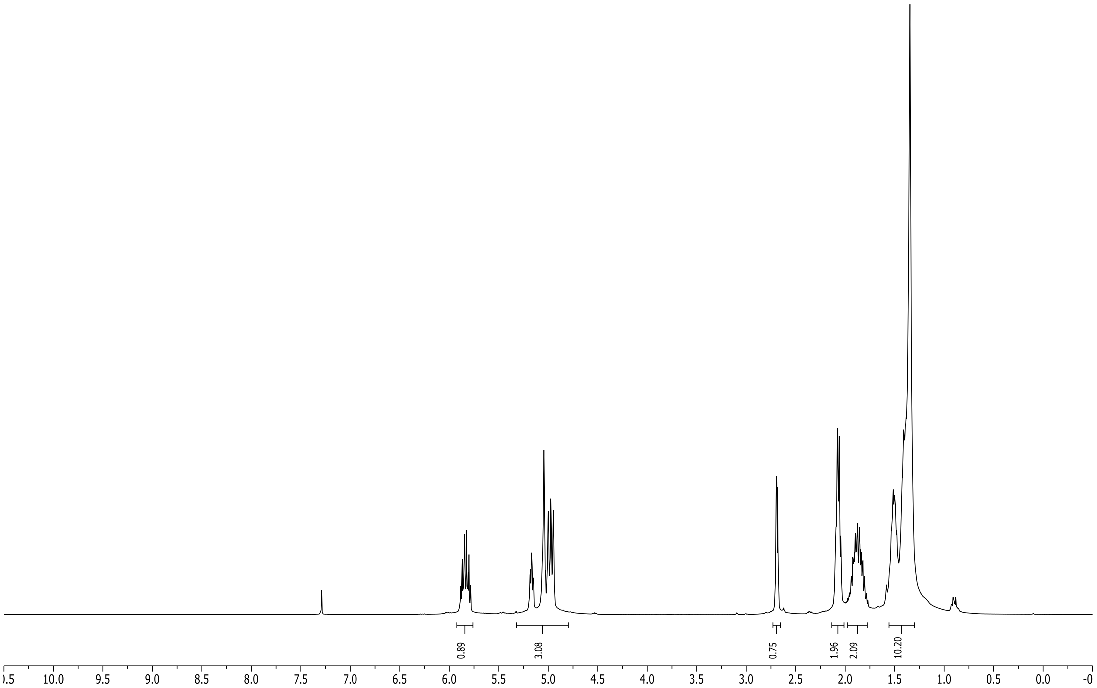
0.89
3.08
0.75
1.96
2.09
10.20
10.0 9.5 9.0 8.5 8.0 7.5 7.0 6.5 6.0 5.5 5.0 4.5 4.0 3.5 3.0 2.5 2.0 1.5 1.0 0.5 0.0

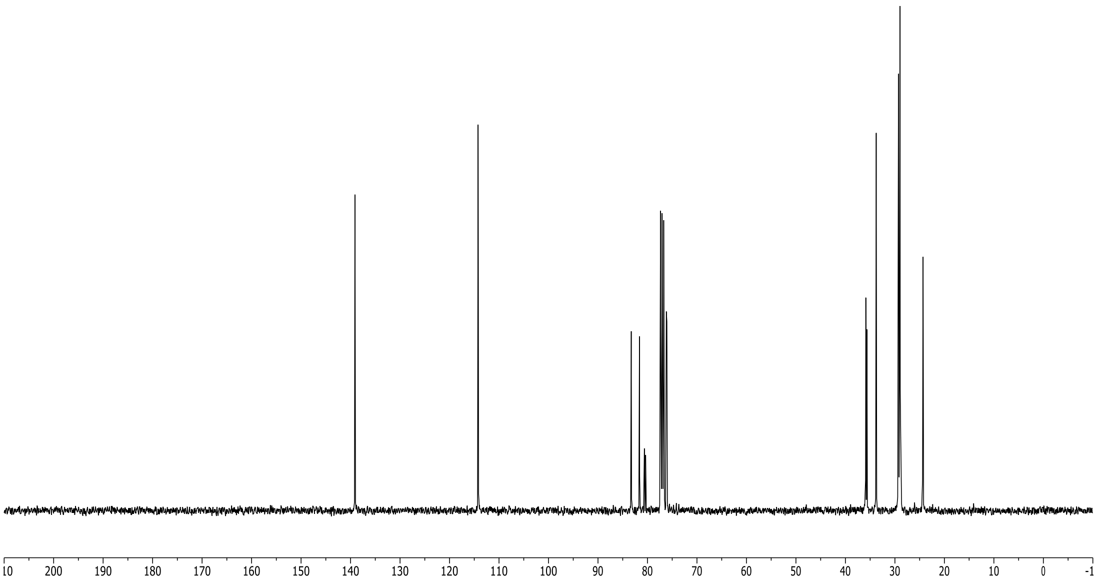
200 190 180 170 160 150 140 130 120 110 100 90 80 70 60 50 40 30 20 10 0

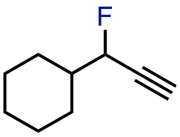
F

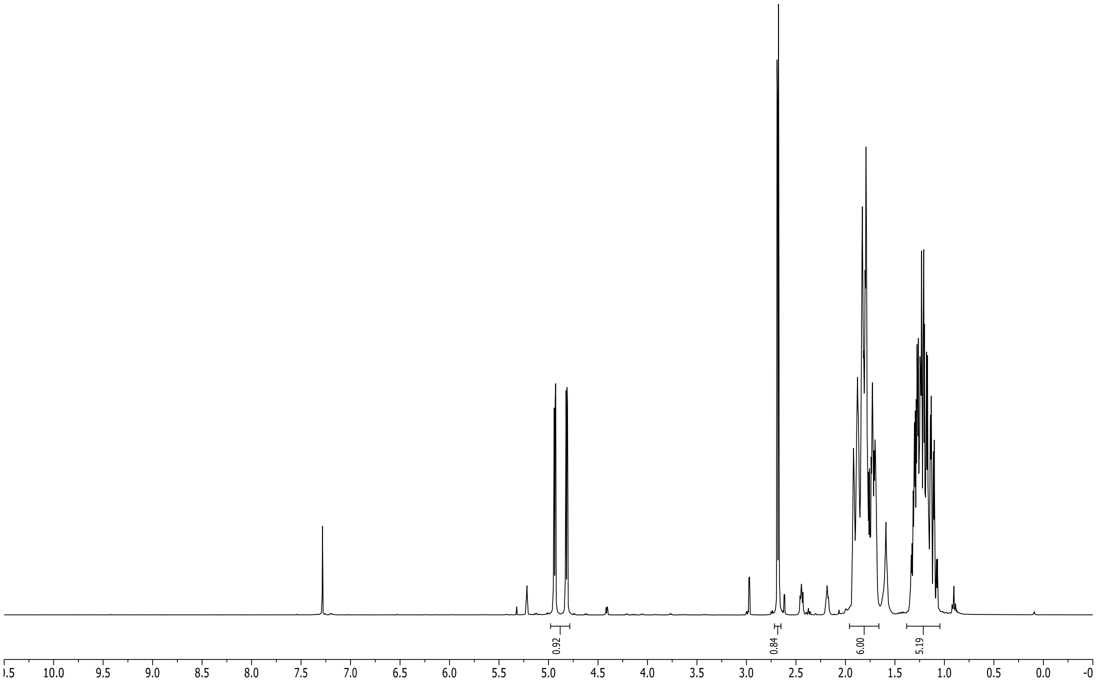

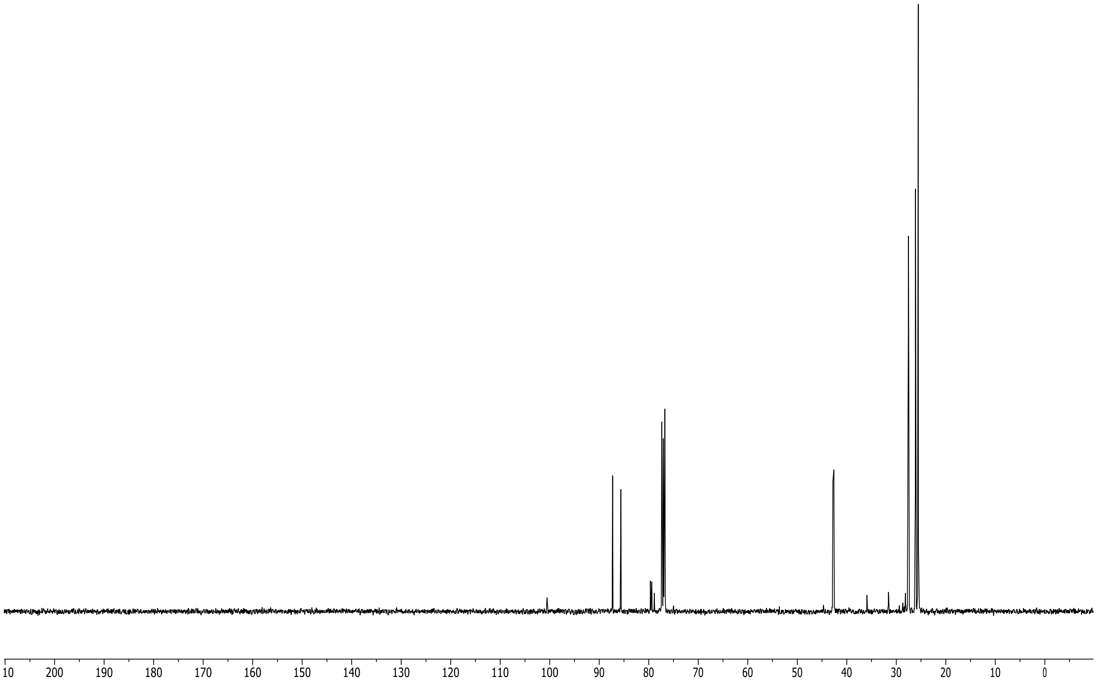

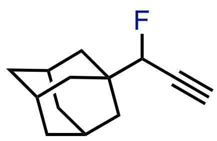

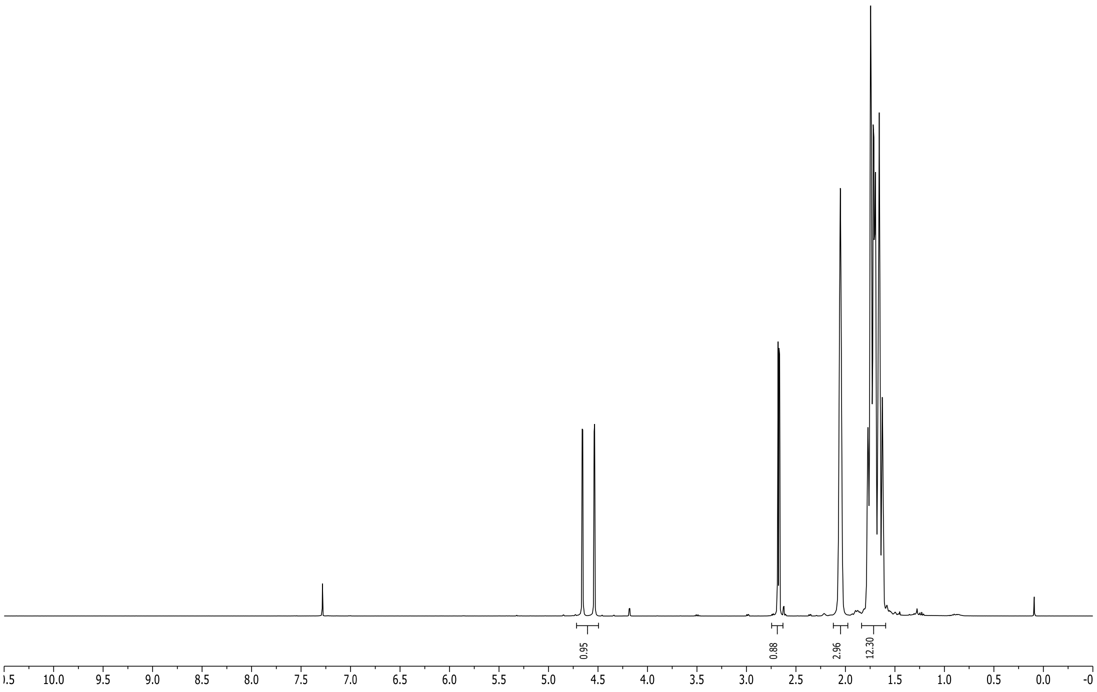
0.95
0.88
2.96
12.30
10.0
9.5
9.0
8.5
8.0
7.5
7.0
6.5
6.0
5.5
5.0
4.5
4.0
3.5
3.0
2.5
2.0
1.5
1.0
0.5
0.0

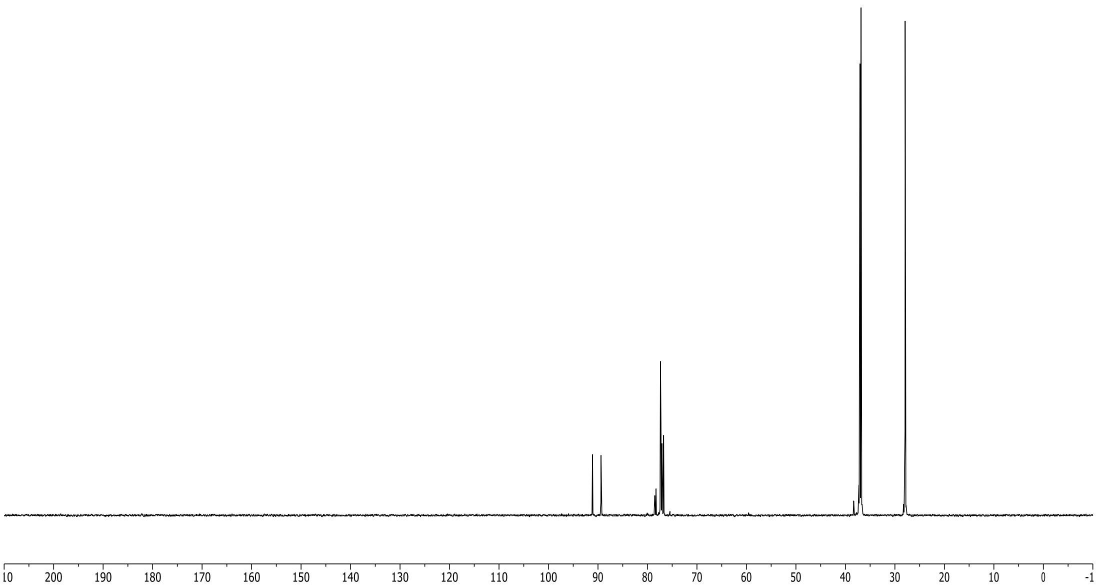
200
190
180
170
160
150
140
130
120
110
100
90
80
70
60
50
40
30
20
10
0

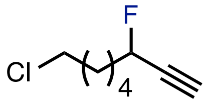
F
Cl
4

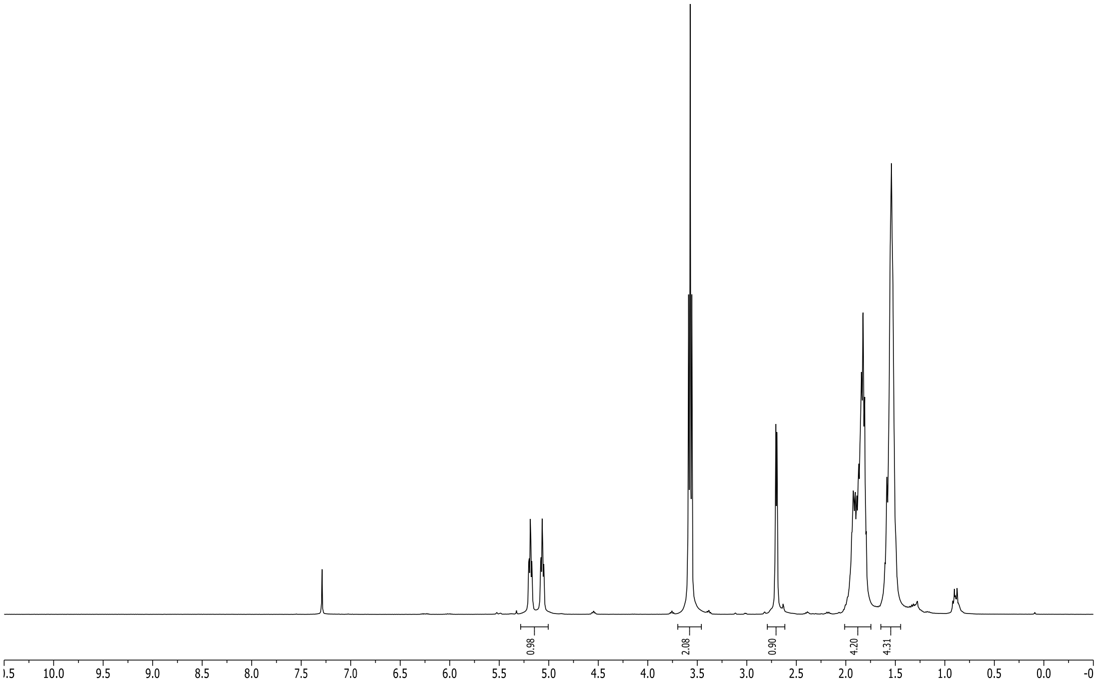
0.98
2.08
0.90
4.20
4.31

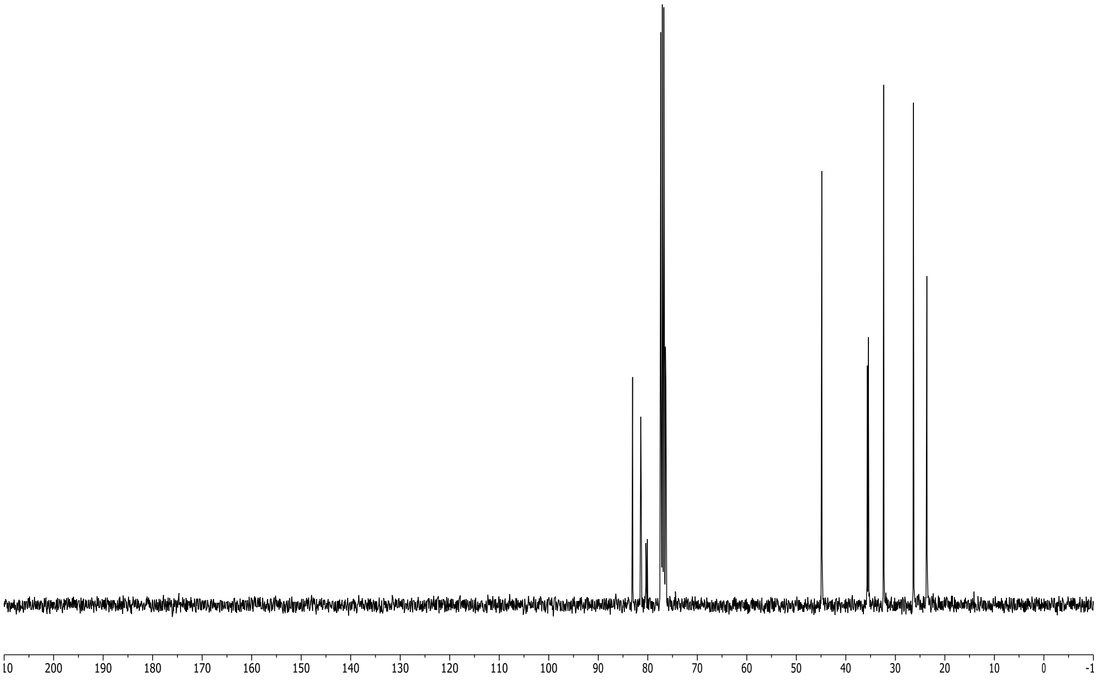

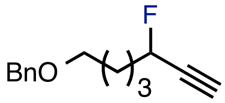
F
BnO
3

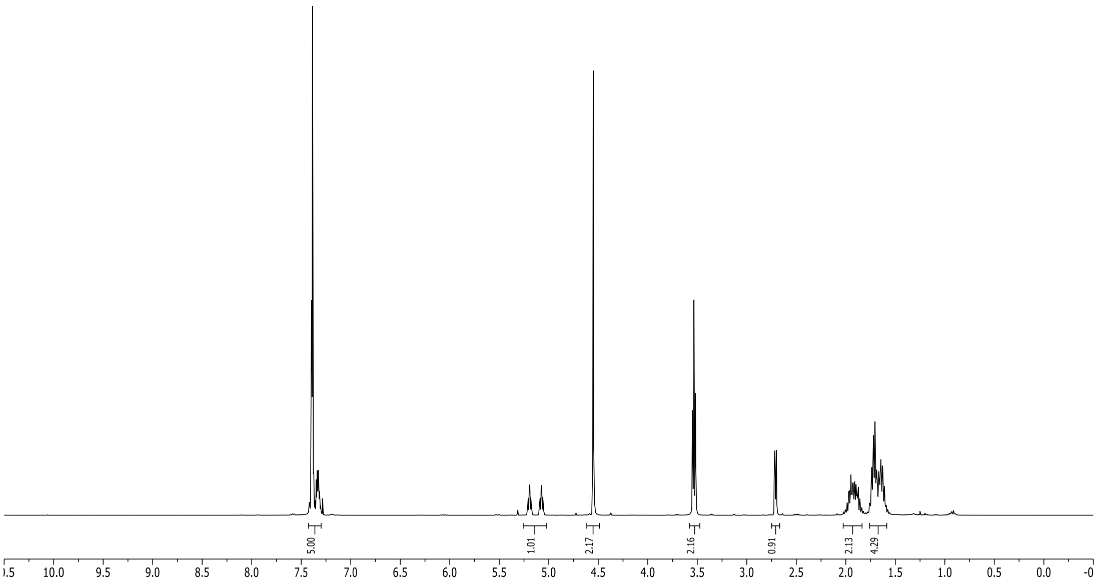

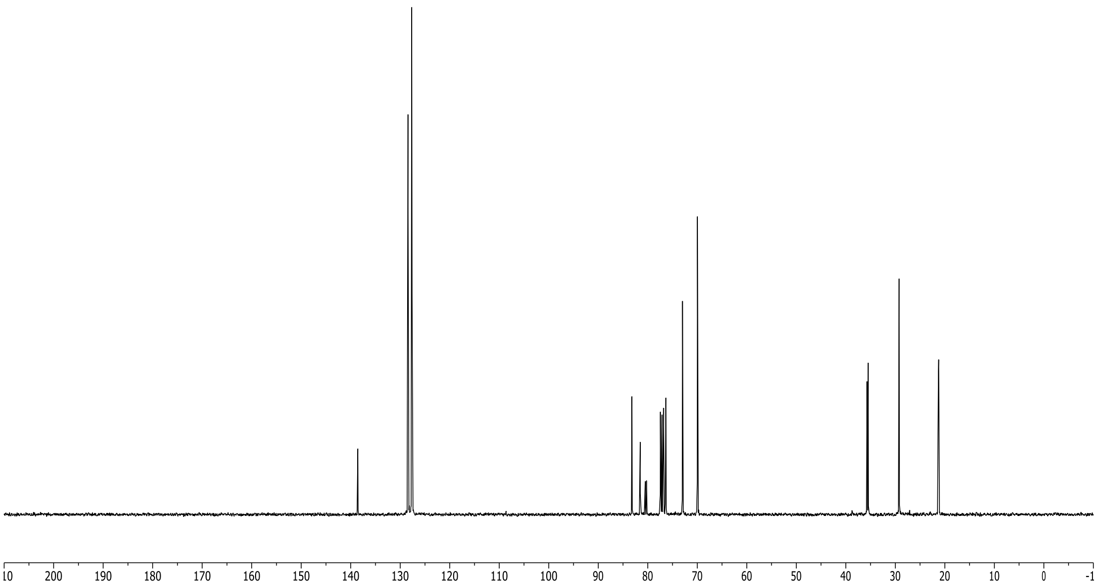

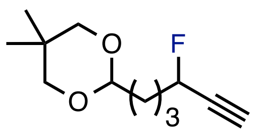
F
O
O
3

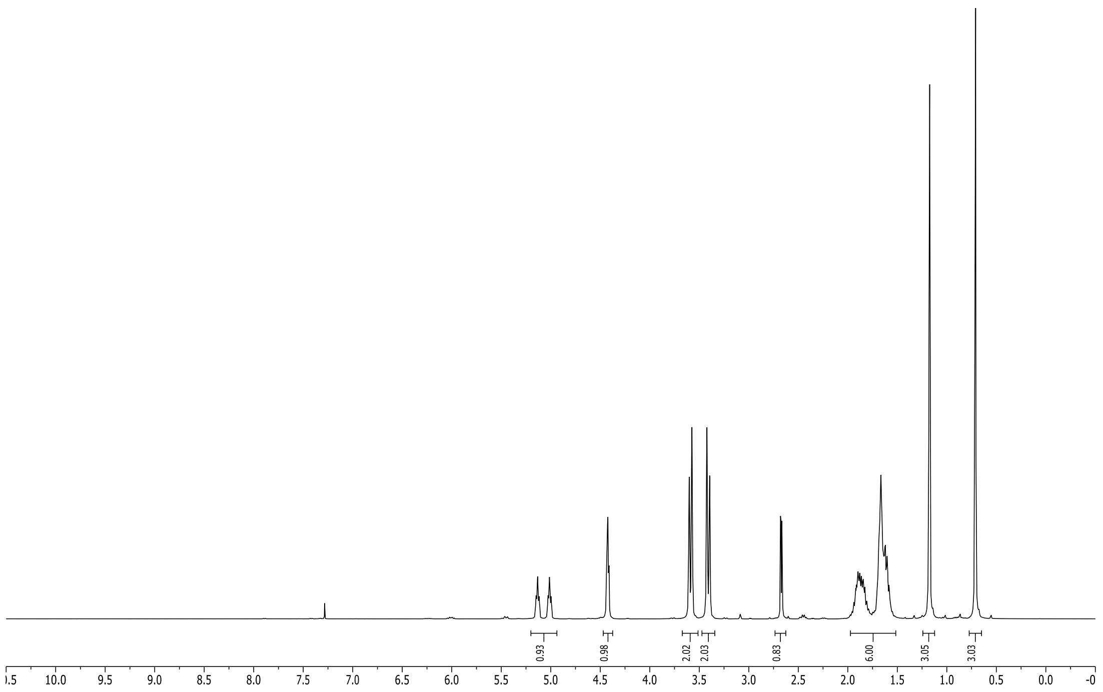

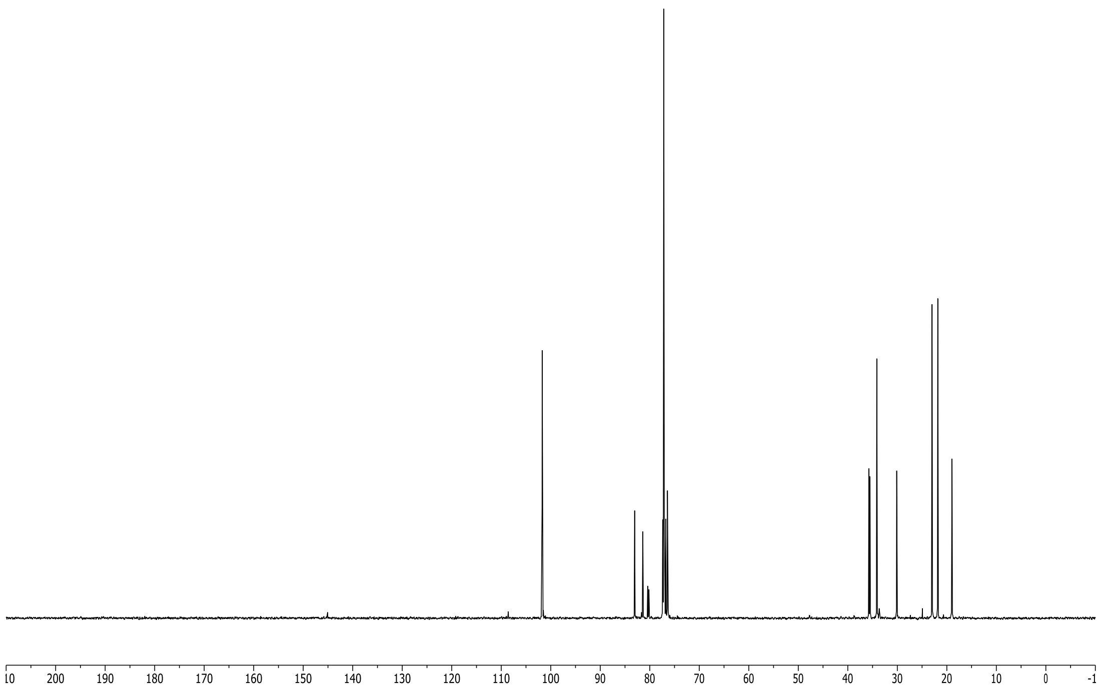

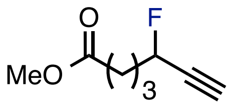

MeO
O
F
3

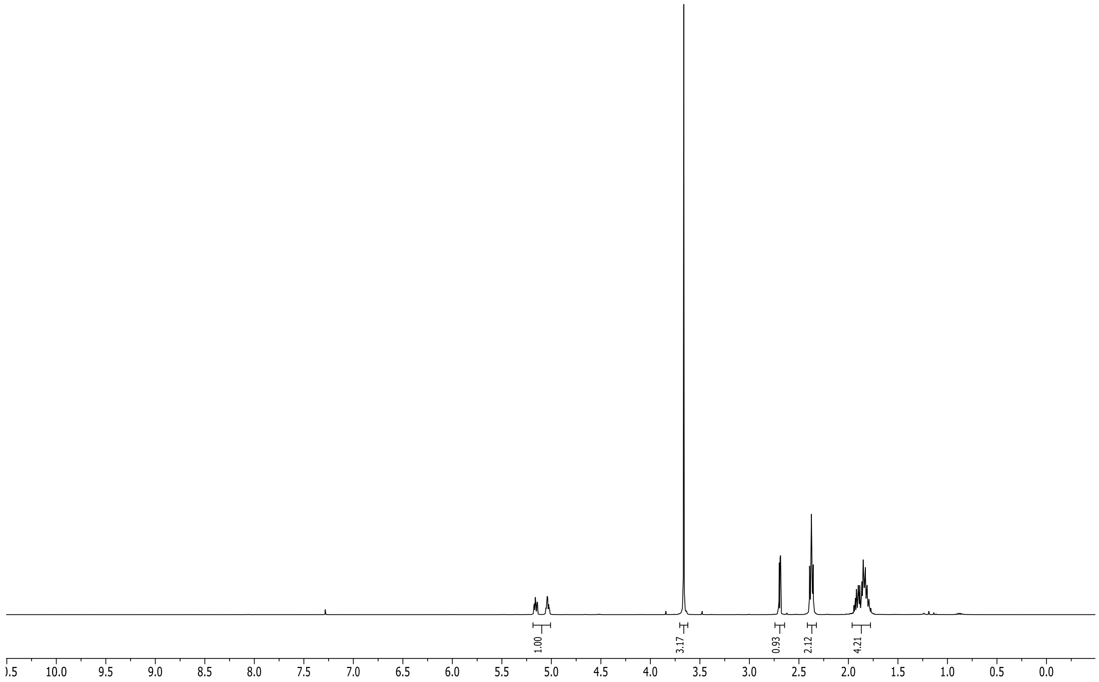

1.00
3.17
0.93
2.12
4.21
10.0 9.5 9.0 8.5 8.0 7.5 7.0 6.5 6.0 5.5 5.0 4.5 4.0 3.5 3.0 2.5 2.0 1.5 1.0 0.5 0.0

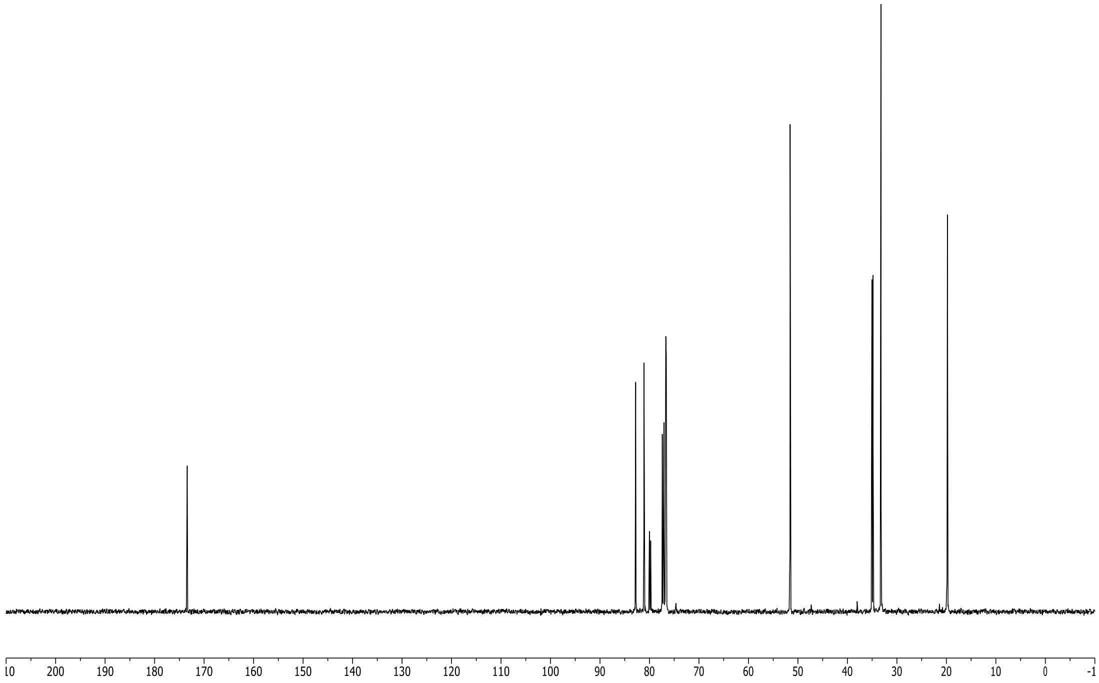

200 190 180 170 160 150 140 130 120 110 100 90 80 70 60 50 40 30 20 10 0

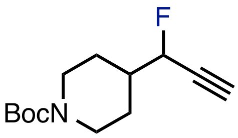
F
BocN

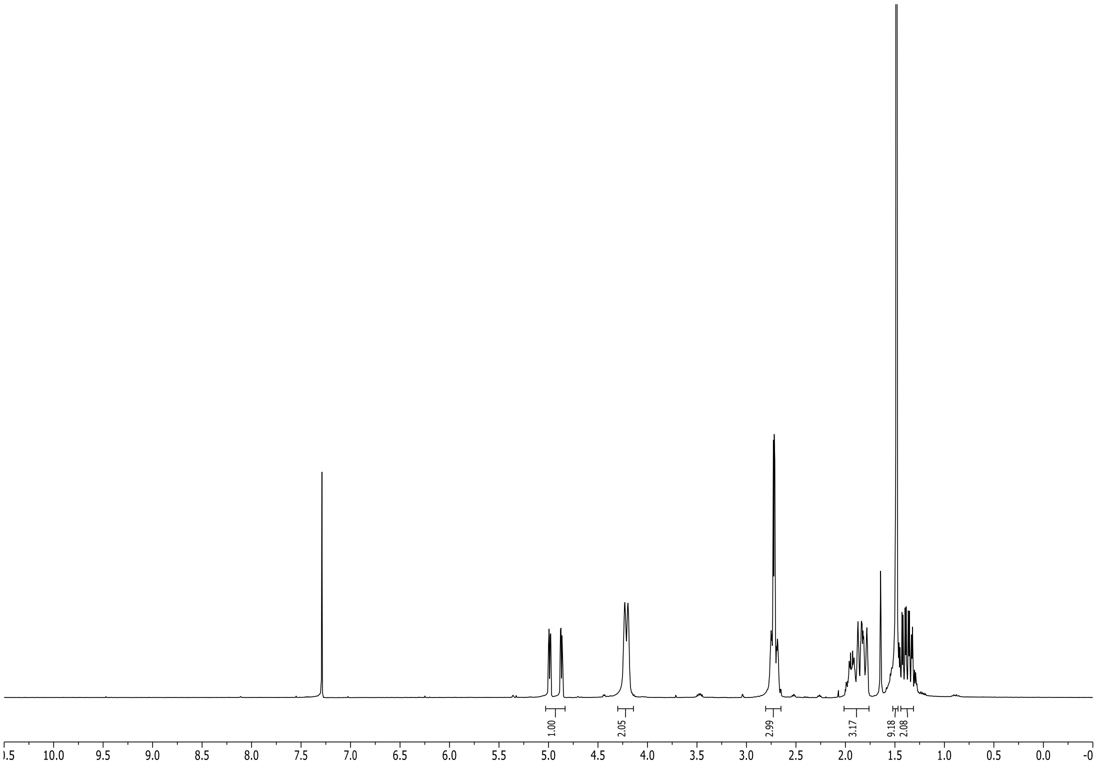

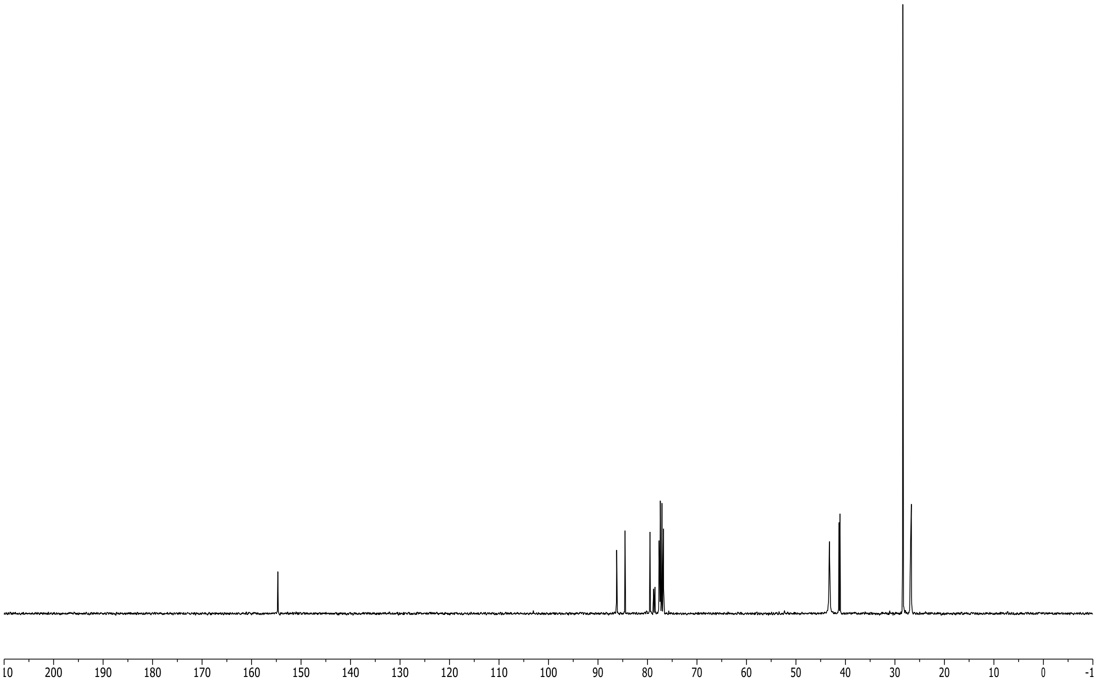

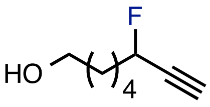
F
HO
4

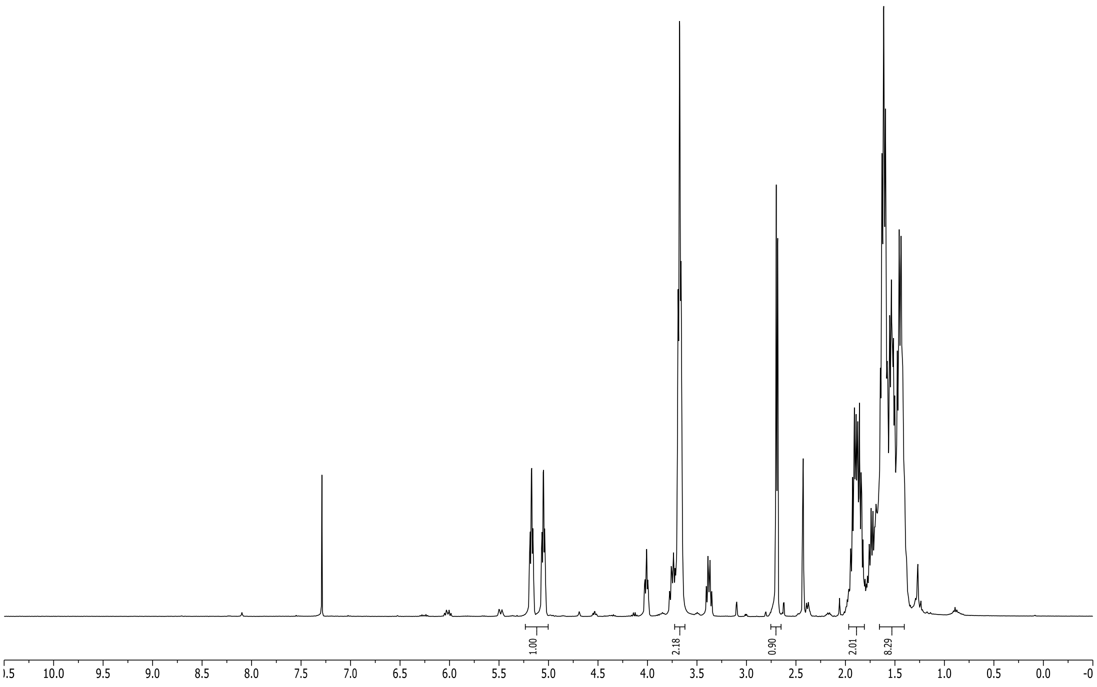
1.00
2.18
0.90
2.01
8.29
10.0
9.5
9.0
8.5
8.0
7.5
7.0
6.5
6.0
5.5
5.0
4.5
4.0
3.5
3.0
2.5
2.0
1.5
1.0
0.5
0.0

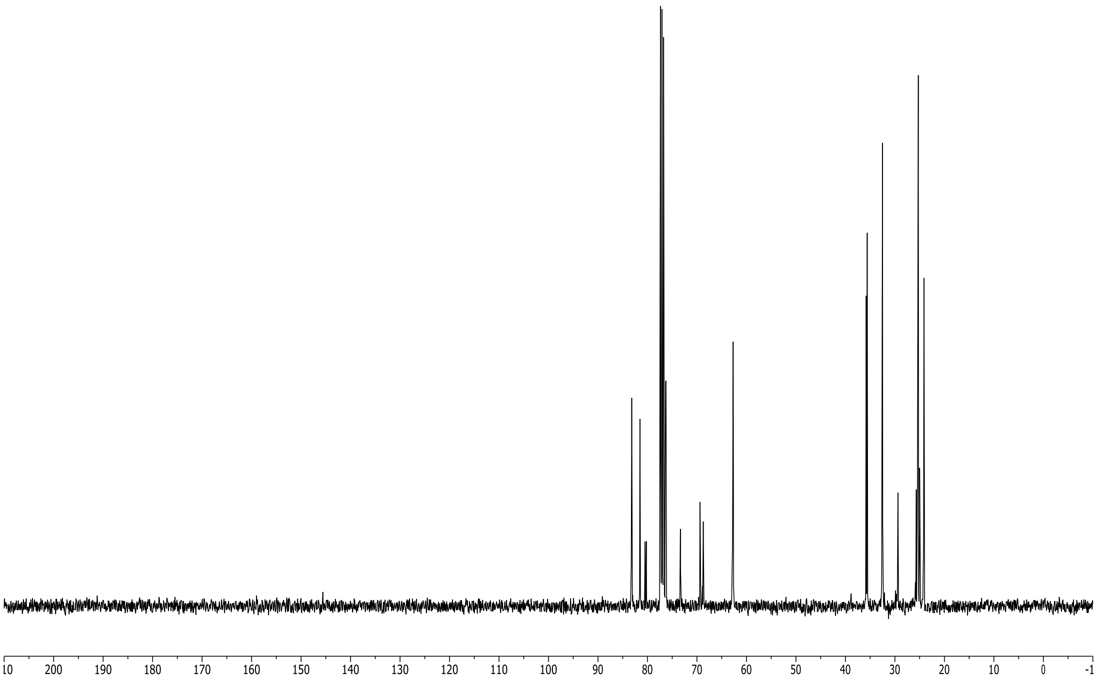
200
190
180
170
160
150
140
130
120
110
100
90
80
70
60
50
40
30
20
10
0

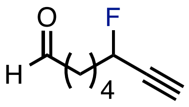
O
F
H
4

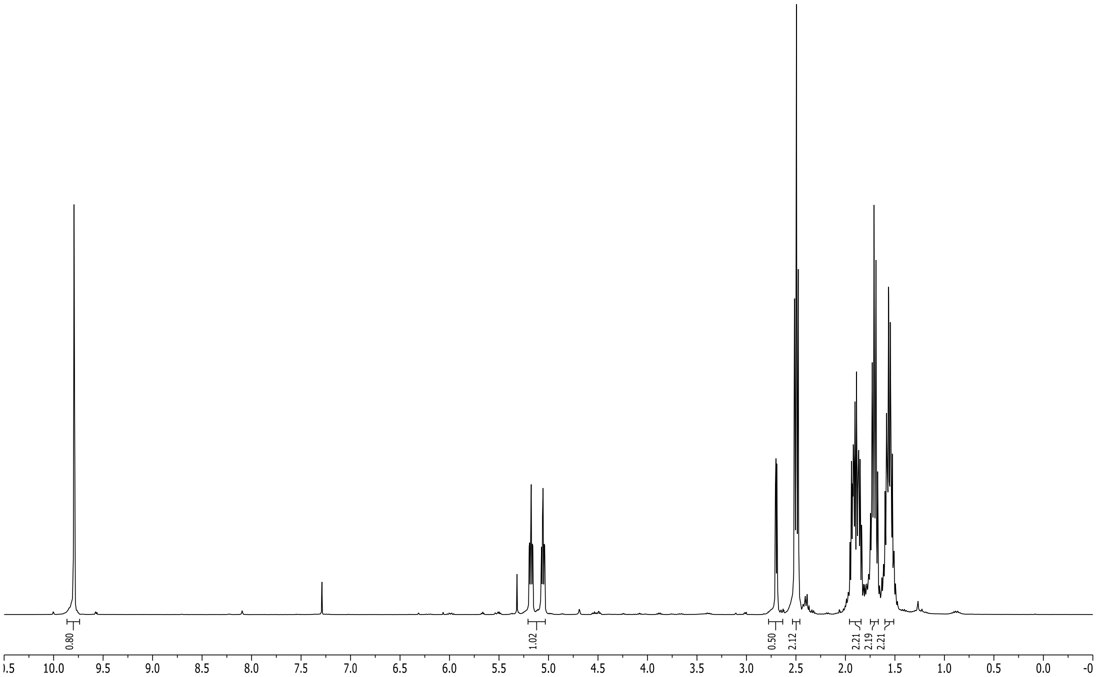
0.80
1.02
0.50
2.12
2.21
2.19
2.21
10.0 9.5 9.0 8.5 8.0 7.5 7.0 6.5 6.0 5.5 5.0 4.5 4.0 3.5 3.0 2.5 2.0 1.5 1.0 0.5 0.0

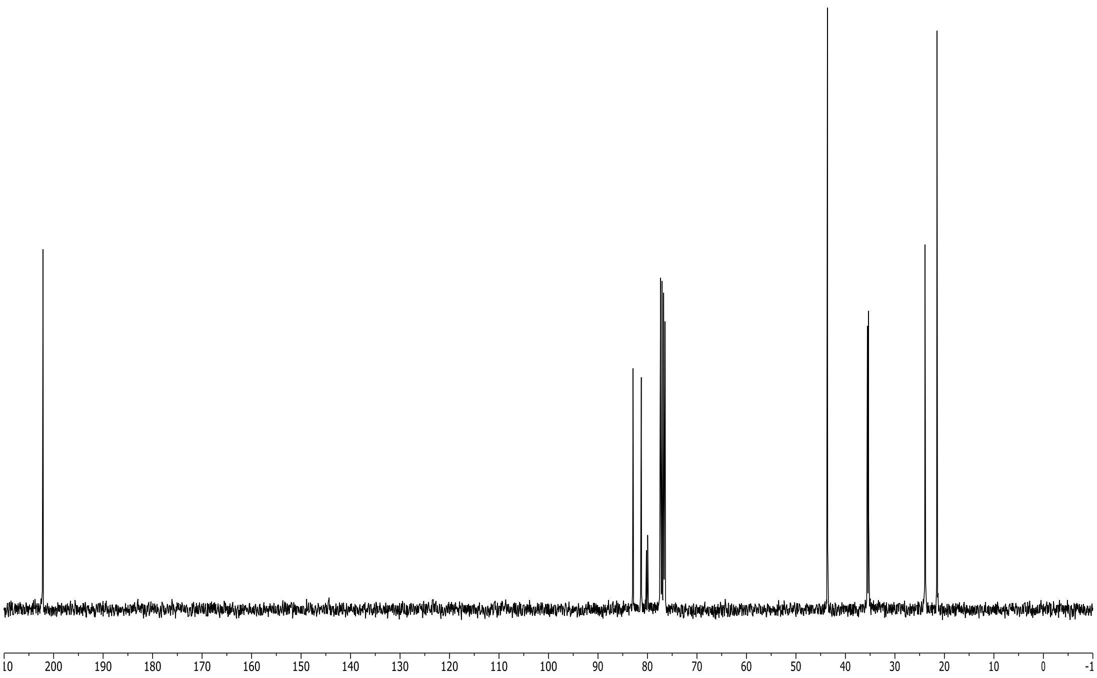
200 190 180 170 160 150 140 130 120 110 100 90 80 70 60 50 40 30 20 10 0

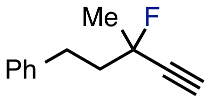

Me
F
Ph

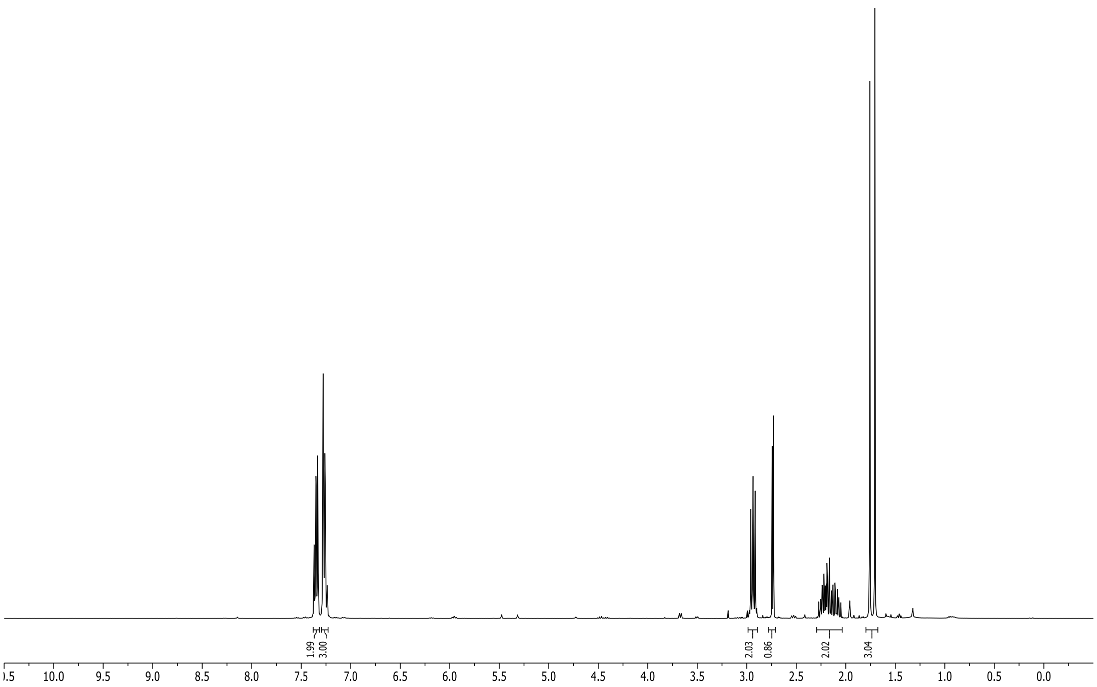

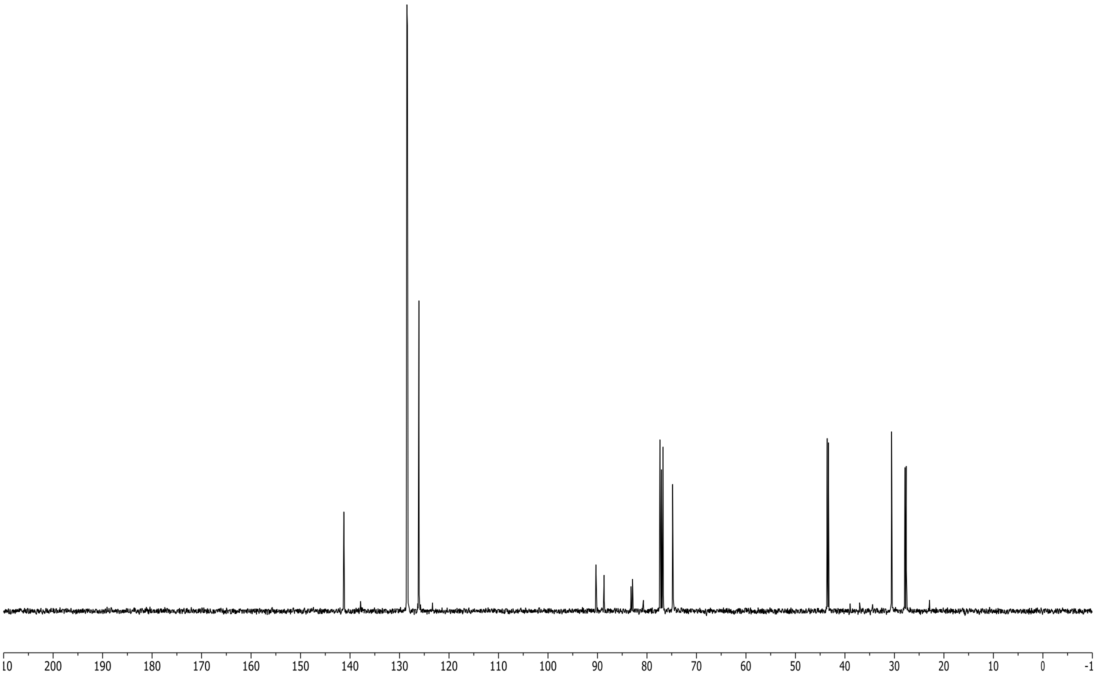

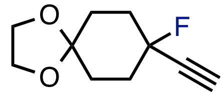
O
O
F

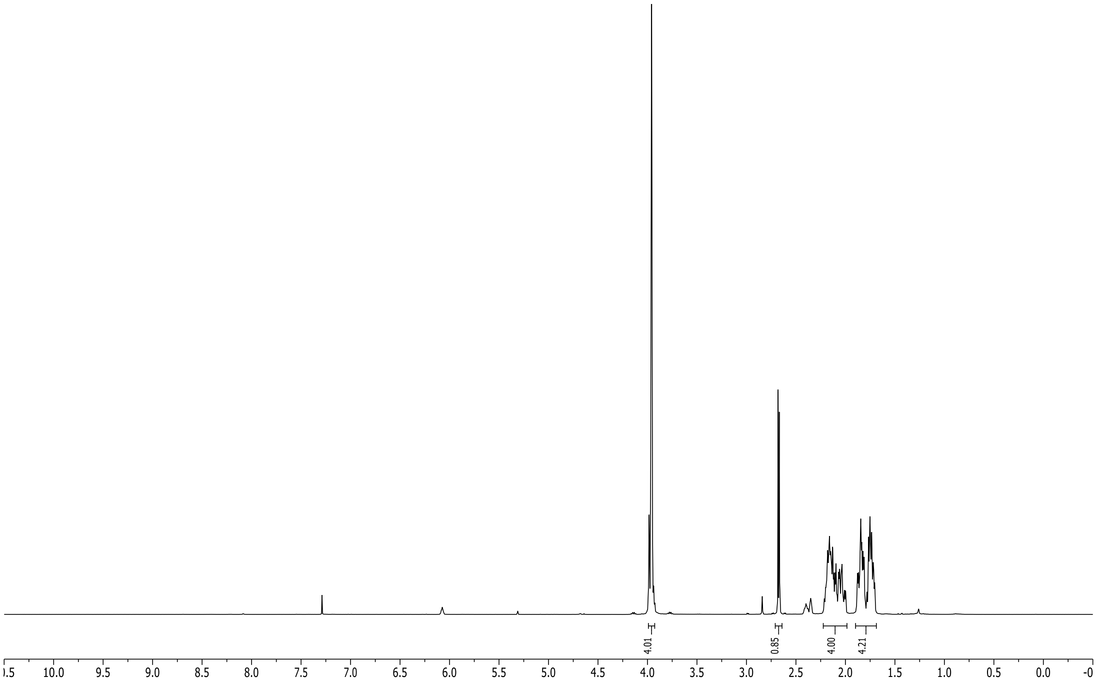
4.01
0.85
4.00
4.21
10.0 9.5 9.0 8.5 8.0 7.5 7.0 6.5 6.0 5.5 5.0 4.5 4.0 3.5 3.0 2.5 2.0 1.5 1.0 0.5 0.0

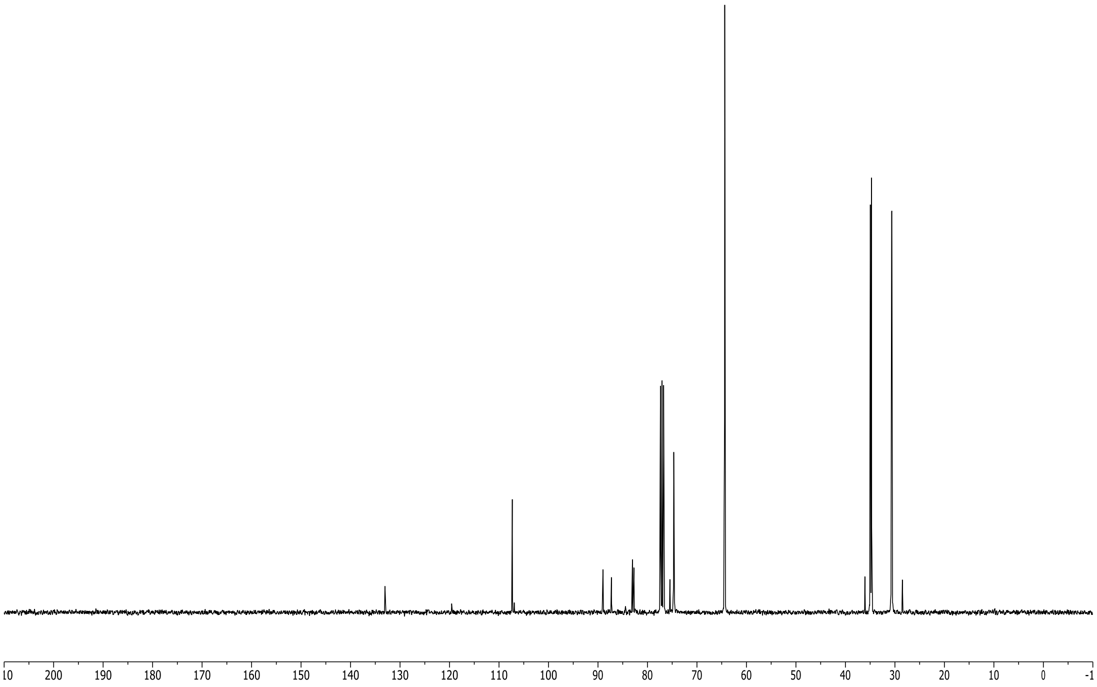
200 190 180 170 160 150 140 130 120 110 100 90 80 70 60 50 40 30 20 10 0

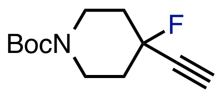
BocN
F

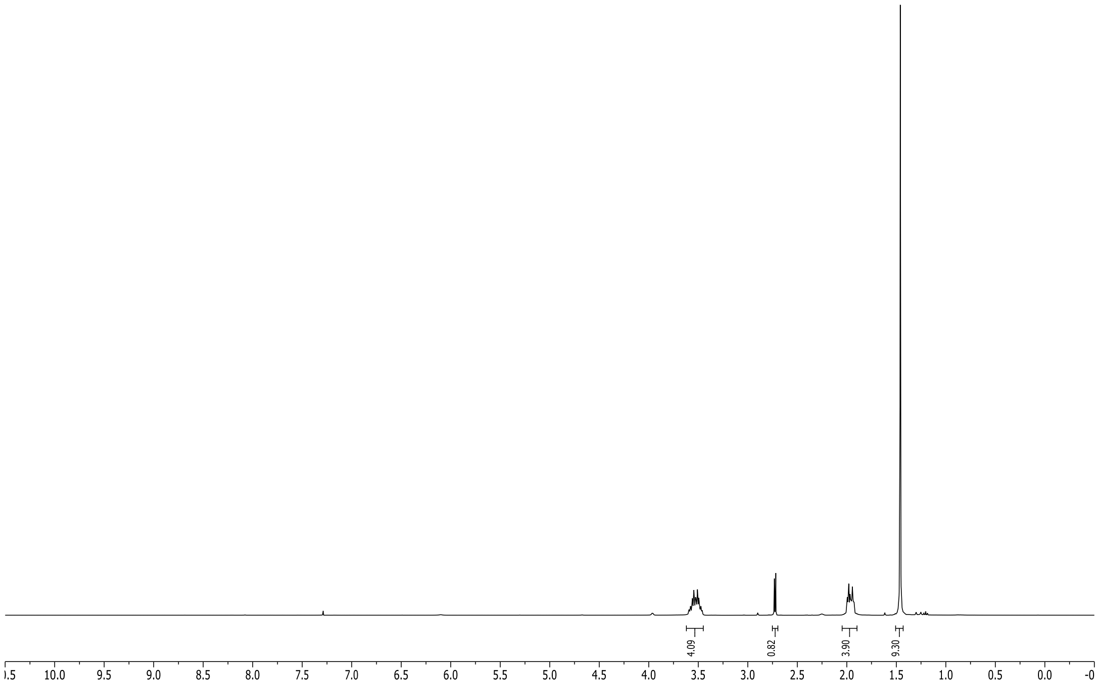
4.09
0.82
3.90
9.30
10.0
9.5
9.0
8.5
8.0
7.5
7.0
6.5
6.0
5.5
5.0
4.5
4.0
3.5
3.0
2.5
2.0
1.5
1.0
0.5
0.0

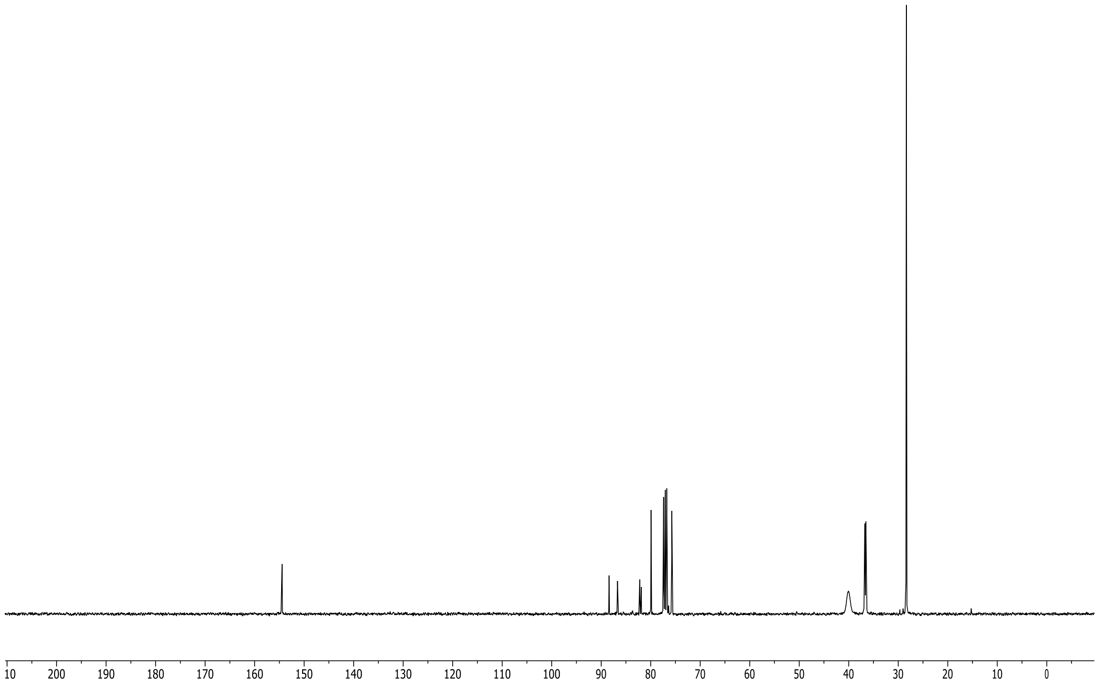
200
190
180
170
160
150
140
130
120
110
100
90
80
70
60
50
40
30
20
10
0

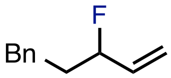
F
Bn

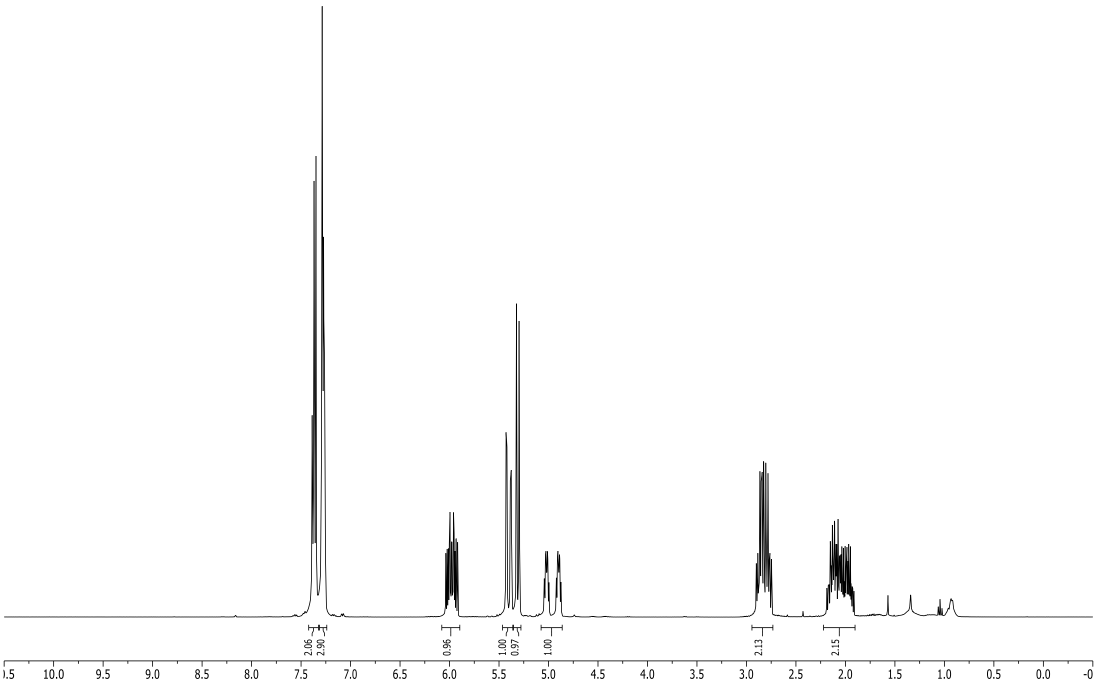
2.06
2.90
0.96
1.00
0.97
1.00
2.13
2.15
10.0 9.5 9.0 8.5 8.0 7.5 7.0 6.5 6.0 5.5 5.0 4.5 4.0 3.5 3.0 2.5 2.0 1.5 1.0 0.5 0.0

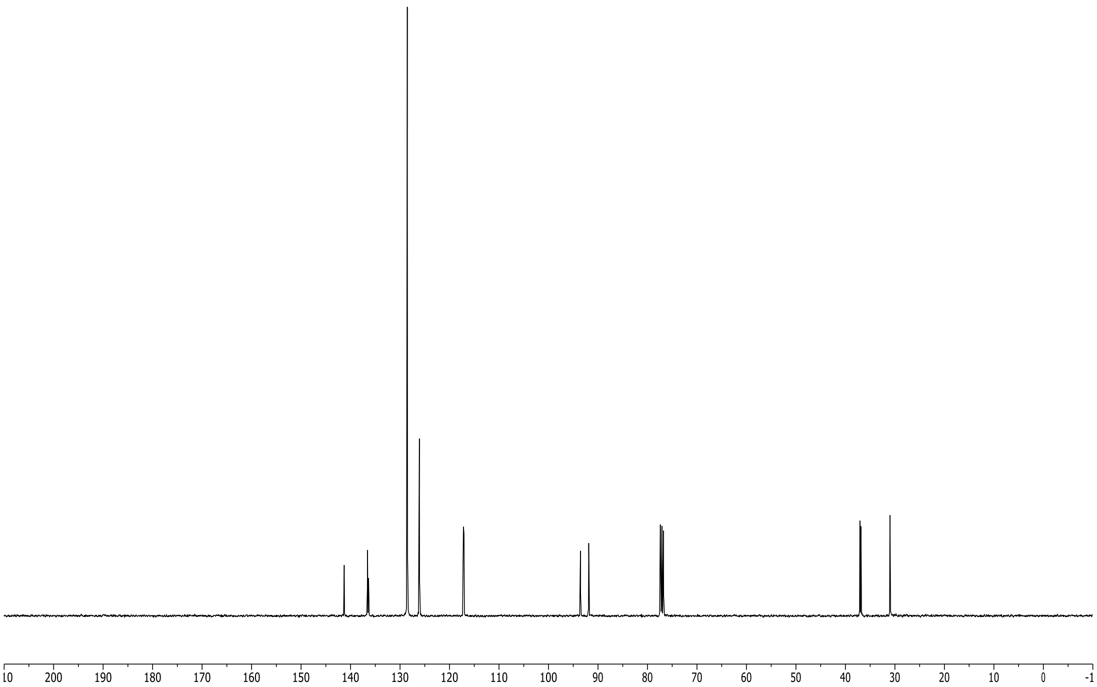
200 190 180 170 160 150 140 130 120 110 100 90 80 70 60 50 40 30 20 10 0

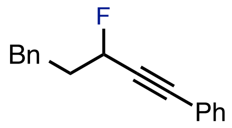
F
Bn
Ph

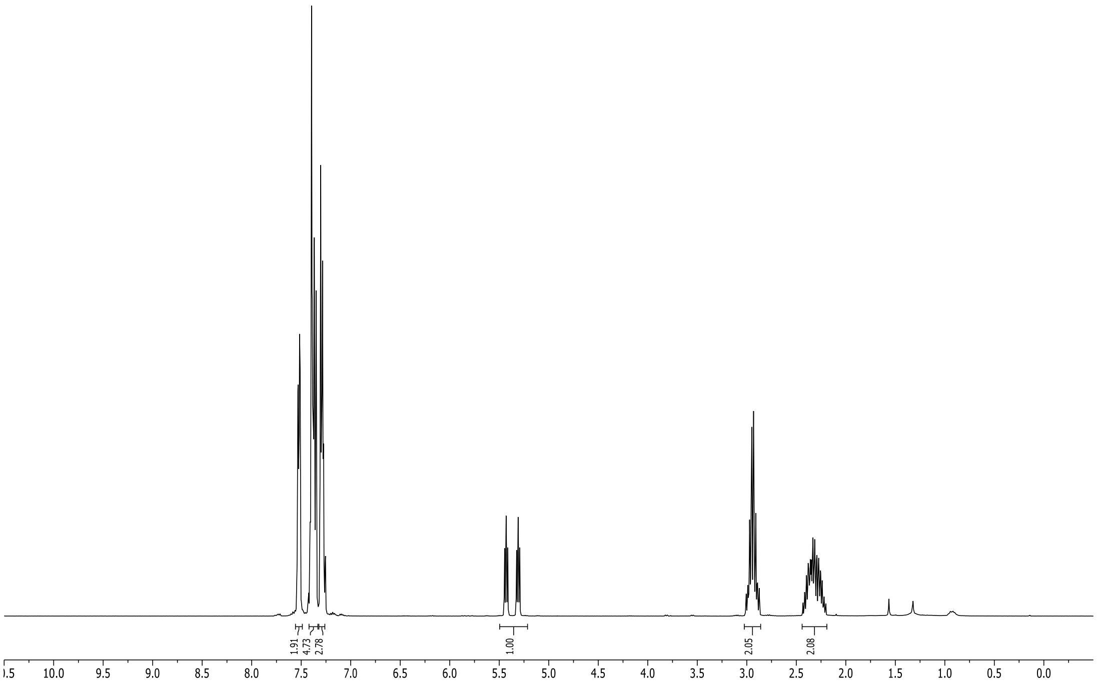
1.91
4.73
2.78
1.00
2.05
2.08
10.0 9.5 9.0 8.5 8.0 7.5 7.0 6.5 6.0 5.5 5.0 4.5 4.0 3.5 3.0 2.5 2.0 1.5 1.0 0.5 0.0

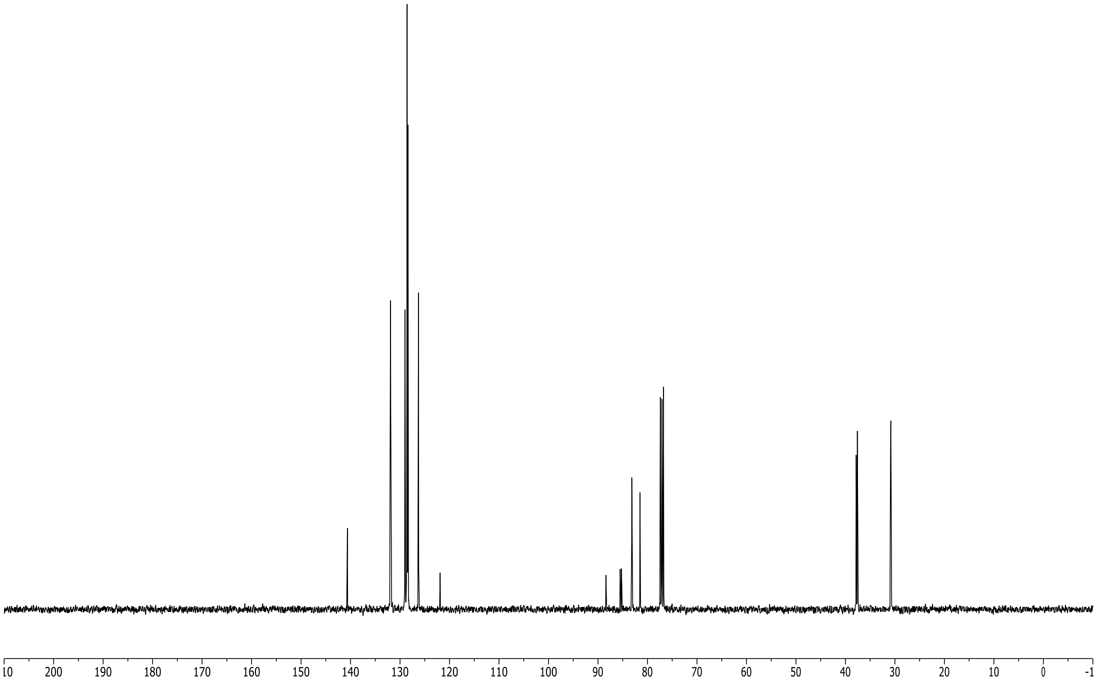
200 190 180 170 160 150 140 130 120 110 100 90 80 70 60 50 40 30 20 10 0

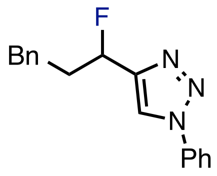
F
Bn
N
N
N
Ph

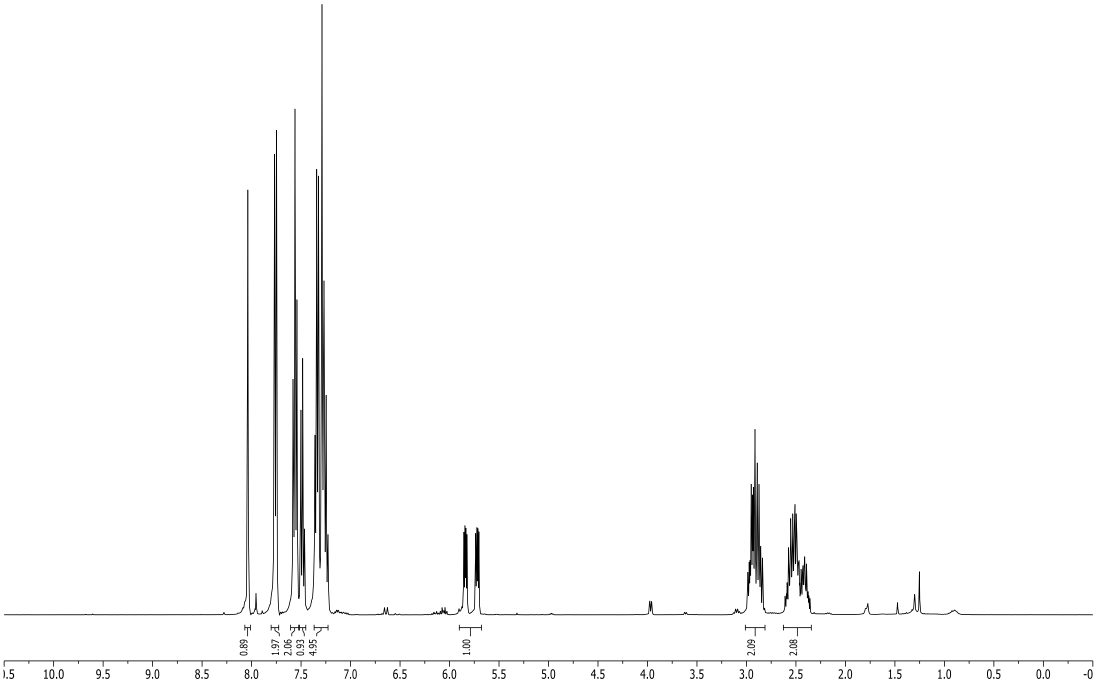
0.89
1.97
2.06
0.93
4.95
1.00
2.09
2.08
10.0 9.5 9.0 8.5 8.0 7.5 7.0 6.5 6.0 5.5 5.0 4.5 4.0 3.5 3.0 2.5 2.0 1.5 1.0 0.5 0.0

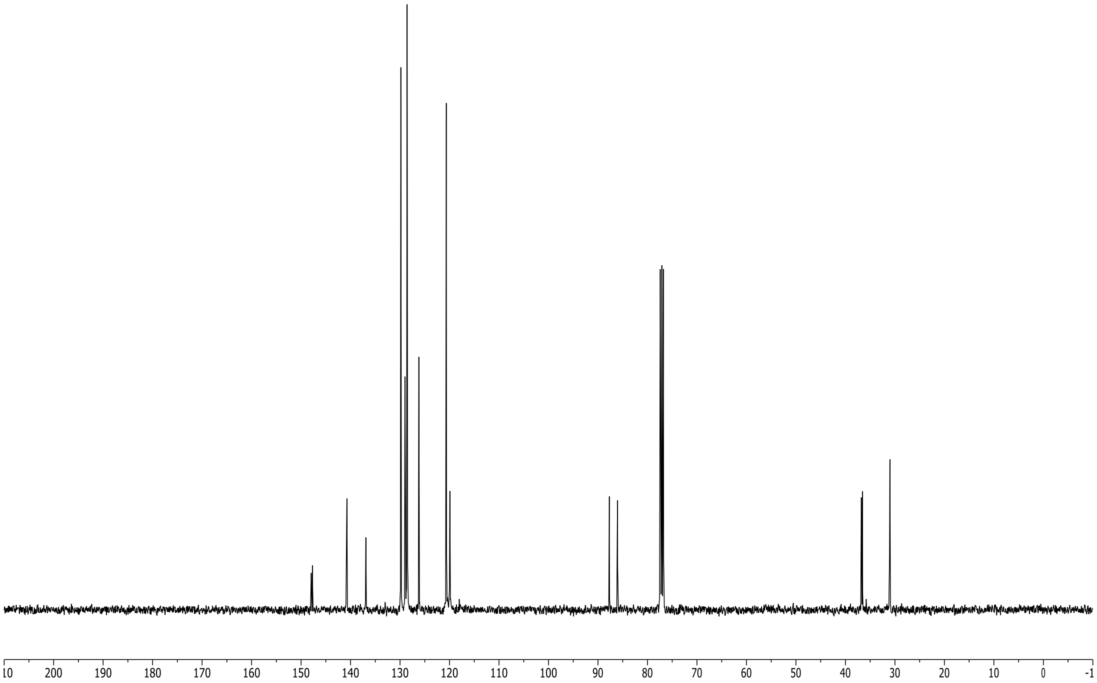
200 190 180 170 160 150 140 130 120 110 100 90 80 70 60 50 40 30 20 10 0

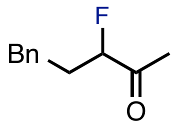
F
Bn
O

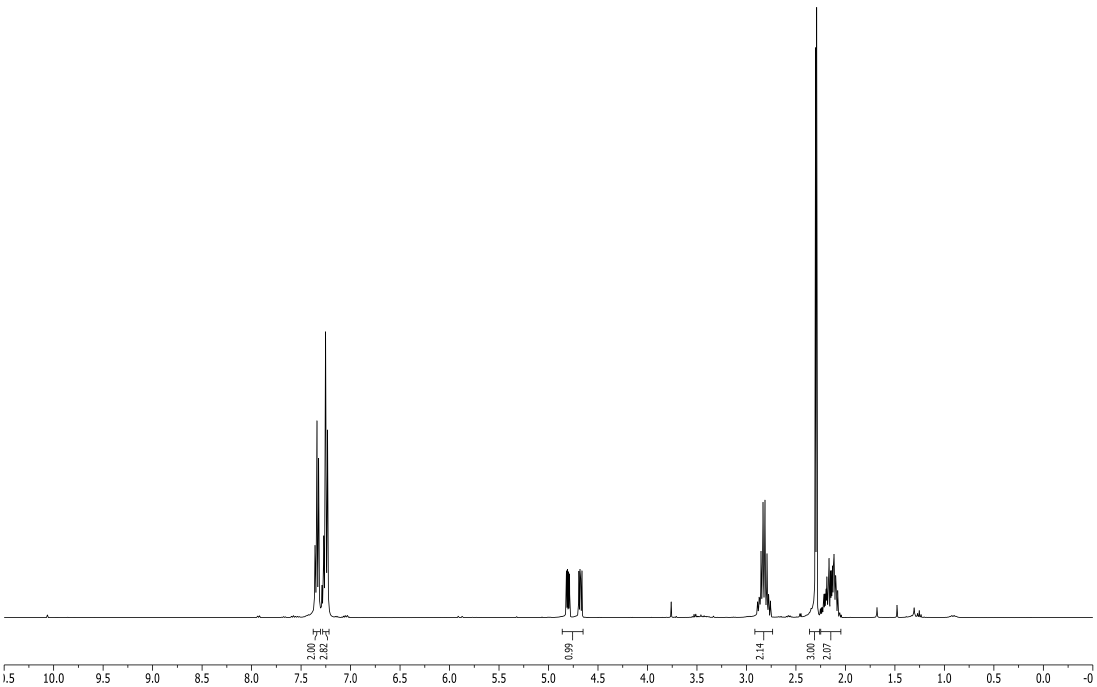
2.00
2.82
0.99
2.14
3.00
2.07
10.0
9.5
9.0
8.5
8.0
7.5
7.0
6.5
6.0
5.5
5.0
4.5
4.0
3.5
3.0
2.5
2.0
1.5
1.0
0.5
0.0

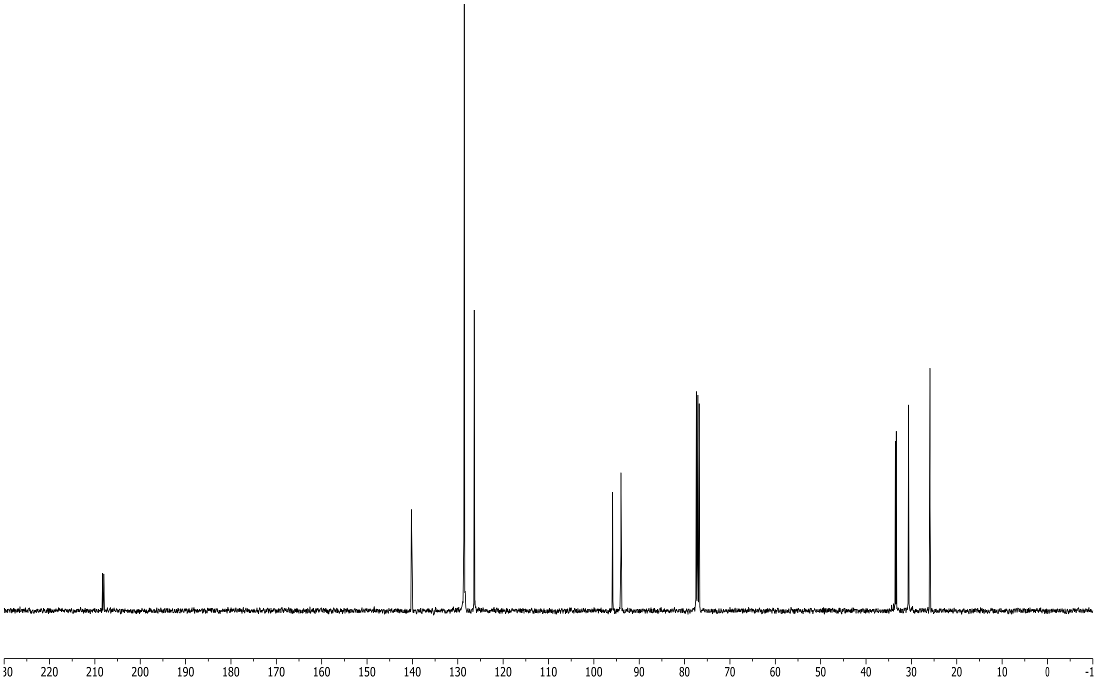
220
210
200
190
180
170
160
150
140
130
120
110
100
90
80
70
60
50
40
30
20
10
0

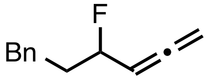
F
Bn

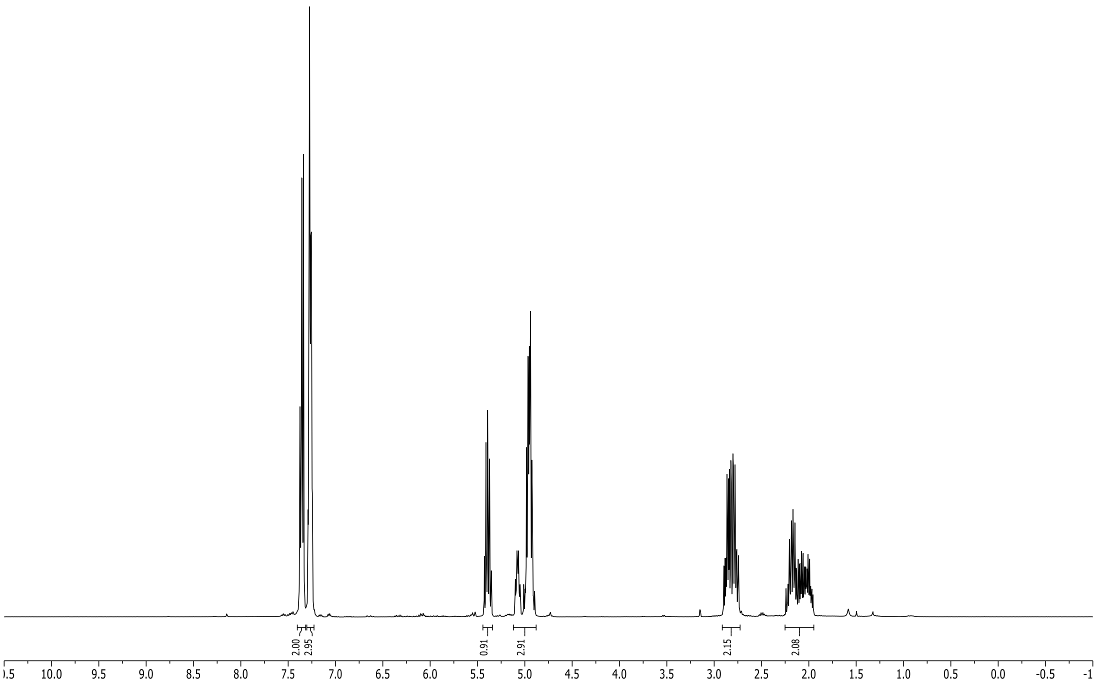
2.00
2.95
0.91
2.91
2.15
2.08
10.0 9.5 9.0 8.5 8.0 7.5 7.0 6.5 6.0 5.5 5.0 4.5 4.0 3.5 3.0 2.5 2.0 1.5 1.0 0.5 0.0 -0.5

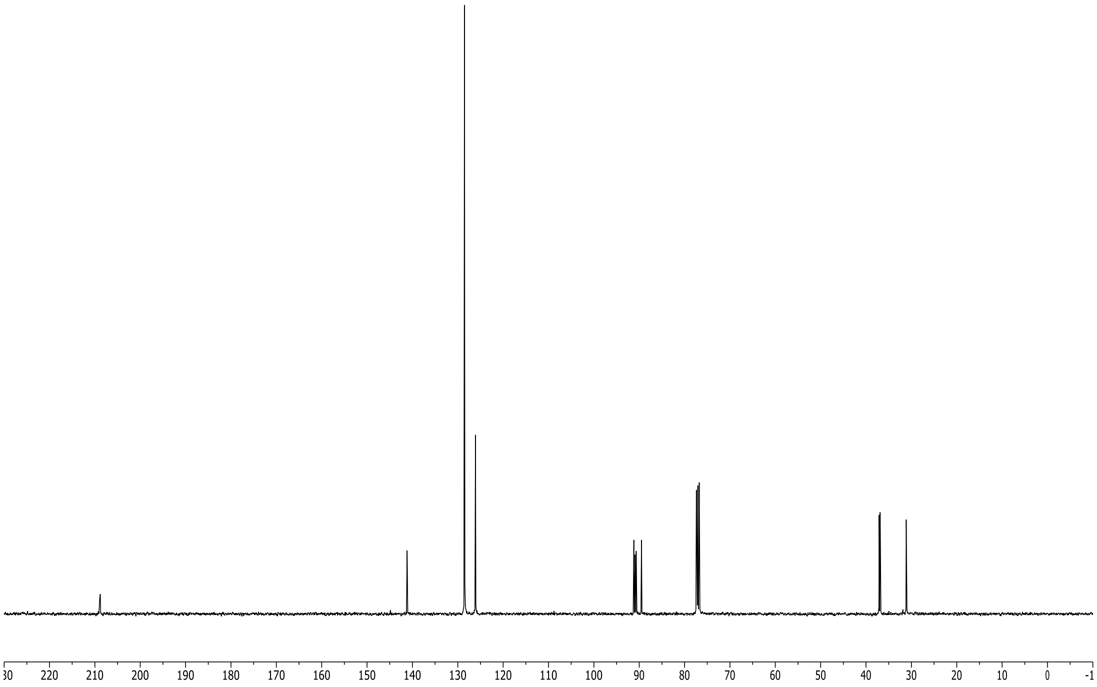
220 210 200 190 180 170 160 150 140 130 120 110 100 90 80 70 60 50 40 30 20 10 0

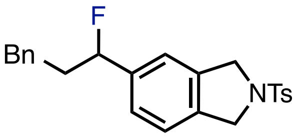
F
Bn
NTs

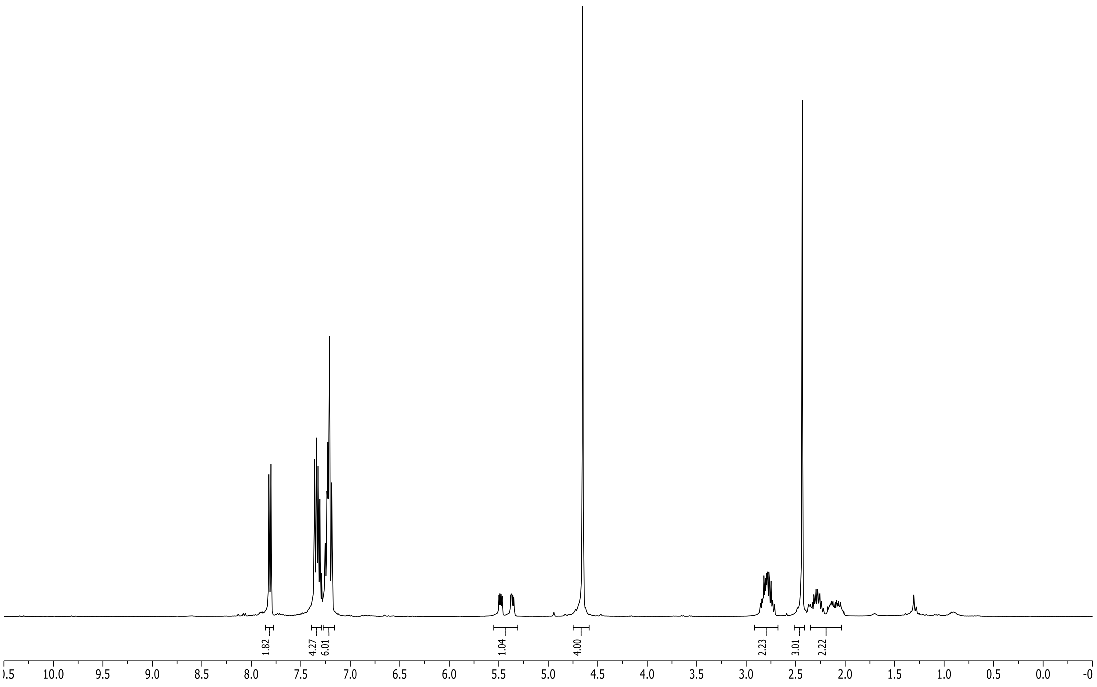
1.82
4.27
6.01
1.04
4.00
2.23
3.01
2.22
10.0 9.5 9.0 8.5 8.0 7.5 7.0 6.5 6.0 5.5 5.0 4.5 4.0 3.5 3.0 2.5 2.0 1.5 1.0 0.5 0.0

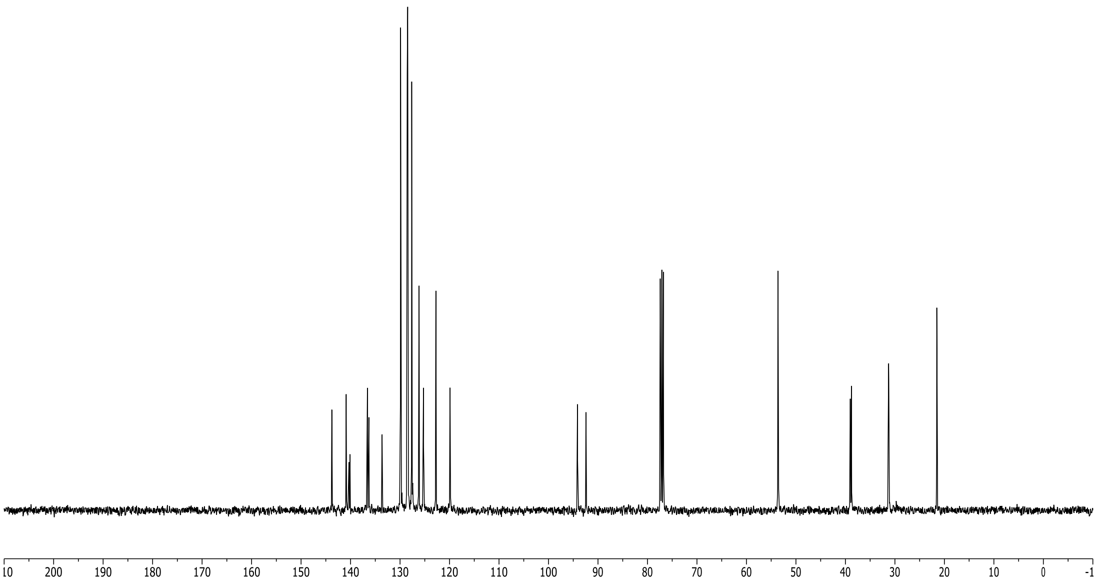
200 190 180 170 160 150 140 130 120 110 100 90 80 70 60 50 40 30 20 10 0
